# Supplementary material for: Protein context shapes the specificity of SH3 domain-mediated interactions in vivo
Source: Nat Commun. 2021 Mar 12;12:1597. doi: 10.1038/s41467-021-21873-2 (PMC7954794; doi:10.1038/s41467-021-21873-2)
Supplement: Supplementary file 1 — Supplementary Information [file 41467_2021_21873_MOESM1_ESM.pdf]

Supplementary Information

Protein context shapes the specificity of SRC-Homology 3 (SH3) domain-mediated interactions in vivo

Dionne, *et al*

This PDF file includes:

Supplementary Figures 1-7

Supplementary Tables 1-3

Supplementary References (1-4)

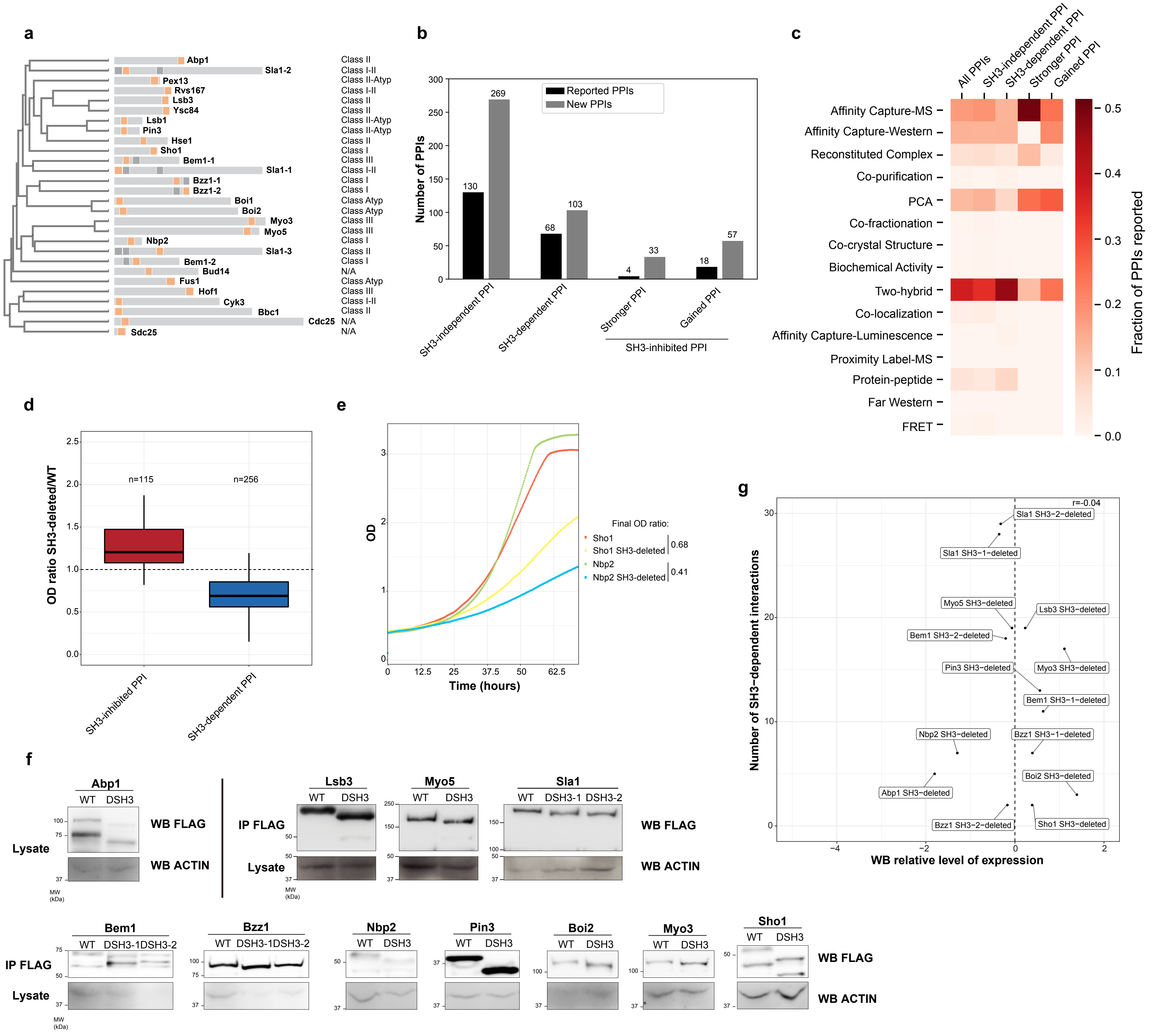

Supplementary Fig. 1| Yeast SH3 domains, the number and types of PPI changes in response to SH3 deletion and validation. a The relationships of yeast SH3 amino acid sequences. Domain position and length relative to their host proteins are illustrated. SH3s used in the phylogeny are shown in orange. Grey squares are other SH3s in the same protein. The preferred type of binding motifs (Atyp stands for atypical) from in vitro assays are indicated[1]. **b** The proportion of PPIs previously reported for the different types of PPIs affected or not by SH3 deletion[2]. **c** The fraction of known PPIs per method of detection is shown for the same categories of interactions as in b[2]. **d** Validation of the PPIs altered by the deletion of yeast SH3s using low-throughput liquid DHFR-PCA. The ratios (SH3-deleted/WT) of the optical density (OD) from the last time point of the experiment for the growth curves of each PPI are shown. A ratio higher than 1 represents an interaction that is stronger upon SH3 deletion. The median is represented as a bold center line and hinges are for the 25th and 75th percentiles (first and third quartiles). Whiskers extend from the hinges to maximum 1.5 times the Q3-Q1 interquartile range. **e** Examples of two SH3-dependent PPIs DHFR-PCA liquid assay. Growth curves are shown for the WT and SH3-deleted baits for Sho1-Pbs2 and Nbp2-Pbs2 PPIs. The ratios of the final optical density (SH3-deleted/WT) as represented in panel b are also indicated. **f** Western blot (WB) analysis of the expression level of SH3-deleted proteins that lost many PPIs upon their SH3-deletion. All baits have a C-terminal 1xFLAG tag allowing their immuno-detection. The WB validation of the expression level of the baits was performed in one replicate. **g** Comparison of the relative level of expression of SH3-deleted baits quantified by WB (log2 of SH3-deleted/WT ratio, WB relative level of expression of 0 for WT baits) and their number of SH3-dependent partners identified by DHFR-PCA (Pearson's r = -0.04, p-value = 0.90, two-sided). Source data are provided as a Source Data file. See also Supplementary Data 1.

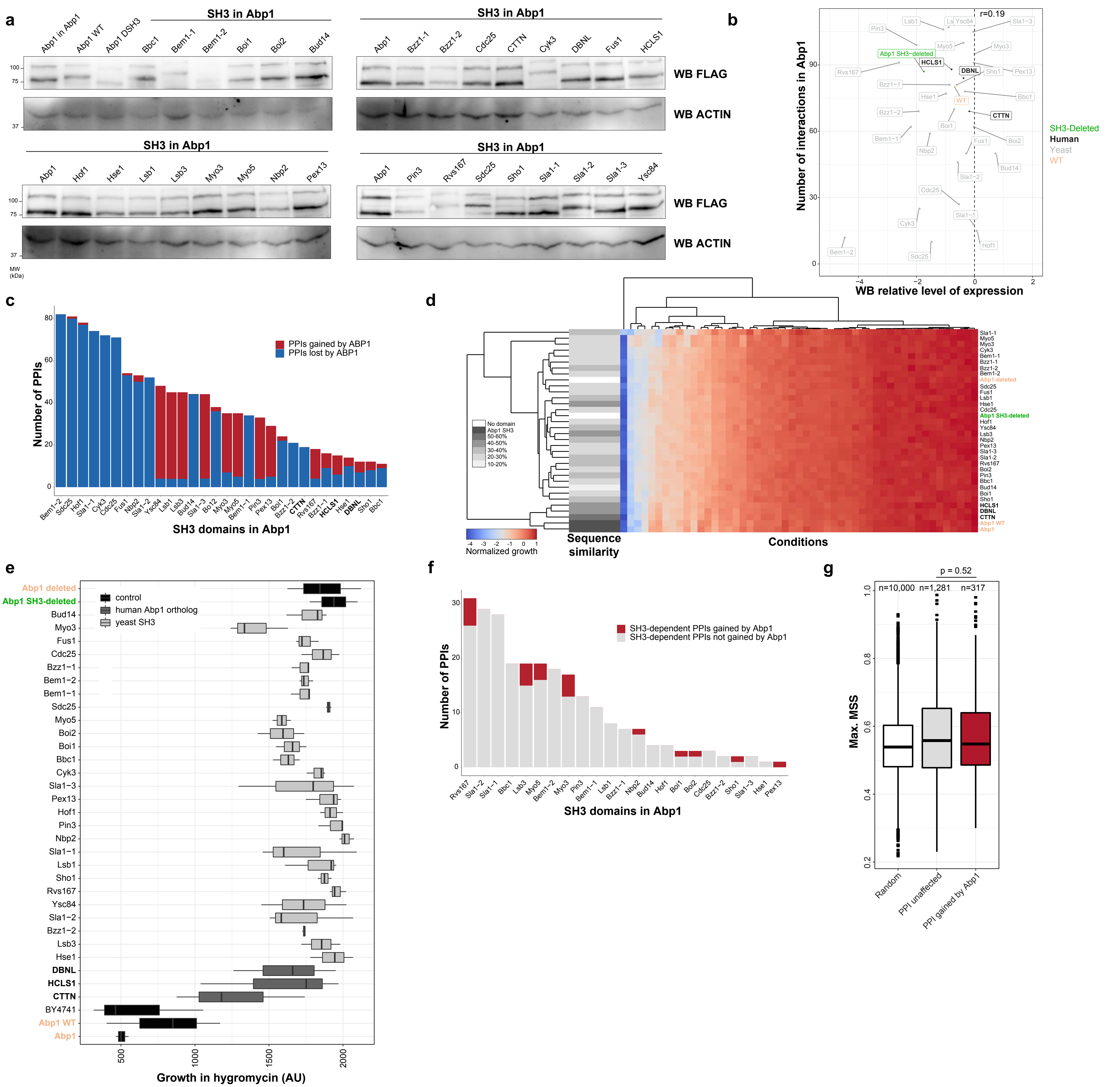

Supplementary Fig. 2| Characterization of Abp1 SH3 domain swapping. a WB analysis of protein expression levels of all Abp1 SH3 swapped bait proteins as compared to the WT Abp1, Abp1SH3 in Abp1 and SH3-deleted control baits. Two bands are detected for Abp1, as previously observed[3]. The WB validation of the expression level of every bait was performed in two replicates. **b** The relative level of expression of Abp1 SH3-swapped baits quantified by WB (log2 of Abp1 SH3 swapped/Abp1SH3 in Abp1 ratio, WB relative level of expression of 0 for the Abp1SH3 in Abp1 bait) compared to their number of PPIs as identified by DHFR-PCA (Pearson's r = 0.19, p-value = 0.31, two-sided). **c** Number of PPIs that were affected by Abp1 SH3 domain swapping. **d** Growth in stress conditions for the Abp1 SH3 swapped strains. The growth values were scaled per strain (row). Blue to red represents the normalized growth per strain after 74 hours (log2 colony size). The sequence similarity of each SH3 to Abp1 SH3 is represented in grey scale. Each strain was grown in twelve replicates. **e** Growth of Abp1 SH3 swapped stains in liquid medium with hygromycin is represented (area under the curve (AU)). The control strains, shown in black, highlight that the resistance to the drug is dependent on Abp1 SH3. Domains are sorted by increasing sequence similarity with Abp1 from the top. Growth rates were measured in triplicates. **f** Number of SH3-dependent PPIs that were gained by each SH3 swapped to Abp1 (as determined in Figure 1b) in comparison to the ones not gained. **g** PWMs analysis of PPIs gained by Abp1 using the matrix similarity scores (MSS) between the PWM and sequence. This analysis shows no enrichment of the predicted SH3 motifs in the gained PPIs relative to the unaffected Abp1 PPIs (p = 0.52, Mann-Whitney test, one-sided). For c, and d, the orange SH3s are controls and the SH3-deleted protein is in green. In all panels, human SH3s are shown in bold. For every boxplot, the median is represented as a bold center line and hinges are for the 25th and 75th percentiles (first and third quartiles). Whiskers extend from the hinges to maximum 1.5 times the Q3-Q1 interquartile range. For g, outliers are represented as black dots. Source data are provided as a Source Data file. See also Supplementary Data 1 and Supplementary Data 2.

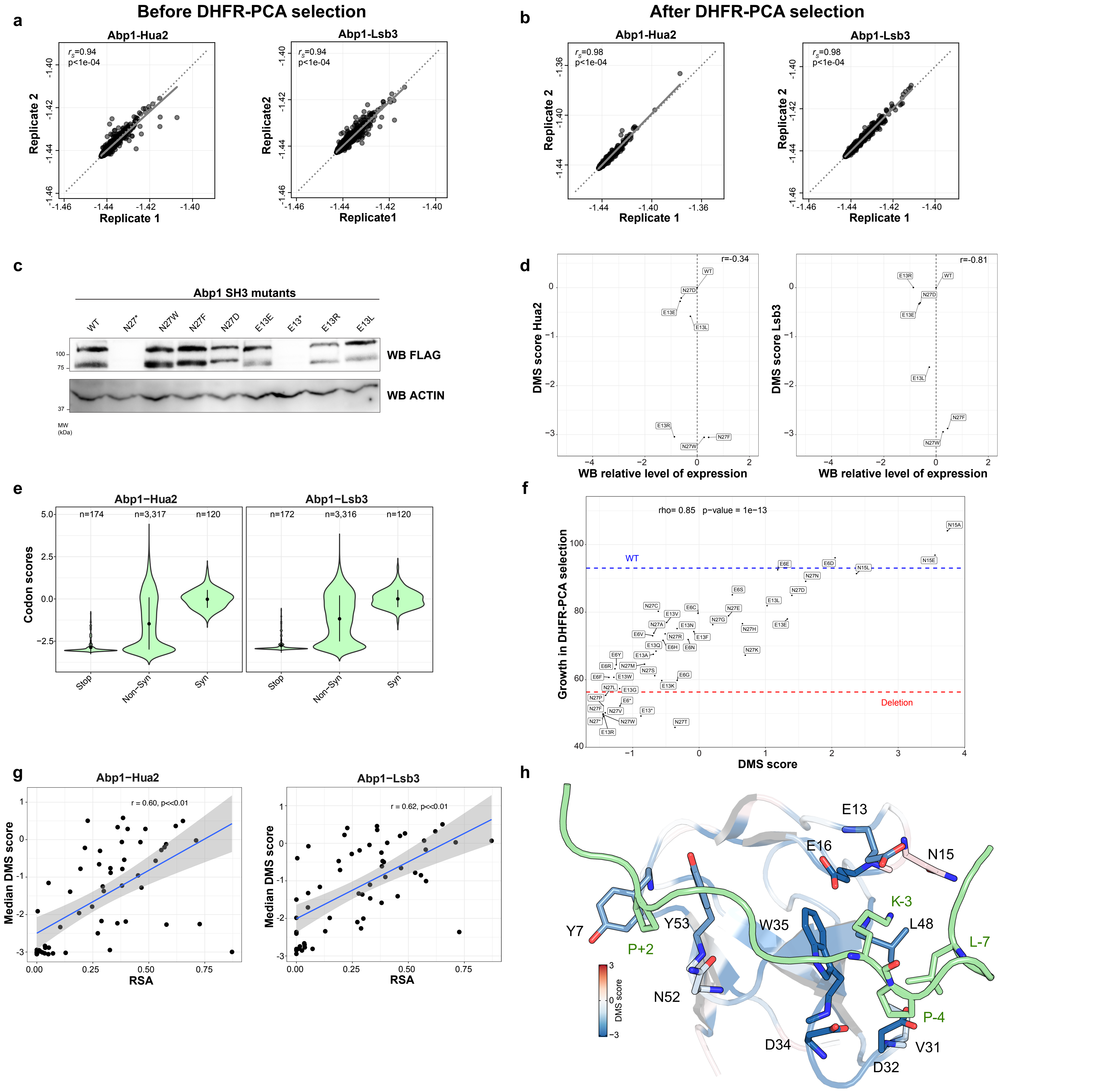

Supplementary Fig. 3| Characterization and validation of DMS mutants of Abp1 SH3 domain. a-b Reproducibility between biological replicates of DMS DHFR-PCA experiments. The correlations for the two interactors are shown (a is for the reference condition and b for the DHFR-PCA condition). The frequencies of mutants (shown on log2 scale) were compared in terms of Spearman's rank correlation coefficient (rs). Associated p-value for a test whose null hypothesis is that two variables are uncorrelated, is shown on each plot. **c** WB comparing expression levels of selected Abp1 SH3 mutants with WT Abp1. An asterisk represents a stop codon, which inhibits translation of the 1xFLAG epitope. The WB validation of the expression level of selected Abp1 SH3 mutants was performed in two replicates. **d** Quantification of the relative level of expression of Abp1 SH3 mutants by WB as in c (log2 of Abp1 SH3 mutant/Abp1 WT ratio, WB relative level of expression of 0 for WT Abp1) compared to their DMS scores (two-sided tests: Hua2 (left): Pearson's r = -0.34, p-value = 0.46, Lsb3 (right): Pearson's r = -0.81, p-value = 0.03). **e** Distribution of the codon log2 average sequence counts for each type of mutation of Abp1 SH3 for the two PPIs. Syn is for synonymous, error bar represents the mean plus and minus one standard deviation. **f** Selected non-conserved positions (E6, E13, N15 and N27) sensitivities to mutations for the Abp1-Hua2 PPI were tested in a low-throughput liquid DHFR-PCA assay. The growth of the mutants in the low-throughput experiment (average of four replicates of the total OD for each growth curve, y axis, n = 45 mutants) is compared to the mutants DMS score (x axis). The growth of WT Abp1 and Abp1 SH3-deleted are represented by horizontal dash lines. **g** The median DMS score of the 58 different Abp1 SH3 positions for both PPIs in relation to their relative solvent accessibility (RSA). Shaded area represents the 95% confidence interval for linear model predictions. **h** Abp1 SH3 residues in close proximity (within 4 Å) to the most important binding residues of the Ark1 peptide (P+2, K-3, P-4, and L-7) are mapped on the structure of Abp1 SH3 in complex with the peptide (PDB: 2RPN[4]). Ark1 peptide is shown in green, oxygen atoms are in red and nitrogen atoms are in blue. The colors of Abp1 SH3 residues that are mapped on the structure represent their average DMS score for Abp1-Hua2 PPI. Most interface residues sensitive to mutations, such as Y7, E13, E16, V31, D32, D34, L48 and Y53, specifically affect Hua2 PPI. Source data are provided as a Source Data file. See also Supplementary Data 1.

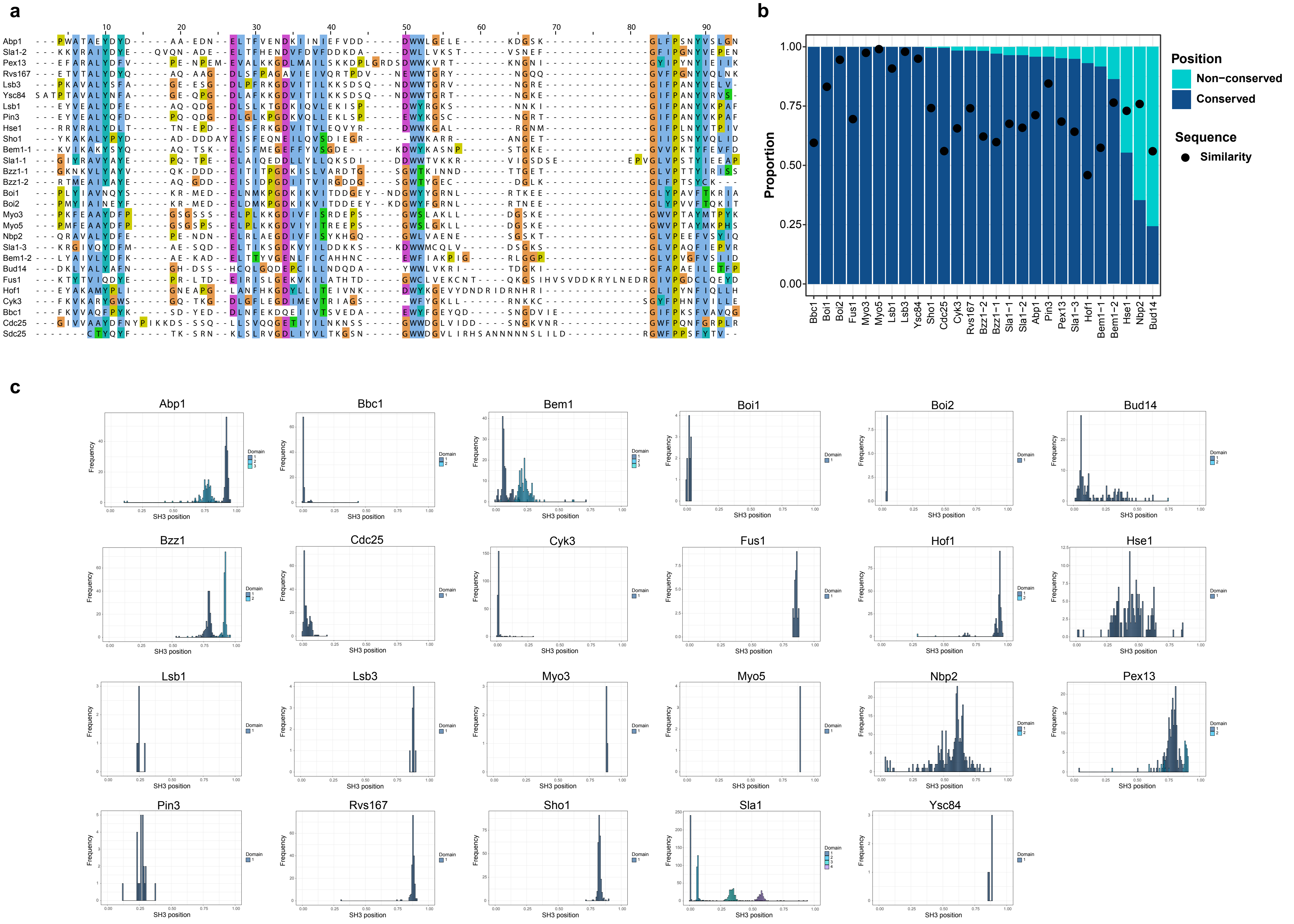

Supplementary Fig. 4| Yeast SH3 domain sequence and position in their host protein. a Multiple sequence alignment of yeast SH3 domains. **b** The y-axis shows the fraction of one-to-one orthologs (across 449 fungal species) with conserved domain positions relative to *S. cerevisiae*. The mean sequence similarity is represented by black circles. The pseudogene Sdc25 was excluded from these analyses. **c** Each panel represents the distribution of SH3 domain positions for the one-to-one orthologs of a given *S. cerevisiae* SH3. For each ortholog sequence, SH3 domain start positions were identified from SH3 hidden Markov models (HMMs), and then the start positions compiled across all orthologs to derive a frequency distribution. The SH3 domains are numbered in cases where more than one SH3 was identified across orthologs, where ‘1’ represents the domain that is also found in the *S. cerevisiae* copy. For multi-SH3 proteins (Bem1, Bzz1, and Sla1) the numbers correspond to the domain numbering given in Supplementary Fig. 1a. Source data are provided as a Source Data file.

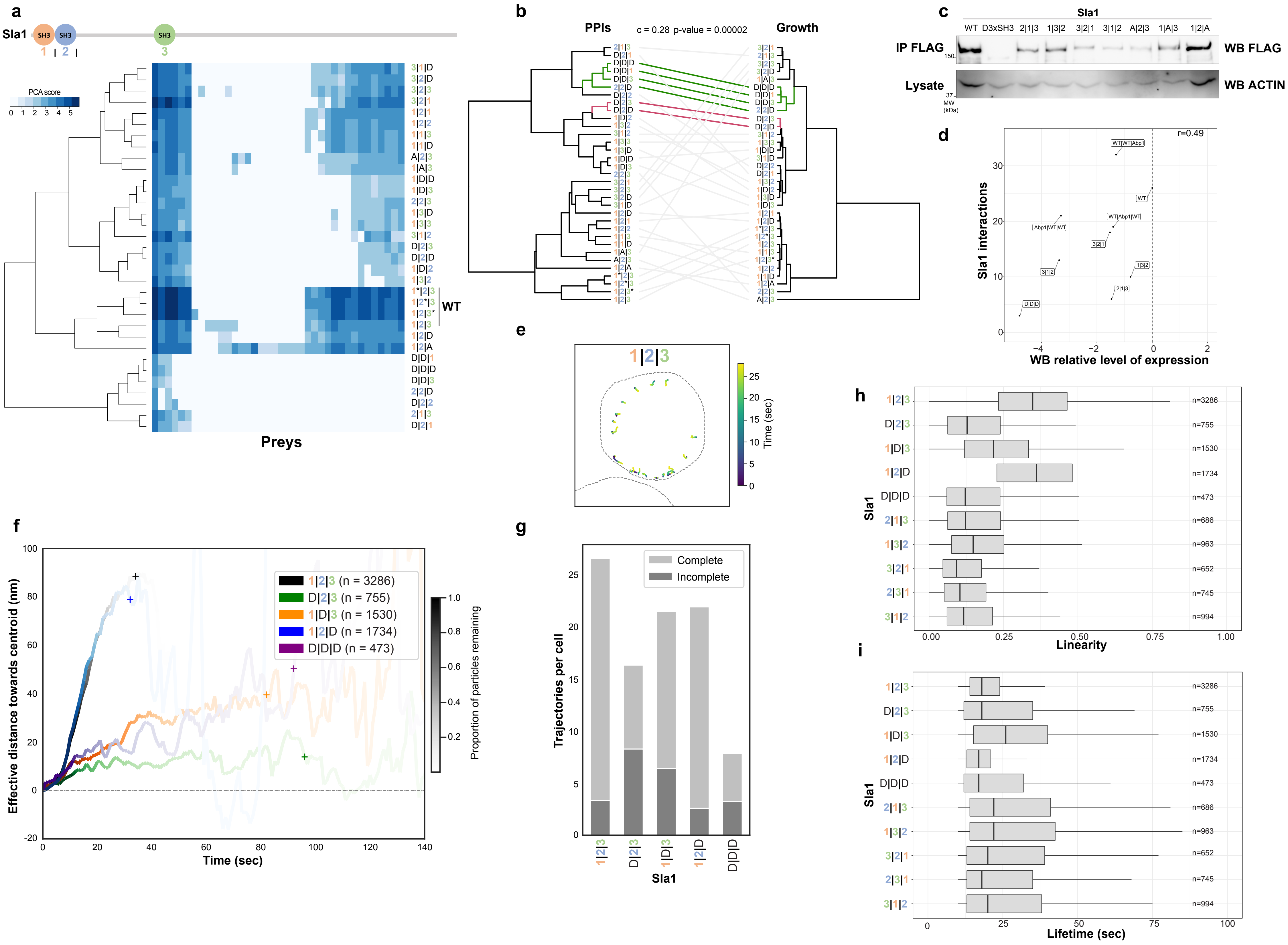

Supplementary Fig. 5| SH3 shuffling impacts Sla1 PPIs, cells growth in stress conditions and clathrin-mediated endocytosis. a Sla1 SH3-deleted or -shuffled PPIs as detected by DHFR-PCA. The color code represents PPI strength as detected by DHFR-PCA (PCA score). All PPIs were measured in quadruplicate. "A" is for Abp1 SH3 and "*" is for the reinsertion of the WT domain as control strains. A scaled cartoon of Sla1 is also illustrated. **b** Cophenetic correlation for the similarity of Sla1 SH3-shuffled PPI clusters with the growth phenotype clusters. Empirical p-value obtained from data permutation is p = 0.00002. **c** Protein expression levels of the main Sla1 SH3-shuffled proteins as compared to WT Sla1. The letter "A" stands for Abp1 SH3 inserted into Sla1. The WB validation of the expression level of Sla1 baits was performed in one replicate. **d** The relative level of expression of Sla1 SH3-shuffled baits quantified by WB (log2 of Sla1 SH3-shuffled/Sla1 WT ratio, WB relative level of expression of 0 for WT Sla1) compared to their number of PPIs as identified by DHFR-PCA (Pearson's r = 0.49, p-value = 0.18, two-sided). The level of expression of Sla1 proteins was normalized on the level of the loading control Actin. **e** Representative fluorescence microscopy timeframe analysis of a yeast cell expressing WT Sla1-GFP. The calculated trajectories for every Sla1-GFP foci detected are shown. Cell membrane is delimited with a dashed line and each Sla1-GFP particle color represents its position in time. **f** Sla1-GFP particles average effective distance travelled towards the cell center through time. Sla1 SH3-deleted strains are shown. The proportion of events that are not completed yet is represented by the color transparency of the curves (+ represent the time point when 95% of foci have disassembled). **g** Complete or incomplete Sla1-GFP endocytosis events are shown (average per cell) for Sla1 SH3-deleted strains (n = 3286 Sla1-GFP endocytosis foci for Sla1 1|2|3, n = 473 Sla1-GFP endocytosis foci for Sla1 D|D|D, n = 755 Sla1-GFP endocytosis foci for Sla1 D|2|3, n = 1530 Sla1-GFP endocytosis foci for Sla1 1|D|3, n = 1734 Sla1-GFP endocytosis foci for Sla1 1|2|D). **h** Linearity of Sla1-GFP particle trajectories for each SH3 deletion or shuffling. **i** Sla1-GFP particle lifetime (in seconds) are shown for the same strains as in panel f. For the boxplot, the median is represented as a bold center line and hinges are for the 25th and 75th percentiles (first and third quartiles). Whiskers extend from the hinges to maximum 1.5 times the Q3-Q1 interquartile range. Source data are provided as a Source Data file. See also Supplementary Data 1 and Supplementary Data 2.

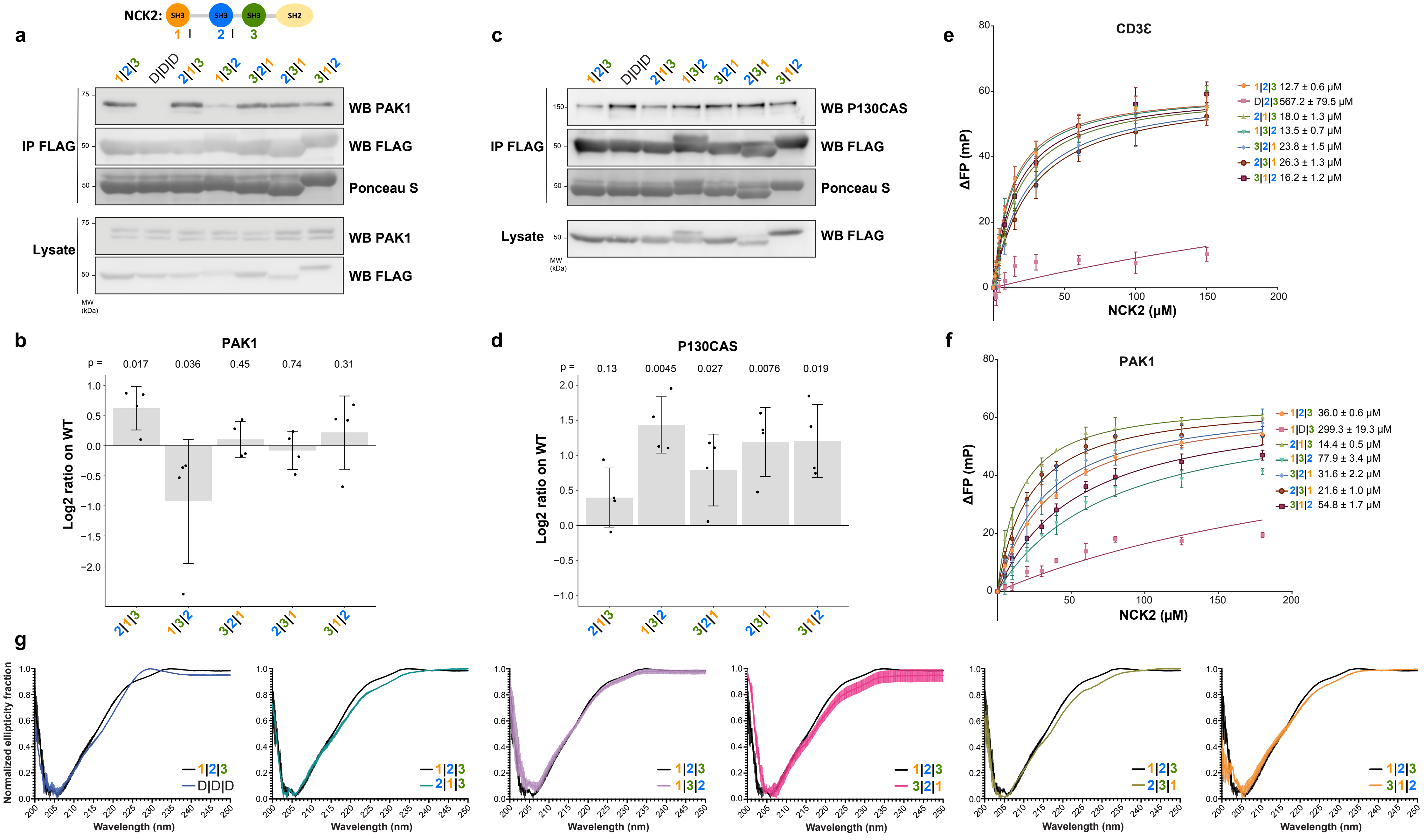

Supplementary Fig. 6| NCK2 SH3 shuffling affects its interactions with PAK1 and P130CAS/BCAR1 in cells but only slightly alters the availability of the SH3 binding pockets in vitro. a Western blot analysis of NCK2 SH3-shuffled proteins interaction with PAK1. Each bait is N-terminally fused to a 3XFLAG tag. Loading corresponds to Ponceau staining of the nitrocellulose membrane. Experiment was performed in four replicates in HEK293T cells. **b** Western blot quantification of PAK1 co-immunoprecipitation with NCK2. Average log2 ratio relative to the WT protein is shown (NCK2mut/NCK2WT, WT NCK2 log2 ratio = 0). Ratios from replicates were compared to WT NCK2 via pairwise one-way ANOVA statistical test (p-values: 2|1|3 = 0.017, 1|3|2 = 0.036, 3|2|1 = 0.45, 2|3|1 = 0.74 and 3|1|2 = 0.31, n = 4 independent biological replicates). Error bar represents the mean plus and minus one standard deviation. **c** Western blot analysis of NCK2 SH3-shuffled proteins association with SH2 target P130CAS/BCAR1. The experiment was performed in four replicates in HEK293T cells stimulated with the tyrosine phosphatase inhibitor pervanadate. **d** Quantification of the Western blot signals of P130CAS co-immunoprecipitation with NCK2 as in panel b. The four ratios of the replicates were compared to WT NCK2 via pairwise one-way ANOVA statistical test (p-values: 2|1|3 = 0.13, 1|3|2 = 0.0045, 3|2|1 = 0.027, 2|3|1 = 0.0076 and 3|1|2 = 0.019, n = 4 independent biological replicates). Error bar represents the mean plus and minus one standard deviation. **e-f** Fluorescence polarization in vitro curves for NCK2 binding to CD3Ɛ or PAK1. Each binding assay was executed in triplicate. Error bars represent plus and minus one standard deviation of the average of the ΔFP for each point. Dissociation constants error values represent the SE of the value derived from the binding curve. **g** Far-UV CD spectra of NCK2 recombinant proteins. WT NCK2 spectrum (black) is overlaid on SH3-shuffled proteins or NCK2 D|D|D triple SH3-inactive negative control (W38K/W148K/W234K) spectra. Bold traces and vertical dashed lines represent the mean and SEM calculated values for each triplicate measurement, respectively. WT NCK2, NCK2 D|D|D and NCK2 SH3-shuffled proteins similarly exhibit a mix between random coil and folded secondary structure elements. Source data are provided as a Source Data file. See also Supplementary Data 3.

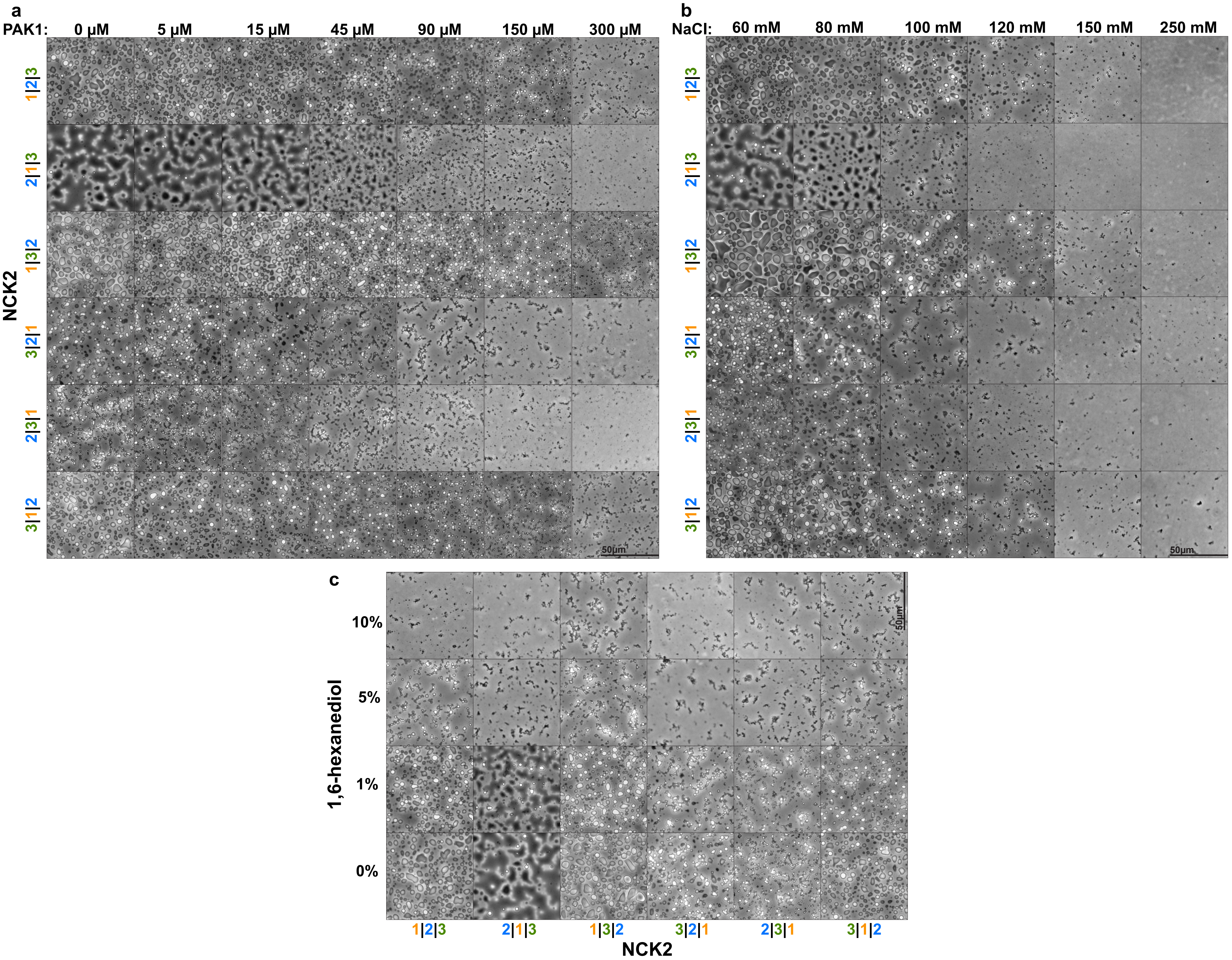

Supplementary Fig. 7| NCK2 SH3-shuffled proteins ability to promote phase transition is perturbed by PAK1, NaCl and 1,6-hexanediol. a-c An increasing amount of PAK1 (**a** PAK1 peptide), salt (**b** NaCl) or 1,6-hexanediol (**c**) was added to the different NCK2 SH3-shuffled protein samples and incubated for 24 hours before observing phase transition using phase contrast microscopy. The experiment was performed in triplicates.

Supplementary Table 1.
Primers used in this study

DNA sequence	Description
AATGATACGGCGACCACCGAGATCTACACTCTTTCCCTACAC GACGCTCTTCCGATCTNNNNNCAAGTGTTCTAACTTACGGAG TCGCTCTACG	P01_PE1.0 plate primer FORWARD with index 1 (CAAGTGTTC), use to add illumina P5 and barcode for HTS sequencing
AATGATACGGCGACCACCGAGATCTACACTCTTTCCCTACAC GACGCTCTTCCGATCTNNNNNAGGACATTCTAACTTACGGAG TCGCTCTACG	P02_PE1.0 plate primer FORWARD with index 2 (AGGACATTC), use to add illumina P5 and barcode for HTS sequencing
AATGATACGGCGACCACCGAGATCTACACTCTTTCCCTACAC GACGCTCTTCCGATCTNNNNNCACTAATGGTAACTTACGGAG TCGCTCTACG	P03_PE1.0 plate primer FORWARD with index 3 (CACTAATGG), use to add illumina P5 and barcode for HTS sequencing
CAAGCAGAAGACGGCATACGAGATCGGTCTCGGCATTCCTGC TGAACCGCTCTTCCGATCTNNNNNCAAGTGTTCGGATGGGAT TCTTTAGGTCCTG	P01_PE2.0 plate primer REVERSE with index 1 (CAAGTGTTC), use to add illumina P7 and barcode for HTS sequencing
CAAGCAGAAGACGGCATACGAGATCGGTCTCGGCATTCCTGC TGAACCGCTCTTCCGATCTNNNNNAGGACATTCGGATGGGAT TCTTTAGGTCCTG	P02_PE2.0 plate primer REVERSE with index 2 (AGGACATTC), use to add illumina P7 and barcode for HTS sequencing
CAAGCAGAAGACGGCATACGAGATCGGTCTCGGCATTCCTGC TGAACCGCTCTTCCGATCTNNNNNCACTAATGGGGATGGGAT TCTTTAGGTCCTG	P03_PE2.0 plate primer REVERSE with index 3 (CACTAATGG), use to add illumina P7 and barcode for HTS sequencing
TCTGCAGCTCCTCCTCCGCCTCCAAGACGAGCAACTCCAGAG AAAAAGCCAAAGGAAAAT	Oligo-F to amplify stuffer for ABP1/YCR088W SH3 (535-592)
AGCCAACGGGACTCTTCACCAAAAAACAGACATAATTCTAAAG ATATTACTTCTCCAGAG	Oligo-F to amplify stuffer for BEM1/YBR200W SH3-1 (223-393)
ATCATTTGTAACCTTCCGACTTGAACTATTGGATCCATTCACA GAGGAAGGTTTAGTTCT	Oligo-R to amplify stuffer for BEM1/YBR200W SH3-1 (223-393)
GTGAATGGATCCAATAGTTCAAGTCGGAAGGTTACAAATGATT CATTAAACATGGGATCG	Oligo-F to amplify stuffer for BEM1/YBR200W SH3-2 (472-648)
TAGGTTAACTGACTTGATGTCTTCTATTACGTCGTTACCTGTT GCGTACCCCGTGGCAAT	Oligo-R to amplify stuffer for BEM1/YBR200W SH3-2 (472-648)
TTACCGTATTACATTTAAATTTGCATAATGGCCACTAACTTAAC ATCTTTGAAGCCACCA	Oligo-F to amplify stuffer for CYK3/YDL117W SH3 (34-207)
AAAACTTTCTACTATTTTTGAAGGTTGCCTACCATTTTCAGTGC TCGAATTTAATCTCTC	Oligo-R to amplify stuffer for CYK3/YDL117W SH3 (34-207)
AGGGATAGAGGTATTACTGTTACCTTGCCTATTGTCACCAGTG AAGGTTTTCCAGTCATT	Oligo-F to amplify stuffer for HOF1/YMR032W SH3 (1804-1998)
GCAGTTCAATGGTCGAACCATAGACCCACCGCCTCCTGATCC GCCACCGCCAAGACCTTG	Oligo-R to amplify stuffer for HOF1/YMR032W SH3 (1804-1998)
AGACAGGCAAATATCCCTCCACCACCTCCTCCACCCCCACCC TCTTCAAAGCCAAAAGAA	Oligo-F to amplify stuffer for MYO5/YMR109W SH3 (3262-3438)
ATTCAGAACGGGTTTAGGAACATCCCTGTTTTGAGGAGGAGT AGGGATATTATTATTTCC	Oligo-R to amplify stuffer for MYO5/YMR109W SH3 (3262-3438)
CATAGCCAAGGCGACAGAGTGTGTTATATACAAAAGAGCTAG AGTATGACTGTGTTTCTG	Oligo-F to amplify stuffer for SLA1/YBL007C SH3-1 (16-207)
TTCTTCATCAGCATTTTGCACCTGTTCATAATCATAAATGGCTC TTACCTTCTTCAAAAC	Oligo-R to amplify stuffer for SLA1/YBL007C SH3-1 (16-207)
GATAGCGAAGAACCGGTGGGTCTAGTGCCCTCCACTTACATT GAAGAAGCTCCTGTTTTG	Oligo-F to amplify stuffer for SLA1/YBL007C SH3-2 (214-393)
AGGAGTAGCTGCAGGAGCTTCAGCGGCAGCGGGAGCCTGTT CCTGCTTGGAAGTGGACCC	Oligo-R to amplify stuffer for SLA1/YBL007C SH3-2 (214-393)
TATAAAGGTGCTTCCCGTGATCCTGGGTTGAGAGAAGTCGAA ATGGCTTCCAAATCCAAA	Oligo-F to amplify stuffer for SLA1/YBL007C SH3-3 (1066-1242)

GAAGTTTTTCTTGATAGACTTGATGATACCGCTTGCTGTAGATTCAGTATGTTTTTTGTC	Oligo-R to amplify stuffer for SLA1/YBL007C SH3-3 (1066-1242)
GAGCTACTAGAAGTAAACACAGTCTTACCCAAACTAAGGATGAGTGAACCCGAAGTGCCC	Oligo-F to amplify stuffer for BBC1/YJL020C SH3 (8-68)
TGTACGTTGCTCAGTGGAACCGGTATTCGGAGATGATTCGGCTTCCTTTCCAACTTCAGA	Oligo-R to amplify stuffer for BBC1/YJL020C SH3 (8-68)
AATAAAAAATAGAAGATGAGTCTCGAAGGAAATACCCTAGGCAAAGGGGCCAAATCTTTT	Oligo-F to amplify stuffer for BOI1/YBL085W SH3 (16-76)
AACACCAGAATTTCCACTCTCTTGGGTTGGTGATTTGTGCAGGTTCTCTGGTTTTTCTAT	Oligo-R to amplify stuffer for BOI1/YBL085W SH3 (16-76)
ACTTTGTCACCGGATTTTGACAGCAAAGGAAGTGCAACCGGGCGTGACGGTGGCAATTTC	Oligo-F to amplify stuffer for BOI2/YER114C SH3 (46-106)
TAATGGACTATATATCCTTTTGGTAGACTTGGCTCTCATCAATGTCGGAGCCTTCTCCAC	Oligo-R to amplify stuffer for BOI2/YER114C SH3 (46-106)
GATTTGGAGGAGGAAAACGACGATTACCAACCCTTATCGCCGCCAAGAGAACTGGACCCT	Oligo-F to amplify stuffer for BUD14/YAR014C SH3 (262-319)
AGCAACCGATTGAGAAGACATGTTTTCATTTTTCCAGCAATTCAATCGAGCTAGTCTTTC	Oligo-R to amplify stuffer for BUD14/YAR014C SH3 (262-319)
TCTATACGCACCACTAGTACCAACAATACAAAAAAGACCACACAAAATTCCAGTGATGAC	Oligo-F to amplify stuffer for BZZ1/YHR114W SH3-1 (496-554)
CCTTCTTGGTGGTGGTACTTCTGGTGCAGGGCCTCTATCATTTGCTTTAACTGTAGCCGC	Oligo-R to amplify stuffer for BZZ1/YHR114W SH3-1 (496-554)
GCAAATGATAGAGGCCCTGCACCAGAAGTACCACCACCAAGAAGGAGTACACTACCTGTT	Oligo-F to amplify stuffer for BZZ1/YHR114W SH3-2 (580-633)
TCCTCAGCTTCTCTTTCTTCTTCCCCCTCGACAAGTGAGTTGACCAGCATTCGTCCAATT	Oligo-F to amplify stuffer for CDC25/YLR310C SH3 (61-127)
TGAGCTCTTACTGGAACTATATTTTTTCATCGGATGACTGTGCTTTCTGAGATGACTGTC	Oligo-R to amplify stuffer for CDC25/YLR310C SH3 (61-127)
AAACCATTGCCGCTTACTCCAAACTCCAAATATAATGGGGAGGCTAGCGTCCAATTAGGG	Oligo-F to amplify stuffer for FUS1/YCL027W SH3 (439-512)
ACTGCAACTGCAAACATACCAATACCACCACCCCCTCCCCCCATGGGCCAACCAAAGGAT	Oligo-F to amplify stuffer for MYO3/YKL129C SH3 (1123-1181)
CTGATTTGTAACGTCATTCACTGCCCCTGTCGCTGCAACAGGCACCGTATTTCTTGTGTC	Oligo-R to amplify stuffer for MYO3/YKL129C SH3 (1123-1181)
GGAGAAGACACATACAATAAAAGACAAAGTATAACACTACCAGACGACTACATAGTTAAC	Oligo-F to amplify stuffer for NBP2/YDR162C SH3 (113-170)
CAAATGTGTCAAATAGAAAGGTCGAGCCTTATTCTCCACCTCGTTCTCACCATCTTCCGG	Oligo-R to amplify stuffer for NBP2/YDR162C SH3 (113-170)
TCTGGAACCATACGAGCATCGCAAGGAAATGGTAGCGAGCCTATTGATCCTTCGAAGTTA	Oligo-F to amplify stuffer for PEX13/YLR191W SH3 (309-371)
AATATTGATGCACTAGTGTGTACGCGTTTCATCATCAACATGCTCAATTTTCTTCCGTCT	Oligo-R to amplify stuffer for PEX13/YLR191W SH3 (309-371)
CCTCTAACATCACCTGTTGCGGGCACACCAGCCGCGGCCGTAGCGGCAGCTCCTGGCGTT	Oligo-F to amplify stuffer for RVS167/YDR388W SH3 (424-481)
GAAACTACGATGGGAGACACTTTGGGTCTGTATAGCGATATCGGTGATGATAATTTCATT	Oligo-F to amplify stuffer for SHO1/YER118C SH3 (303-360)
CGAACCATAGACCCACCGCCTCCTGATCCGCCACCGCCACGATGCATTTCTTCTGGACC	Oligo-R to amplify stuffer for SHO1/YER118C SH3 (303-360)
AGCAAGTTACCTGAGAAATGGGATGGAAACCAAAGATCGCCCCAAAATGCAGATACAGAA	Oligo-F to amplify stuffer for LSB1/YGR136W SH3 (56-111)
GGGACGAGAAACAGTCGAGCTTGAAGCAGCTTCCGCAGATTTAGGTGAGGCTGATCTCGT	Oligo-R to amplify stuffer for LSB1/YGR136W SH3 (56-111)
TCAACACCGCAAACTAGTCAGGGCAGATTCACTGCTCCAACATCCCCATCCACGTCTTCT	Oligo-F to amplify stuffer for LSB3/YFR024C-A SH3 (403-459)

CAGCAGCATCAGCAGCAGAATCAAGCTCCTGCTCACAAAATA CCGGCACAAACCGTTGTG	Oligo-F to amplify stuffer for HSE1/YHL002W SH3 (220-275)
TTGAGAGAATACTATGGCTTCCTTATTTTTTTCCTTCTCAATTT CTTCTTTCGATGGCTC	Oligo-R to amplify stuffer for HSE1/YHL002W SH3 (220-275)
AGCTTGCCGGCAAAATGGATCCTGCCAATGCACCCCGCAAC GCCAGTCCAGCTTCCTTG	Oligo-F to amplify stuffer for PIN3/YPR154W SH3 (57-112)
TAATTCTTGAGCTTTATACTGTGGAGGTGGTGGAAGATTGGAT GGACCGTTAGACCCAGA	Oligo-R to amplify stuffer for PIN3/YPR154W SH3 (57-112)
CGATGATTTATCACATAAAATGTCGAAGACAGGGTTAGGTAAT GAATCCACTGCTACAAA	Oligo-F to amplify stuffer for YSC84/YHR016C SH3 (409-468)
GGCGACGATGCAGTTCAATGGTCGAACCATAGACCCACCGCC TCCTGATCCGCCACCGCC	Oligo-R to amplify stuffer for ABP1/YCR088W (535-592), LSB3/YFR024C-A (403-459), BZZ1/YHR114W second SH3 (580-633), FUS1/YCL027W (439-512), YSC84/YHR016C (409-468) and RVS167/YDR388W (424-481) SH3 domains
AAAAAGCCAAAGGAAAATGGCGGAAGTTCTGGAGGTGGTGGT GGCGGTGGCGGATCAGGA	Stuffer for ABP1/YCR088W SH3 (535-592)
GATATTACTTCTCCAGAGGGTGGCTCAGGAGGAGGTGGTGG AAGAACTAAACCTTCCTCT	Second stuffer for BEM1/YBR200W SH3-1 (223-393)
TCATTAAACATGGGATCGGGTGGCTCAGGAGGAGGTGGTGG AATTGCCACGGGGTACGCA	Second stuffer for BEM1/YBR200W SH3-2 (472-648)
AGTATGACTGTGTTTCTGGGTGGCTCAGGAGGAGGTGGTGG AGTTTTGAAGAAGGTAAGA	Second stuffer for SLA1/YBL007C SH3-1 (16-207)
GAAGAAGCTCCTGTTTTGGGTGGCTCAGGAGGAGGTGGTGG AGGGTCCACTTCCAAGCAG	Second stuffer for SLA1/YBL007C SH3-2 (214-393)
ATGGCTTCCAAATCCAAAGGTGGCTCAGGAGGAGGTGGTGG AGACAAAAAACATACTGAA	Second stuffer for SLA1/YBL007C SH3-3 (1066-1242)
GATATTACTTCTCCAGAGGGCGGAAGTTCTGGAGGTGGTGGT AGAACTAAACCTTCCTCT	Stuffer for BEM1/YBR200W SH3-1 (223-393)
TCATTAAACATGGGATCGGGCGGAAGTTCTGGAGGTGGTGGT ATTGCCACGGGGTACGCA	Stuffer for BEM1/YBR200W SH3-2 (472-648)
ACATCTTTGAAGCCACCAGGCGGAAGTTCTGGAGGTGGTGGT GAGAGATTAAATTCGAGC	Stuffer for CYK3/YDL117W SH3 (34-207)
TCTTCAAAGCCAAAAGAAGGCGGAAGTTCTGGAGGTGGTGGT GGAAATAATAATATCCCT	Stuffer for MYO5/YMR109W SH3 (3262-3438)
AGTATGACTGTGTTTCTGGGCGGAAGTTCTGGAGGTGGTGGT GTTTTGAAGAAGGTAAGA	Stuffer for SLA1/YBL007C SH3-1 (16-207)
GAAGAAGCTCCTGTTTTGGGCGGAAGTTCTGGAGGTGGTGG TGGGTCCACTTCCAAGCAG	Stuffer for SLA1/YBL007C SH3-2 (214-393)
ATGGCTTCCAAATCCAAAGGCGGAAGTTCTGGAGGTGGTGGT GACAAAAAACATACTGAA	Stuffer for SLA1/YBL007C SH3-3 (1066-1242)
AGTATGACTGTGTTTCTGGGTGGCTCAGGAGGAGGTGGTGG AGGGTCCACTTCCAAGCAG	Second stuffer for SLA1/YBL007C SH3-1 and SH3-2 (6-131)
AGTATGACTGTGTTTCTGGGCGGAAGTTCTGGAGGTGGTGGT GGGTCCACTTCCAAGCAG	Stuffer for SLA1/YBL007C SH3-1 and SH3-2 (6-131)
AGTGAACCCGAAGTGCCCGGCGGAAGTTCTGGAGGTGGTGG TTCTGAAGTTGGAAAGGAA	Stuffer for BBC1/YJL020C SH3 (8-68)
AAAGGGGCCAAATCTTTTGGCGGAAGTTCTGGAGGTGGTGGT ATAGAAAAACCAGAGAAC	Stuffer for BOI1/YBL085W SH3 (16-76)
CGTGACGGTGGCAATTTCGGCGGAAGTTCTGGAGGTGGTGG TGTGGAGAAGGCTCCGACA	Stuffer for BOI2/YER114C SH3 (46-106)
CCAAGAGAACTGGACCCTGGCGGAAGTTCTGGAGGTGGTGG TGAAAGACTAGCTCGATTG	Stuffer for BUD14/YAR014C SH3 (262-319)

CAAAATTCCAGTGATGACGGCGGAAGTTCTGGAGGTGGTGGT GCGGCTACAGTTAAAGCA	Stuffer for BZZ1/YHR114W SH3-1 (496-554)
CAAAATTCCAGTGATGACGGTGGCTCAGGAGGAGGTGGTGG AGCGGCTACAGTTAAAGCA	Second stuffer for BZZ1/YHR114W SH3-1 (496-554)
ACCAGCATTCGTCCAATTGGCGGAAGTTCTGGAGGTGGTGGT GACAGTCATCTCAGAAAG	Stuffer for CDC25/YLR310C SH3 (61-127)
ATGGGCCAACCAAAGGATGGCGGAAGTTCTGGAGGTGGTGG TGACACAAGAAATACGGTG	Stuffer for MYO3/YKL129C SH3 (1123-1181)
GACGACTACATAGTTAACGGCGGAAGTTCTGGAGGTGGTGGT CCGGAAGATGGTGAGAAC	Stuffer for NBP2/YDR162C SH3 (113-170)
ATTGATCCTTCGAAGTTAGGCGGAAGTTCTGGAGGTGGTGGT AGACGGAAGAAAATTGAG	Stuffer for PEX13/YLR191W SH3 (309-371)
CAAAATGCAGATACAGAAGGCGGAAGTTCTGGAGGTGGTGGT ACGAGATCAGCCTCACCT	Stuffer for LSB1/YGR136W SH3 (56-111)
CCGGCACAAACCGTTGTGGGCGGAAGTTCTGGAGGTGGTGG TGAGCCATCGAAAGAAGAA	Stuffer for HSE1/YHL002W SH3 (220-275)
GCCAGTCCAGCTTCCTTGGGCGGAAGTTCTGGAGGTGGTGG TTCTGGGTCTAACGGTCCA	Stuffer for PIN3/YPR154W SH3 (57-112)
TCCCCATCCACGTCTTCTGGCGGAAGTTCTGGAGGTGGTGGT GGCGGTGGCGGATCAGGA	Stuffer for LSB3/YFR024C-A SH3 (403-459)
AGGAGTACACTACCTGTTGGCGGAAGTTCTGGAGGTGGTGG TGGCGGTGGCGGATCAGGA	Stuffer for BZZ1/YHR114W SH3-2 (580-633)
GCGGCAGCTCCTGGCGTTGGCGGAAGTTCTGGAGGTGGTGG TGGCGGTGGCGGATCAGGA	Stuffer for RVS167/YDR388W SH3 (424-481)
GAAGGTTTTCCAGTCATTGGCGGAAGTTCTGGAGGTGGTGGT CAAGGTCTTGGCGGTGGC	Stuffer for HOF1/YMR032W SH3 (1804-1998)
GGTGATGATAATTTCATTGGCGGAAGTTCTGGAGGTGGTGGT GGTCCAGAAGAAATGCAT	Stuffer for SHO1/YER118C SH3 (303-360)
GCTAGCGTCCAATTAGGGGGCGGAAGTTCTGGAGGTGGTGG TGGCGGTGGCGGATCAGGA	Stuffer for FUS1/YCL027W SH3 (439-512)
GAATCCACTGCTACAAATGGCGGAAGTTCTGGAGGTGGTGGT GGCGGTGGCGGATCAGGA	Stuffer for YSC84/YHR016C SH3 (409-468)
AGTACCAGTGAGGTACCTTTCTTCATC	Oligo-F to confirm and sequence BEM1/YBR200W SH3-1
CCGACTTGAACTATTGGATCCAT	Oligo-R to confirm and sequence BEM1/YBR200W SH3-1
CCCCAAAACCTATTTTGAAGTG	Oligo-F to confirm and sequence BEM1/YBR200W SH3-2
TATTACGTCGTTACCTGTTGCG	Oligo-R to confirm and sequence BEM1/YBR200W SH3-2
CATTAAAGGTCAGTCCCGAAG	Oligo-F to confirm and sequence CYK3/YDL117W SH3
TTGCCTACCATTTTCAGTGC	Oligo-R to confirm and sequence CYK3/YDL117W SH3
CAGGAAACATCATGGATGAGAGAG	Oligo-F to confirm and sequence HOF1/YMR032W SH3
CACGGCAGCGTATATCTTTCTA	Oligo-R to confirm and sequence HOF1/YMR032W SH3
TGCTACACCCGCTGCTACA	Oligo-F to confirm and sequence MYO5/YMR109W SH3
CCCTGTTTTGAGGAGGAGTAGG	Oligo-R to confirm and sequence MYO5/YMR109W SH3
GCAATCCTAAAAGAAGGGATAGTG	Oligo-F to confirm and sequence SLA1/YBL007C SH3-1

TCAGCATTTTGCACCTGTTCA	Oligo-R to confirm and sequence SLA1/YBL007C SH3-1
AGACATTGACGATTGGTGGA	Oligo-F to confirm and sequence SLA1/YBL007C SH3-2
TTGTGTTGAGGAGGTGGTAA	Oligo-R to confirm and sequence SLA1/YBL007C SH3-2
CCACCACATGTGAAGAGATCA	Oligo-F to confirm and sequence SLA1/YBL007C SH3-3
ACCGCTTGCTGTAGATTCAG	Oligo-R to confirm and sequence SLA1/YBL007C SH3-3
AAGAAGAAGCTGAGGAAGCC	Oligo-F to confirm and sequence SH3 domain YCR088W ABP1
AATATAATAGCATGACGCTGACG	Oligo-R to confirm and sequence SH3 domain YCR088W ABP1
AAACATCAATCGCGTCCCAA	Oligo-F to confirm and sequence SH3 domain YJL020C BBC1
GTATTCGGAGATGATTCGGCTT	Oligo-R to confirm and sequence SH3 domain YJL020C BBC1
CAACAAAGTTCTAACTCGAGGTGAC	Oligo-F to confirm and sequence SH3 domain YBL085W BOI1
TCTCTTGGGTTGGTGATTTGTG	Oligo-R to confirm and sequence SH3 domain YBL085W BOI1
TCAACACAACTGTCAGCAGAGAT	Oligo-F to confirm and sequence SH3 domain YER114C BOI2
GGTAGACTTGGCTCTCATCAATGT	Oligo-R to confirm and sequence SH3 domain YER114C BOI2
CTCTGATGACGATGACTTTGACAG	Oligo-F to confirm and sequence SH3 domain YAR014C BUD14
GGAATCATCTTTCGAATCGGAG	Oligo-R to confirm and sequence SH3 domain YAR014C BUD14
CAACGCTGGTGAAGATTCAGAT	Oligo-F to confirm and sequence SH3 domain YHR114W BZZ1 SH3-1
CAGGGCCTCTATCATTTGCTT	Oligo-R to confirm and sequence SH3 domain YHR114W BZZ1 SH3-1
TATATTCGCATATCTAGCGCGG	Oligo-F to confirm and sequence SH3 domain YHR114W BZZ1 SH3-2
GCGGCCAGGGAAAATATTTA	Oligo-R to confirm and sequence SH3 domain YHR114W BZZ1 SH3-2
ATGCTTCACAAACTCCATCG	Oligo-F to confirm and sequence SH3 domain YLR310C CDC25
TTCATCGGATGACTGTGCTT	Oligo-R to confirm and sequence SH3 domain YLR310C CDC25
CACCCCATCAAAACCACTAAA	Oligo-F to confirm and sequence SH3 domain YCL027W FUS1
GGTATAGATTAAATGCGAACGTC	Oligo-R to confirm and sequence SH3 domain YCL027W FUS1
AAAGAAAGTGCCGAAGTCCT	Oligo-F to confirm and sequence SH3 domain YHL002W HSE1
CCCGTTTTAGAAGCTGCATT	Oligo-R to confirm and sequence SH3 domain YHL002W HSE1
TCAAACGTCATATCAGGCGA	Oligo-F to confirm and sequence SH3 domain YGR136W LSB1
AAGCAGCTTCCGCAGATTTA	Oligo-R to confirm and sequence SH3 domain YGR136W LSB1

AAACTAGTCAGGGCAGAT	Oligo-F to confirm and sequence SH3 domain YFR024C-A LSB3
ACGTTTCTCGTATTCTTT	Oligo-R to confirm and sequence SH3 domain YFR024C-A LSB3
GTATCGATGCCACCTTCAAAAG	Oligo-F to confirm and sequence SH3 domain YKL129C MYO3
TAACGTCATTCACTGCCCCT	Oligo-R to confirm and sequence SH3 domain YKL129C MYO3
TGATGGCAGTATTTGGTTTTCC	Oligo-F to confirm and sequence SH3 domain YLR191W PEX13
ATGCACTAGTGTGTACGCGTTT	Oligo-R to confirm and sequence SH3 domain YLR191W PEX13
GGTCAAATGTCATTTCAAATGACG	Oligo-F to confirm and sequence SH3 domain YPR154W PIN3
TTTATACTGTGGAGGTGGTGGAA	Oligo-R to confirm and sequence SH3 domain YPR154W PIN3
ACAATCACCACAGCAGCAACA	Oligo-F to confirm and sequence SH3 domain YDR388W RVS167
GGTAATGAATACAGAGGGATGCAG	Oligo-R to confirm and sequence SH3 domain YDR388W RVS167
CGAAGGAAACCAATGATAATAGC	Oligo-F to confirm and sequence SH3 domain YER118C SHO1
CTTTGACTCGAGAATCCATGCT	Oligo-R to confirm and sequence SH3 domain YER118C SHO1
ATGAGCAATGTCGATGAT	Oligo-F to confirm and sequence SH3 domain YHR016C YSC84
TATATATAGGAACGAGAC	Oligo-R to confirm and sequence SH3 domain YHR016C YSC84
TGTTCTCAATGAGGCAGTAT	Oligo-F to confirm and sequence SH3 domain YDR162C NBP2_V2
CGCTCTGTGTAATCAAATGT	Oligo-R to confirm and sequence SH3 domain YDR162C NBP2_V2
cgggtggcgaatgggactttAGTCAGACATTGACGATTGGgttttagagctagaaatagc	Mutagenesis for insertion of SLA1/YBL007C first SH3 domain gRNA in pCAS F
gctatttctagctctaaaacCCAATCGTCAATGTCTGACTaaagtcccattcgccacccg	Mutagenesis for insertion of SLA1/YBL007C first SH3 domain gRNA in pCAS R
cgggtggcgaatgggactttTCTGGTTCGACGTAATTGCCgttttagagctagaaatagc	Mutagenesis for insertion of SLA1/YBL007C second SH3 domain gRNA in pCAS F
gctatttctagctctaaaacGGCAATTACGTCGAACCAGAaaagtcccattcgccacccg	Mutagenesis for insertion of SLA1/YBL007C second SH3 domain gRNA in pCAS R
cgggtggcgaatgggactttATGATAAAAAATCTAAGGACgttttagagctagaaatagc	Mutagenesis for insertion of SLA1/YBL007C third SH3 domain gRNA in pCAS F
gctatttctagctctaaaacGTCCTTAGATTTTTTATCATaaagtcccattcgccacccg	Mutagenesis for insertion of SLA1/YBL007C third SH3 domain gRNA in pCAS R
cgggtggcgaatgggactttGGTGGCTCAGGAGGAGGTGGgttttagagctagaaatagc	Mutagenesis for insertion of Second stuffer gRNA in pCAS for SH3 project forward
gctatttctagctctaaaacCCACCTCCTCCTGAGCCACCaaagtcccattcgccacccg	Mutagenesis for insertion of Second stuffer gRNA in pCAS for SH3 project reverse
cgggtggcgaatgggactttGGCGGAAGTTCTGGAGGTGGgttttagagctagaaatagc	Mutagenesis for insertion of Stuffer gRNA in pCAS for SH3 project forward
gctatttctagctctaaaacCCACCTCCAGAACTTCCGCCaaagtcccattcgccacccg	Mutagenesis for insertion of Stuffer gRNA in pCAS for SH3 project reverse

cgggtggcgaatgggactttGTCGACGATGACTGGTGGCTgttttagagctagaaatagc	Mutagenesis for insertion of ABP1/YCR088W SH3 domain gRNA in pCAS F
gctatttctagctctaaaacAGCCACCAGTCATCGTCGACaaagtcccattcgccacccg	Mutagenesis for insertion of ABP1/YCR088W SH3 domain gRNA in pCAS R
cgggtggcgaatgggactttGAAGACGCCGAATGGTACTTgttttagagctagaaatagc	Mutagenesis for insertion of BBC1/YJL020C SH3 domain gRNA in pCAS F
gctatttctagctctaaaacAAGTACCATTCGGCGTCTTCaaagtcccattcgccacccg	Mutagenesis for insertion of BBC1/YJL020C SH3 domain gRNA in pCAS R
cgggtggcgaatgggactttTTATGTATCTGGAGATGAGAgttttagagctagaaatagc	Mutagenesis for insertion of BEM1/YBR200W first SH3 domain gRNA in pCAS F
gctatttctagctctaaaacTCTCATCTCCAGATACATAAaaagtcccattcgccacccg	Mutagenesis for insertion of BEM1/YBR200W first SH3 domain gRNA in pCAS R
cgggtggcgaatgggactttGCGCCCACCATAACTGTGAAgttttagagctagaaatagc	Mutagenesis for insertion of BEM1/YBR200W second SH3 domain gRNA in pCAS F
gctatttctagctctaaaacTTCACAGTTATGGTGGGCGCaaagtcccattcgccacccg	Mutagenesis for insertion of BEM1/YBR200W second SH3 domain gRNA in pCAS R
cgggtggcgaatgggactttATGGGGAGTACAATGACGGCgttttagagctagaaatagc	Mutagenesis for insertion of BOI1/YBL085W SH3 domain gRNA in pCAS F
gctatttctagctctaaaacGCCGTCATTGTACTCCCCATaaagtcccattcgccacccg	Mutagenesis for insertion of BOI1/YBL085W SH3 domain gRNA in pCAS R
cgggtggcgaatgggactttGATGAACTAGATATGAAGCCgttttagagctagaaatagc	Mutagenesis for insertion of BOI2/YER114C SH3 domain gRNA in pCAS F
gctatttctagctctaaaacGGCTTCATATCTAGTTCATCaaagtcccattcgccacccg	Mutagenesis for insertion of BOI2/YER114C SH3 domain gRNA in pCAS R
cgggtggcgaatgggactttTGAATGACCAGGATGCCTATgttttagagctagaaatagc	Mutagenesis for insertion of BUD14/YAR014C SH3 domain gRNA in pCAS F
gctatttctagctctaaaacATAGGCATCCTGGTCATTCAaaagtcccattcgccacccg	Mutagenesis for insertion of BUD14/YAR014C SH3 domain gRNA in pCAS R
cgggtggcgaatgggactttGTTGCACGCGATACGGGTTCgttttagagctagaaatagc	Mutagenesis for insertion of BZZ1/YHR114W first SH3 domain gRNA in pCAS F
gctatttctagctctaaaacGAACCCGTATCGCGTGCAACaaagtcccattcgccacccg	Mutagenesis for insertion of BZZ1/YHR114W first SH3 domain gRNA in pCAS R
cgggtggcgaatgggactttTCAGAGGCGATGACGGTAGCgttttagagctagaaatagc	Mutagenesis for insertion of BZZ1/YHR114W second SH3 domain gRNA in pCAS F
gctatttctagctctaaaacGCTACCGTCATCGCCTCTGAaaagtcccattcgccacccg	Mutagenesis for insertion of BZZ1/YHR114W second SH3 domain gRNA in pCAS R
cgggtggcgaatgggactttAACTCATCTGGGTGGTGGGAgttttagagctagaaatagc	Mutagenesis for insertion of CDC25/YLR310C SH3 domain gRNA in pCAS F
gctatttctagctctaaaacTCCCACCACCCAGATGAGTTaaagtcccattcgccacccg	Mutagenesis for insertion of CDC25/YLR310C SH3 domain gRNA in pCAS R

cgggtggcgaatgggactttATGGAAGTCACAAGGATTGCgttttagagctagaaatagc	Mutagenesis for insertion of CYK3/YDL117W SH3 domain gRNA in pCAS F
gctatttctagctctaaaacGCAATCCTTGTGACTTCCATaaagtcccattcgccacccg	Mutagenesis for insertion of CYK3/YDL117W SH3 domain gRNA in pCAS R
cgggtggcgaatgggactttTGGCCACTCATACCGATGGAgttttagagctagaaatagc	Mutagenesis for insertion of FUS1/YCL027W SH3 domain gRNA in pCAS F
gctatttctagctctaaaacTCCATCGGTATGAGTGGCCAaaagtcccattcgccacccg	Mutagenesis for insertion of FUS1/YCL027W SH3 domain gRNA in pCAS R
cgggtggcgaatgggactttGGATTAGCGAATTTCCATAAgttttagagctagaaatagc	Mutagenesis for insertion of HOF1/YMR032W SH3 domain gRNA in pCAS F
gctatttctagctctaaaacTTATGGAAATTCGCTAATCCaaagtcccattcgccacccg	Mutagenesis for insertion of HOF1/YMR032W SH3 domain gRNA in pCAS R
cgggtggcgaatgggactttATAAGCAGAGATGAACCTAGgttttagagctagaaatagc	Mutagenesis for insertion of MYO3/YKL129C SH3 domain gRNA in pCAS F
gctatttctagctctaaaacCTAGGTTCATCTCTGCTTATaaagtcccattcgccacccg	Mutagenesis for insertion of MYO3/YKL129C SH3 domain gRNA in pCAS R
cgggtggcgaatgggactttTTGGTCTCTCGGGAAACTTCgttttagagctagaaatagc	Mutagenesis for insertion of MYO5/YMR109W SH3 domain gRNA in pCAS F
gctatttctagctctaaaacGAAGTTTCCCGAGAGACCAAaaagtcccattcgccacccg	Mutagenesis for insertion of MYO5/YMR109W SH3 domain gRNA in pCAS R
cgggtggcgaatgggactttAATGAATTGAGATTGGCTGAgttttagagctagaaatagc	Mutagenesis for insertion of NBP2/YDR162C SH3 domain gRNA in pCAS F
gctatttctagctctaaaacTCAGCCAATCTCAATTCATTaaagtcccattcgccacccg	Mutagenesis for insertion of NBP2/YDR162C SH3 domain gRNA in pCAS R
cgggtggcgaatgggactttCTCTTGGGAGGGATTCTGACgttttagagctagaaatagc	Mutagenesis for insertion of PEX13/YLR191W SH3 domain gRNA in pCAS F
gctatttctagctctaaaacGTCAGAATCCCTCCCAAGAGaaagtcccattcgccacccg	Mutagenesis for insertion of PEX13/YLR191W SH3 domain gRNA in pCAS R
cgggtggcgaatgggactttGTACTCCGGACGTGAACGAAgttttagagctagaaatagc	Mutagenesis for insertion of RVS167/YDR388W SH3 domain gRNA in pCAS F
gctatttctagctctaaaacTTCGTTCACGTCCGGAGTACaaagtcccattcgccacccg	Mutagenesis for insertion of RVS167/YDR388W SH3 domain gRNA in pCAS R
cgggtggcgaatgggactttCTACAAGTCTCTGACATTGAgttttagagctagaaatagc	Mutagenesis for insertion of SHO1/YER118C SH3 domain gRNA in pCAS F
gctatttctagctctaaaacTCAATGTCAGAGACTTGTAGaaagtcccattcgccacccg	Mutagenesis for insertion of SHO1/YER118C SH3 domain gRNA in pCAS R

cgggtggcgaatgggactttGCAGGTTTCACATAATTTGCgttttagagctagaaatagc	Mutagenesis for insertion of LSB1/YGR136W SH3 domain gRNA in pCAS F
gctatttctagctctaaaacGCAAATTATGTGAAACCTGCaaagtcccattcgccacccg	Mutagenesis for insertion of LSB1/YGR136W SH3 domain gRNA in pCAS R
cgggtggcgaatgggactttCGGAGATTTACCATTCAGAAgttttagagctagaaatagc	Mutagenesis for insertion of LSB3/YFR024C SH3 domain gRNA in pCAS F
gctatttctagctctaaaacTTCTGAATGGTAAATCTCCGaaagtcccattcgccacccg	Mutagenesis for insertion of LSB3/YFR024C SH3 domain gRNA in pCAS R
cgggtggcgaatgggactttGGGCGATGTTATTACAGTGCgttttagagctagaaatagc	Mutagenesis for insertion of HSE1/YHL002W SH3 domain gRNA in pCAS F
gctatttctagctctaaaacGCACTGTAATAACATCGCCCaaagtcccattcgccacccg	Mutagenesis for insertion of HSE1/YHL002W SH3 domain gRNA in pCAS R
cgggtggcgaatgggactttTGGTACAAGGGTAGCTGTAAgttttagagctagaaatagc	Mutagenesis for insertion of PIN3/YPR154W SH3 domain gRNA in pCAS F
gctatttctagctctaaaacTTACAGCTACCCTTGTACCAaaagtcccattcgccacccg	Mutagenesis for insertion of PIN3/YPR154W SH3 domain gRNA in pCAS R
cgggtggcgaatgggactttTGGTGGACAGGCAGGACTAAgttttagagctagaaatagc	Mutagenesis for insertion of LSB4/YHR016C SH3 domain gRNA in pCAS F
gctatttctagctctaaaacTTAGTCCTGCCTGTCCACCAaaagtcccattcgccacccg	Mutagenesis for insertion of LSB4/YHR016C SH3 domain gRNA in pCAS R
cgggtggcgaatgggactttCCTATTGGTGGCTGGTTAAGgttttagagctagaaatagc	Mutagenesis for insertion of BUD14/YAR014C SH3 domain gRNA3 in pCAS F
gctatttctagctctaaaacCTTAACCAGCCACCAATAGGaaagtcccattcgccacccg	Mutagenesis for insertion of BUD14/YAR014C SH3 domain gRNA3 in pCAS R
cgggtggcgaatgggactttTTGTAGAGAGCAACAGCCGTgttttagagctagaaatagc	Mutagenesis for insertion of LSB4(YSC84)/YHR016C SH3 domain gRNA3 in pCAS F
gctatttctagctctaaaacACGGCTGTTGCTCTCTACAAaaagtcccattcgccacccg	Mutagenesis for insertion of LSB4(YSC84)/YHR016C SH3 domain gRNA3 in pCAS R
cgggtggcgaatgggactttGCTAAGCCAATTGGTCGACTgttttagagctagaaatagc	Mutagenesis for insertion of BEM1/YBR200W second SH3 domain gRNA2 in pCAS F
gctatttctagctctaaaacAGTCGACCAATTGGCTTAGCaaagtcccattcgccacccg	Mutagenesis for insertion of BEM1/YBR200W second SH3 domain gRNA2 in pCAS R
cgggtggcgaatgggactttGAGGCTGATCTCGTGAAAGCgttttagagctagaaatagc	Mutagenesis for insertion of LSB1/YGR136W SH3 domain gRNA2 in pCAS F
gctatttctagctctaaaacGCTTTCACGAGATCAGCCTCaaagtcccattcgccacccg	Mutagenesis for insertion of LSB1/YGR136W SH3 domain gRNA2 in pCAS R
CGGGTGGCGAATGGGACTTTAGACGAAATAAGAATCTCGCGTTTTAGAGCTAGAAATAGC	Mutagenesis for insertion of FUS1/YCL027W SH3 domain gRNA2 in pCAS F_
GCTATTTCTAGCTCTAAAACGCGAGATTCTTATTTCGTCTAAAGTCCCATTCGCCACCCG	Mutagenesis for insertion of FUS1/YCL027W SH3 domain gRNA2 in pCAS R_

CATAATCCAAGCCAAACTGAAAATTTCCGTTCACGATATTGGC GGTGGCGGATCAGGAGG	Oligo-F to amplify DHFR and TAG BEM1/YBR200W in Sacchraomyces cerevisiae
CAAGTAAAGAAGAAAAATGCTTCGTCTTCTAACACTAGATTTC GACACTGGATGGCGGCG	Oligo-R to amplify DHFR and TAG BEM1/YBR200W in Sacchraomyces cerevisiae
AAAAGGTCTCTTCCCCAGCAATTATGTGTCTTTGGGCAACGG CGGTGGCGGATCAGGAGG	Oligo-F to amplify DHFR and TAG ABP1/YCR088W in Sacchraomyces cerevisiae
ACGTAAGAATAATATAATAGCATGACGCTGACGTGTGATTTTC GACACTGGATGGCGGCG	Oligo-R to amplify DHFR and TAG ABP1/YCR088W in Sacchraomyces cerevisiae
AAAGGTTTTCAGAATTGTTGCGAGAGGATACGTAGGTTGGGG CGGTGGCGGATCAGGAGG	Oligo-F to amplify DHFR and TAG BBC1/YJL020C in Sacchraomyces cerevisiae
CGAAAGCATTCACAATTTCCCCTTGGAAGCATTGGGTAAATTC GACACTGGATGGCGGCG	Oligo-R to amplify DHFR and TAG BBC1/YJL020C in Sacchraomyces cerevisiae
TGACGAGGAGGCTAACGAAGATGAAGAGGAAGATGATTGGG GCGGTGGCGGATCAGGAGG	Oligo-F to amplify DHFR and TAG MYO5/YMR109W in Sacchraomyces cerevisiae
TTTGCTCGTATAGAGTATATACTCGCTAAATACATTTTGATTCG ACACTGGATGGCGGCG	Oligo-R to amplify DHFR and TAG MYO5/YMR109W in Sacchraomyces cerevisiae
AAAAGAAGGAATATTCCCTGCAAACTACGTTAGAGTTTCTGGC GGTGGCGGATCAGGAGG	Oligo-F to amplify DHFR and TAG YSC84/YHR016C in Sacchraomyces cerevisiae
AATTCTATATATAGGAACGAGACATATGGAGGACGATAATTTC GACACTGGATGGCGGCG	Oligo-R to amplify DHFR and TAG YSC84/YHR016C in Sacchraomyces cerevisiae
AGGTGTGTTTCCTGGGAACTACGTGCAACTCAACAAGAACGG CGGTGGCGGATCAGGAGG	Oligo-F to amplify DHFR and TAG RVS167/YDR388W in Sacchraomyces cerevisiae
AATAGAAGGTAATGAATACAGAGGGATGCAGGGGCCTCCTTT CGACACTGGATGGCGGCG	Oligo-R to amplify DHFR and TAG RVS167/YDR388W in Sacchraomyces cerevisiae
CAACATATTCAATGCTACTGCATCAAATCCGTTTGGATTCGGC GGTGGCGGATCAGGAGG	Oligo-F to amplify DHFR and TAG SLA1/YBL007C in Sacchraomyces cerevisiae
GTTTTAGTTATTATCCTATAAAATCTTAAAATACATTAATTTCGA CACTGGATGGCGGCG	Oligo-R to amplify DHFR and TAG SLA1/YBL007C in Sacchraomyces cerevisiae
CCTGGAAAGTTCAGCATTTGAATACCCTGGTGGCAGACTTGG CGGTGGCGGATCAGGAGG	Oligo-F to amplify DHFR and TAG BOI1/YBL085W in Sacchraomyces cerevisiae
GTGTTTAAGTTGGTCAAGAAGTAACTAATGATTGCAGTTCTTC GACACTGGATGGCGGCG	Oligo-R to amplify DHFR and TAG BOI1/YBL085W in Sacchraomyces cerevisiae
TTACTTCGGGGATAATTCAAAACATAAAACCGACAAAATAGGC GGTGGCGGATCAGGAGG	Oligo-F to amplify DHFR and TAG BOI2/YER114C in Sacchraomyces cerevisiae
GCATCAAATTTGAGGCGCATCTTTTTCAATAGCTTTAAAATTC GACACTGGATGGCGGCG	Oligo-R to amplify DHFR and TAG BOI2/YER114C in Sacchraomyces cerevisiae

GGATGTGTTGATGAAACAATTGGATGAAATTATTCGTAAAGGC GGTGGCGGATCAGGAGG	Oligo-F to amplify DHFR and TAG BUD14/YAR014C in Sacchraomyces cerevisiae
TTGGATGAGAAAAAGACCAGGCTTTATTGTAAGGACAATATTC GACACTGGATGGCGGCG	Oligo-R to amplify DHFR and TAG BUD14/YAR014C in Sacchraomyces cerevisiae
TGACGGATTGAAAGGTCTATTTCCTACAAGTTACTGTAAAGGC GGTGGCGGATCAGGAGG	Oligo-F to amplify DHFR and TAG BZZ1/YHR114W in Sacchraomyces cerevisiae
GCCAGGGAAAATATTTAATAGTTTCAGTTCATTCCTTCGTTTC GACACTGGATGGCGGCG	Oligo-R to amplify DHFR and TAG BZZ1/YHR114W in Sacchraomyces cerevisiae
GAAGCTAGGTAAGAAAAAACCTCCTTCTAGGTTATTTCGAGGC GGTGGCGGATCAGGAGG	Oligo-F to amplify DHFR and TAG CDC25/YLR310C in Sacchraomyces cerevisiae
AAAGCTAAGGTTCTTCTTGATTAGCAAATTGTATAAACTTTTCG ACACTGGATGGCGGCG	Oligo-R to amplify DHFR and TAG CDC25/YLR310C in Sacchraomyces cerevisiae
TGGTATTGGGTGGTCCGTTTTTGCTGAATGGTTGTGCGTAGG CGGTGGCGGATCAGGAGG	Oligo-F to amplify DHFR and TAG CYK3/YDL117W in Sacchraomyces cerevisiae
ATACAGATTATAGCGCTGTAAAAAAATTTGTGAAAAACGTTTC GACACTGGATGGCGGCG	Oligo-R to amplify DHFR and TAG CYK3/YDL117W in Sacchraomyces cerevisiae
TAGAGGCATTGTGCCTGGTGACTGTCTCCAAGAATACGACGG CGGTGGCGGATCAGGAGG	Oligo-F to amplify DHFR and TAG FUS1/YCL027W in Sacchraomyces cerevisiae
TATAGGTATAGATTAAATGCGAACGTCAATATTATTTTCATTCG ACACTGGATGGCGGCG	Oligo-R to amplify DHFR and TAG FUS1/YCL027W in Sacchraomyces cerevisiae
AATTCCCTATAATTTCATTCAGCTACTGCATCAAGGTCTTGGC GGTGGCGGATCAGGAGG	Oligo-F to amplify DHFR and TAG HOF1/YMR032W in Sacchraomyces cerevisiae
TCTTTTATCAGAAAACTAGTAAAATTGATATACATCGAGATTCG ACACTGGATGGCGGCG	Oligo-R to amplify DHFR and TAG HOF1/YMR032W in Sacchraomyces cerevisiae
CGATGATGATAATGACGATGGCGATGATGATGATGACTGGGG CGGTGGCGGATCAGGAGG	Oligo-F to amplify DHFR and TAG MYO3/YKL129C in Sacchraomyces cerevisiae
CCATTCTATAAGAGACTAAACATCTATATATTCATGTTAATTCG ACACTGGATGGCGGCG	Oligo-R to amplify DHFR and TAG MYO3/YKL129C in Sacchraomyces cerevisiae
GGTCGAAGCAGATATGAAAACAAAATTAGATATATCGGATGGC GGTGGCGGATCAGGAGG	Oligo-F to amplify DHFR and TAG NBP2/YDR162C in Sacchraomyces cerevisiae
TAGTGAGTATTTTACTGTCATAACCAATTGGAATTTGTATTTCG ACACTGGATGGCGGCG	Oligo-R to amplify DHFR and TAG NBP2/YDR162C in Sacchraomyces cerevisiae
GAAGAAAATTGAGCATGTTGATGATGAAACGCGTACACACGG CGGTGGCGGATCAGGAGG	Oligo-F to amplify DHFR and TAG PEX13/YLR191W in Sacchraomyces cerevisiae
TATATATATATGCGAATATATGTGTGCAAATATTGATGCATTCG ACACTGGATGGCGGCG	Oligo-R to amplify DHFR and TAG PEX13/YLR191W in Sacchraomyces cerevisiae

TTATGTTCAACTAATCGATGGTCCAGAAGAAATGCATCGTGGC GGTGGCGGATCAGGAGG	Oligo-F to amplify DHFR and TAG SHO1/YER118C in Sacchraomyces cerevisiae
TCCTTTGACTCGAGAATCCATGCTATAAGATTGTTAATCATTC GACACTGGATGGCGGCG	Oligo-R to amplify DHFR and TAG SHO1/YER118C in Sacchraomyces cerevisiae
TGGTTCTGCTATTGGTAGTGATATTGTCAACAGCATTTTTGGC GGTGGCGGATCAGGAGG	Oligo-F to amplify DHFR and TAG LSB1/YGR136W in Sacchraomyces cerevisiae
TAAGTTTAAATCACTGAAACAAATATTTCGTGACAGAATATTCG ACACTGGATGGCGGCG	Oligo-R to amplify DHFR and TAG LSB1/YGR136W in Sacchraomyces cerevisiae
TAGAGAAGGTATATTCCCAGCAAATTACGTTGAACTAGTTGGC GGTGGCGGATCAGGAGG	Oligo-F to amplify DHFR and TAG LSB3/YFR024C in Sacchraomyces cerevisiae
CAATAACGTTTCTCGTATTCTTTACTCTCCTTCAAATTTTTTCG ACACTGGATGGCGGCG	Oligo-R to amplify DHFR and TAG LSB3/YFR024C in Sacchraomyces cerevisiae
ATATTCAGTTGTTAGCCAACCACCACCTGGTTATGAACAAGGC GGTGGCGGATCAGGAGG	Oligo-F to amplify DHFR and TAG HSE1/YHL002W in Sacchraomyces cerevisiae
AAAATTAAAGATATGTAAAGGTGCTATATAAGTTGAAGGGTTC GACACTGGATGGCGGCG	Oligo-R to amplify DHFR and TAG HSE1/YHL002W in Sacchraomyces cerevisiae
AGGCGCTAGTATTGGGTCAGATATTGTTAATAATATCTTTGGC GGTGGCGGATCAGGAGG	Oligo-F to amplify DHFR and TAG PIN3/YPR154W in Sacchraomyces cerevisiae
TGGAGAACAAATGAATGAAGTATATAAAGAGAAAAACGACTTC GACACTGGATGGCGGCG	Oligo-R to amplify DHFR and TAG PIN3/YPR154W in Sacchraomyces cerevisiae
AGCTCCAGAGGCTGAAGGTCCAACCGTTGAAGAAGTTGATGG CGGTGGCGGATCAGGAGGC	Oligo forward to tag YAL005C/SSA1 with DHFR
GGATGTGTTGATGAAACAATTGGATGAAATTATTCGTAAAGGC GGTGGCGGATCAGGAGGC	Oligo forward to tag YAR014C/BUD14 with DHFR
CAACATATTCAATGCTACTGCATCAAATCCGTTTGGATTCGGC GGTGGCGGATCAGGAGGC	Oligo forward to tag YBL007C/SLA1 with DHFR
TTTGAAGAGACAAGGTAGAACCTTATACGGTTTCGGTGGTGG CGGTGGCGGATCAGGAGGC	Oligo forward to tag YBR009C/HHF1 with DHFR
GGATATCAAGTTGGCTAGAAGATTAAGAGGTGAAAGATCAGG CGGTGGCGGATCAGGAGGC	Oligo forward to tag YBR010W/HHT1 with DHFR
TTCAAGAAGCAGAAAAAACGAAGGTTCTTATTTAATGCATGGC GGTGGCGGATCAGGAGGC	Oligo forward to tag YBR034C/HMT1 with DHFR
TCGTTCCTCAGCTCATATGTCTAGCAACGCCATTCAACGTGG CGGTGGCGGATCAGGAGGC	Oligo forward to tag YCR002C/CDC10 with DHFR
AAGGGAGATAAAATGCTGCCATAATGGGCGCGCATTGAAAGG CGGTGGCGGATCAGGAGGC	Oligo forward to tag YCR008W/SAT4 with DHFR
ACAAATGACAAGCCTAGATATTTGTGCGCTCGGGATAAAAGG CGGTGGCGGATCAGGAGGC	Oligo forward to tag YCR009C/RVS161 with DHFR
AAAAGGTCTCTTCCCCAGCAATTATGTGTCTTTGGGCAACGG CGGTGGCGGATCAGGAGGC	Oligo forward to tag YCR088W/ABP1 with DHFR
TGGTTTGAAGAGAGTTGTCACCAAGGCCATGTCTTCTCGTGG CGGTGGCGGATCAGGAGGC	Oligo forward to tag YDL229W/SSB1 with DHFR
AATTATTACTTTTATCGCTTCGTTAATGACTTTGAACAAAGGCG GTGGCGGATCAGGAGGC	Oligo forward to tag YDR129C/SAC6 with DHFR

ATATAACGTCGGCAAACAGCTCAGTAATGAGATTATATATGGC GGTGGCGGATCAGGAGGC	Oligo forward to tag YDR243C/PRP28 with DHFR
AGGTGTGTTTCCTGGGAACTACGTGCAACTCAACAAGAACGG CGGTGGCGGATCAGGAGGC	Oligo forward to tag YDR388W/RVS167 with DHFR
TTCATATTCGATTAATCCAGATGAAATAGAACTAGATATTGGC GGTGGCGGATCAGGAGGC	Oligo forward to tag YDR416W/SYF1 with DHFR
AGTTGAGAACGTCCTGAATAAGGAAGGCGTTCTACAAAAAGG CGGTGGCGGATCAGGAGGC	Oligo forward to tag YDR507C/GIN4 with DHFR
AGAAGCTGCAGCAAATATTCTATTCAGCGATCATGCCGACGG CGGTGGCGGATCAGGAGGC	Oligo forward to tag YEL037C/RAD23 with DHFR
CTCGCTGGCGCTTAATAGAGACGATCCACCAGATATGCTAGG CGGTGGCGGATCAGGAGGC	Oligo forward to tag YER149C/PEA2 with DHFR
CGACGGCGGATTTGATGATGGATTTGACGGTAGTGACTTTGG CGGTGGCGGATCAGGAGGC	Oligo forward to tag YFL010C/WWM1 with DHFR
TTCTGTGCTGACGGCGGGTGTTAATTCAGTGGCAATTAAAGG CGGTGGCGGATCAGGAGGC	Oligo forward to tag YFR004W/RPN11 with DHFR
TGATAGTGCACTGATCTTAATGTATAAAGGATTTGGTCTGGGC GGTGGCGGATCAGGAGGC	Oligo forward to tag YFR010W/UBP6 with DHFR
CGCCGGTGGTATAAGAAAAATAAGTGGCTCCATCAAGAAAGG CGGTGGCGGATCAGGAGGC	Oligo forward to tag YFR028C/CDC14 with DHFR
TACTCTGGGAGAATATATAGAAGAGATGAACATACGCTCGGG CGGTGGCGGATCAGGAGGC	Oligo forward to tag YGL060W/YBP2 with DHFR
ATTTACTTCAGATCAAAAAAACAAAAGGACGCAGTTTTCAGGC GGTGGCGGATCAGGAGGC	Oligo forward to tag YGL070C/RPB9 with DHFR
AATTGACTACAAGAAGTTCATCGAAGATGTTTTGAGACAAGGC GGTGGCGGATCAGGAGGC	Oligo forward to tag YGL106W/MLC1 with DHFR
AGATATTTCGAAATTGAAAGGCATCTCTAAACCGTCACCAGGC GGTGGCGGATCAGGAGGC	Oligo forward to tag YGL197W/MDS3 with DHFR
AAAGGCTGCTAGGGCTGATAGGGGTGCAAACTTTAGAGATGG CGGTGGCGGATCAGGAGGC	Oligo forward to tag YGL207W/SPT16 with DHFR
GAAAAATTTTGAGAAACCTTGCCTAATTCAAGATGGAAAAGGC GGTGGCGGATCAGGAGGC	Oligo forward to tag YGR052W/FMP48 with DHFR
GAAATATATACTATTCTTTTTGTTTCAGCTTAGTATTATGGGCG GTGGCGGATCAGGAGGC	Oligo forward to tag YGR168C/PEX35 with DHFR
AGTGGAAGAAGCGAGAGAAGAATTTGAAAATATAGCTTTAGG CGGTGGCGGATCAGGAGGC	Oligo forward to tag YGR229C/SMI1 with DHFR
TAGAGATTTGAAAACTGGTAATGCTGTAAAGGGTGAATTAGGC GGTGGCGGATCAGGAGGC	Oligo forward to tag YHR064C/SSZ1 with DHFR
AAAATACATCAAGAAGTCGGCAAAGAAAGGTGATAAGAATGG CGGTGGCGGATCAGGAGGC	Oligo forward to tag YHR140W/YHR140W with DHFR
CCAATATCTGAAGGAGTATGAATTTTCTCAAAAAGATTTTGGC GGTGGCGGATCAGGAGGC	Oligo forward to tag YHR185C/PFS1 with DHFR
AATGGGTAATACTGGATCAGGAGGGTATGATAATGCTTGGGG CGGTGGCGGATCAGGAGGC	Oligo forward to tag YIL021W/RPB3 with DHFR
TGCAGTAGACGTATTAAAGCTCAATGATCCTACAAGACATGGC GGTGGCGGATCAGGAGGC	Oligo forward to tag YIL033C/BCY1 with DHFR
TAGCGATGTCTACGTATTGTTTTATGAAAGGGTTTATGACGGC GGTGGCGGATCAGGAGGC	Oligo forward to tag YIL156W/UBP7 with DHFR
TTCTGCCTTACATTCAGAAGAAGCGTCTTTTCACTCCCTTGGC GGTGGCGGATCAGGAGGC	Oligo forward to tag YIR003W/AIM21 with DHFR
TTTATCTAAAAATGTACCGGCATTACATATGGGTGGTTTAGGC GGTGGCGGATCAGGAGGC	Oligo forward to tag YJL128C/PBS2 with DHFR
CTGTGGATCCTCTGGGAGACTGACCGCCTCACCAGTGTACG GCGGTGGCGGATCAGGAGGC	Oligo forward to tag YJL210W/PEX2 with DHFR

AGAAGATGAAGAAGAGGAAATAGAGCCGGAAGTGAAAGCTGG CGGTGGCGGATCAGGAGGC	Oligo forward to tag YKL117W/SBA1 with DHFR
CGATGATGATAATGACGATGGCGATGATGATGATGACTGGGG CGGTGGCGGATCAGGAGGC	Oligo forward to tag YKL129C/MYO3 with DHFR
CGAGATATTGAGAAACGCTAGTCAAATCGTCTCCTCTGTTGG CGGTGGCGGATCAGGAGGC	Oligo forward to tag YKR001C/VPS1 with DHFR
TAAAAAAGCCTTTGTGGAAAAATTGAACTCTAAAAAGGACGGC GGTGGCGGATCAGGAGGC	Oligo forward to tag YLL048C/YBT1 with DHFR
CTTGATCATAGGTATTTATTATGTGTTGAAATGGTTAAGAGGC GGTGGCGGATCAGGAGGC	Oligo forward to tag YLR078C/BOS1 with DHFR
CGGTAACCAGGCCATACAGTACGCTAATGAGTTCCAACAGGG CGGTGGCGGATCAGGAGGC	Oligo forward to tag YLR113W/HOG1 with DHFR
GCCTCCTGTTATCAAGAAAAGTAAAAAATGTGCAATTTTGGGC GGTGGCGGATCAGGAGGC	Oligo forward to tag YLR229C/CDC42 with DHFR
GAAGCTAGGTAAGAAAAAACCTCCTTCTAGGTTATTTCGAGGC GGTGGCGGATCAGGAGGC	Oligo forward to tag YLR310C/CDC25 with DHFR
CCACTCCCCCGTCCCTACAAAGAAGAAGGGATTTTTACGTGG CGGTGGCGGATCAGGAGGC	Oligo forward to tag YLR314C/CDC3 with DHFR
AATTGAGGCGTCTGAACCTTTCAAAACCTTCAAGAATACGGGC GGTGGCGGATCAGGAGGC	Oligo forward to tag YLR320W/MMS22 with DHFR
CGGTTTTGAATTGGTTCAACCAAAAGGAAGAAGAAAGCACGG CGGTGGCGGATCAGGAGGC	Oligo forward to tag YLR435W/TSR2 with DHFR
TAATAAAGGTAAACTTATTACCAAGGTTAACAATGAGGAGGGC GGTGGCGGATCAGGAGGC	Oligo forward to tag YML131W/YML131W with DHFR
GGAAGGTTTGAAGCAGAAGTCCACAATTGTTACCGTAGATGG CGGTGGCGGATCAGGAGGC	Oligo forward to tag YMR001C/CDC5 with DHFR
CCCCGGATGGTTTGAAGAAGTCCGCGATCATAAAAGACATGG CGGTGGCGGATCAGGAGGC	Oligo forward to tag YMR216C/SKY1 with DHFR
ACCACTGCTCCTCGCGGGACGAAAGTTATCCTTGGATAAGGG CGGTGGCGGATCAGGAGGC	Oligo forward to tag YNL020C/ARK1 with DHFR
AAGAAAAGGATTCCAGTTTGGTCTTGGTCTGGCATTTTTAGGC GGTGGCGGATCAGGAGGC	Oligo forward to tag YNL026W/SAM50 with DHFR
TTTGAAGAGACAAGGTAGAACCTTATATGGTTTCGGTGGTGG CGGTGGCGGATCAGGAGGC	Oligo forward to tag YNL030W/HHF2 with DHFR
GGTTCTGCCCTTTGGCTGTGTGGATGATGTAAACAGGCAGGG CGGTGGCGGATCAGGAGGC	Oligo forward to tag YNL073W/MSK1 with DHFR
GGAAGACAGTATTCGCAGAATTAGGGAGAAGTATTCAAACGG CGGTGGCGGATCAGGAGGC	Oligo forward to tag YNL094W/APP1 with DHFR
CGAGATAAGAAGGCATGCCTACTATAACCAGGATGATGATGG CGGTGGCGGATCAGGAGGC	Oligo forward to tag YNL243W/SLA2 with DHFR
ACTGGGATTGCAAAAGAAGAACATTAAAATTCATGGGTTTGGC GGTGGCGGATCAGGAGGC	Oligo forward to tag YNL244C/SUI1 with DHFR
CATAGGTGAGGCTAGCACAGGTAACAGGCTAAGTTTCAAAGG CGGTGGCGGATCAGGAGGC	Oligo forward to tag YNL271C/BNI1 with DHFR
CGTCGAGAAATTGATTTCGCTATCACAGTCCATGGAAAATGG CGGTGGCGGATCAGGAGGC	Oligo forward to tag YNL315C/ATP11 with DHFR
ATTAGATAACCAACCTTGGCAGTTAGCAAGATGCGGTAGGGG CGGTGGCGGATCAGGAGGC	Oligo forward to tag YOL133W/HRT1 with DHFR
TTCAGCGGCAATTCCCGTTATCCTTGCTAAAATTAAAGTCGGC GGTGGCGGATCAGGAGGC	Oligo forward to tag YOR035C/SHE4 with DHFR
TAAAACTTCACCACCAGAAGGCATGGAACCTAAACATTGGGG CGGTGGCGGATCAGGAGGC	Oligo forward to tag YOR057W/SGT1 with DHFR
CACCTTCGACATCAAAAAAGCCAACTCCGCCACGCTCGCAGG CGGTGGCGGATCAGGAGGC	Oligo forward to tag YOR091W/TMA46 with DHFR

TGCTAAATCAGCAGACCCTGATGCCATGGATACTACGGAAGG CGGTGGCGGATCAGGAGGC	Oligo forward to tag YPL235W/RVB2 with DHFR
CGACAGTCAAGACGATAGTATACTTAGTAGCGACCCCTTTGG CGGTGGCGGATCAGGAGGC	Oligo forward to tag YNL078W/NIS1 with DHFR
GGTAGACACTGTTTTAAGGAAATTTGCAGATTACAAAATTGGC GGTGGCGGATCAGGAGGC	Oligo forward to tag YDL028C/MPS1 with DHFR
AATTCCACCTGCAGGTATTCCTCCACCCCCACCCCTTCCAGG CGGTGGCGGATCAGGAGGC	Oligo forward to tag YIR006C/PAN1 with DHFR
TGTGCGATGGCACATCCAACGAATCACCTCACCGTTATCGGG CGGTGGCGGATCAGGAGGC	Oligo forward to tag YCL008C/STP22 with DHFR
AGAGAGAACGCATGCTATGCTGAACGATATTCAAAATATAGGC GGTGGCGGATCAGGAGGC	Oligo forward to tag YIL159W/BNR1 with DHFR
AAATATTGATAGTAACAATGCACAGAGTAAAATTTTCAGTGGC GGTGGCGGATCAGGAGGC	Oligo forward to tag YHR023W/MYO1 with DHFR
CAACGGACTGGAACACTCAGACCCCTTTTCAACCTTTTACGG CGGTGGCGGATCAGGAGGC	Oligo forward to tag YGR092W/DBF2 with DHFR
AAAGACATTTTCGTTATTATCAATTGCCGCACCAATTGGCTTC GACACTGGATGGCGGCGTTAG	Oligo reverse to tag YAL005C/SSA1 with DHFR
TTGGATGAGAAAAAGACCAGGCTTTATTGTAAGGACAATATTC GACACTGGATGGCGGCGTTAG	Oligo reverse to tag YAR014C/BUD14 with DHFR
GTTTTAGTTATTATCCTATAAAATCTTAAAATACATTAATTTCGA CACTGGATGGCGGCGTTAG	Oligo reverse to tag YBL007C/SLA1 with DHFR
ATATTTGCTTGTTGTTACCGTTTTCTTAGAATTAGCTAAATTCG ACACTGGATGGCGGCGTTAG	Oligo reverse to tag YBR009C/HHF1 with DHFR
TTTTGTTCGTTTTTTACTAAAACTGATGACAATCAACAAATTCG ACACTGGATGGCGGCGTTAG	Oligo reverse to tag YBR010W/HHT1 with DHFR
TGCTTTTCAAATTTTTTTCTTTCTCCAGCAAACAAAAGTCTTCG ACACTGGATGGCGGCGTTAG	Oligo reverse to tag YBR034C/HMT1 with DHFR
AATAACATAAGATATATAATCACCACCATTCTTATGAGATTTCG ACACTGGATGGCGGCGTTAG	Oligo reverse to tag YCR002C/CDC10 with DHFR
TGTTCCAAAATATCGTGATATTTGAAAGTGAAGTACTCGTTTC GACACTGGATGGCGGCGTTAG	Oligo reverse to tag YCR008W/SAT4 with DHFR
ACATAAATGACCGTAAAAAACTAAAGGCAAAAGCATTAATTTC GACACTGGATGGCGGCGTTAG	Oligo reverse to tag YCR009C/RVS161 with DHFR
ACGTAAGAATAATATAATAGCATGACGCTGACGTGTGATTTTC GACACTGGATGGCGGCGTTAG	Oligo reverse to tag YCR088W/ABP1 with DHFR
CAATATAAGTAATATTCATATATATGTGATGAATGCAGTCTTCG ACACTGGATGGCGGCGTTAG	Oligo reverse to tag YDL229W/SSB1 with DHFR
AAGCTGAGTAGAAAACAGGTTACGAAAGTTGTTTGTTGGCTTC GACACTGGATGGCGGCGTTAG	Oligo reverse to tag YDR129C/SAC6 with DHFR
TCTTGGGTCCATCAAAAAAAAGAAAGAAAATAATATAACTTTC GACACTGGATGGCGGCGTTAG	Oligo reverse to tag YDR243C/PRP28 with DHFR
AATAGAAGGTAATGAATACAGAGGGATGCAGGGGCCTCCTTT CGACACTGGATGGCGGCGTTAG	Oligo reverse to tag YDR388W/RVS167 with DHFR
TATGGTTTCGAAAATGATGCATGATTTTACATAGCTTATATTCG ACACTGGATGGCGGCGTTAG	Oligo reverse to tag YDR416W/SYF1 with DHFR
CAAAACGAAGGAGACAAAACATGATTGCATTACATTAGCATTC GACACTGGATGGCGGCGTTAG	Oligo reverse to tag YDR507C/GIN4 with DHFR
AATAAGTGAAGATACTTCAAGCCATAACATTACTACAATCTTC GACACTGGATGGCGGCGTTAG	Oligo reverse to tag YEL037C/RAD23 with DHFR
TCTATATTTATATATCAATGTTTTATAATAAGATGTTTATTTCGA CACTGGATGGCGGCGTTAG	Oligo reverse to tag YER149C/PEA2 with DHFR
ATATCATTTTATCGTCCTTGTCCAAAAAGTCTTGATAACATTCG ACACTGGATGGCGGCGTTAG	Oligo reverse to tag YFL010C/WWM1 with DHFR

TTTCTAGTTATTTAATGCATAATGACTTTATAAAATTTGTTTCG ACACTGGATGGCGGCGTTAG	Oligo reverse to tag YFR004W/RPN11 with DHFR
TCCGAAAAAACTATTAATTTTGAAAAAATGAAAAATGGACTTCG ACACTGGATGGCGGCGTTAG	Oligo reverse to tag YFR010W/UBP6 with DHFR
TTTTATTATATGATATATATATATATAAAAATGAAATAAATTCGA CACTGGATGGCGGCGTTAG	Oligo reverse to tag YFR028C/CDC14 with DHFR
TCGGGAACAAAAGAGAAGAGCAACTAATTTACTATGTAGTTTC GACACTGGATGGCGGCGTTAG	Oligo reverse to tag YGL060W/YBP2 with DHFR
TCTCTCCCTCTGTCATTAATTTTGAAAGTTCGTTGAGCACTTC GACACTGGATGGCGGCGTTAG	Oligo reverse to tag YGL070C/RPB9 with DHFR
TTATATAGTAATAAATGTCTAAATTTGCAGTTCCGCACTCTTCG ACACTGGATGGCGGCGTTAG	Oligo reverse to tag YGL106W/MLC1 with DHFR
AACTTTATATGTGCGAGTAACTATCCTGGGACGTAGCGGTTTC GACACTGGATGGCGGCGTTAG	Oligo reverse to tag YGL197W/MDS3 with DHFR
TCAAGGTCTTGCTGGTGAAACCCAGTAAGTGTTATAAAGTTTC GACACTGGATGGCGGCGTTAG	Oligo reverse to tag YGL207W/SPT16 with DHFR
GGAAAATGAGGCGAAACTTTCAAAAGGTCTTTTTTACTTCTTC GACACTGGATGGCGGCGTTAG	Oligo reverse to tag YGR052W/FMP48 with DHFR
ATTTACAAACTTGTAAACTCTTCTCTTCTAAAAAAACACATTCG ACACTGGATGGCGGCGTTAG	Oligo reverse to tag YGR168C/YGR168C with DHFR
CCAAATTTCTTTTTGGTTGTAGAATGTTAATTGTGATATTTTCG ACACTGGATGGCGGCGTTAG	Oligo reverse to tag YGR229C/SMI1 with DHFR
TTTTCTATATACGTATACATACCGTTTTTCTTAGAGCGCTTTCG ACACTGGATGGCGGCGTTAG	Oligo reverse to tag YHR064C/SSZ1 with DHFR
ACATACAAAGAGCTTAAATAGTAGTATACTAGTAGTAATGTTC GACACTGGATGGCGGCGTTAG	Oligo reverse to tag YHR140W/YHR140W with DHFR
CACAAAATTACATTTAAATATTAATGGCTTTCTTCAAAATTTCG ACACTGGATGGCGGCGTTAG	Oligo reverse to tag YHR185C/PFS1 with DHFR
GTTCACTTGTTTTTTTTCCTCTATTACGCCCACTTGAGAATTCG ACACTGGATGGCGGCGTTAG	Oligo reverse to tag YIL021W/RPB3 with DHFR
AGGAAATTCATGTGGATTTAAGATCGCTTCCCCTTTTTACTTC GACACTGGATGGCGGCGTTAG	Oligo reverse to tag YIL033C/BCY1 with DHFR
ATACATATTTATGGTGCAAAAAAAAAAATGCAAAATTTTGTTCG ACACTGGATGGCGGCGTTAG	Oligo reverse to tag YIL156W/UBP7 with DHFR
CATACTATAGAGTTTATTCATACATATTATTTATAAGATTTTCG ACACTGGATGGCGGCGTTAG	Oligo reverse to tag YIR003W/YIR003W with DHFR
TATATTCACGTGCCTGTTTGCTTTTATTTGGATATTAACGTTCG ACACTGGATGGCGGCGTTAG	Oligo reverse to tag YJL128C/PBS2 with DHFR
ATACACATATATAGAGATACAAGCGAGGGAACGGGGCCCTTT CGACACTGGATGGCGGCGTTAG	Oligo reverse to tag YJL210W/PEX2 with DHFR
GTTACTCATTCTAGCACTCCAGGTTGATTTGCTCCTCCTTTTC GACACTGGATGGCGGCGTTAG	Oligo reverse to tag YKL117W/SBA1 with DHFR
CCATTCTATAAGAGACTAAACATCTATATATTCATGTTAATTCG ACACTGGATGGCGGCGTTAG	Oligo reverse to tag YKL129C/MYO3 with DHFR
CAAAACCAAGCTTGAGTCGACCGGTATAGATGAGGAAAACTT CGACACTGGATGGCGGCGTTAG	Oligo reverse to tag YKR001C/VPS1 with DHFR
ATATATATATATATATATATATACTTTAGCATCGAAACAGTTCG ACACTGGATGGCGGCGTTAG	Oligo reverse to tag YLL048C/YBT1 with DHFR
ATATGGTATGTTTATTTATATATAGAACTCACGGTTACTTTTCG ACACTGGATGGCGGCGTTAG	Oligo reverse to tag YLR078C/BOS1 with DHFR
GAAGTAAGAATGAGTGGTTAGGGACATTAAAAAAACACGTTTC GACACTGGATGGCGGCGTTAG	Oligo reverse to tag YLR113W/HOG1 with DHFR
TAGATATAGATTAAGAAAAGATGGGCATATACTAATATGATTC GACACTGGATGGCGGCGTTAG	Oligo reverse to tag YLR229C/CDC42 with DHFR

AAAGCTAAGGTTCTTCTTGATTAGCAAATTGTATAAACTTTTCG ACACTGGATGGCGGCGTTAG	Oligo reverse to tag YLR310C/CDC25 with DHFR
TAATAGTGTATGTTTGAAATTTTTATATGTCTTTATTTCGTTCG ACACTGGATGGCGGCGTTAG	Oligo reverse to tag YLR314C/CDC3 with DHFR
CGGAGAATTTTTAAATATGCTTATATTACATGTGTATAATTTCG ACACTGGATGGCGGCGTTAG	Oligo reverse to tag YLR320W/MMS22 with DHFR
GCAGCGCACTATCAATTATAATATACAGATTTTGAAGATATTC GACACTGGATGGCGGCGTTAG	Oligo reverse to tag YLR435W/TSR2 with DHFR
TAAAAATAATATAGTCATATCTTAAACTTTTTGTGTAAAATTCG ACACTGGATGGCGGCGTTAG	Oligo reverse to tag YML131W/YML131W with DHFR
GTAATTTCGTATTCGTATTTCTTTCTACTTTAATATTGGTTTCG ACACTGGATGGCGGCGTTAG	Oligo reverse to tag YMR001C/CDC5 with DHFR
AAAAGTAAAAGGCAAGGGCAAAATAAAGGTATAAAGGTAATTC GACACTGGATGGCGGCGTTAG	Oligo reverse to tag YMR216C/SKY1 with DHFR
GCCTCTTCAGAGATCGATCCGGTTCTGTTGAGCCAAATACTT CGACACTGGATGGCGGCGTTAG	Oligo reverse to tag YNL020C/ARK1 with DHFR
TAGGCGATAGCTTCACCTTGACATTAAAAGGAATGTATTCTTC GACACTGGATGGCGGCGTTAG	Oligo reverse to tag YNL026W/SAM50 with DHFR
GAAAATAATTTCAAACACCGATTGTTTAACCACCGATTGTTTC GACACTGGATGGCGGCGTTAG	Oligo reverse to tag YNL030W/HHF2 with DHFR
TCTGATTTATTTACAAAAGCATTGGCAGGCGTCGCAAAATTTC GACACTGGATGGCGGCGTTAG	Oligo reverse to tag YNL073W/MSK1 with DHFR
TTTTTAAACTCCCTCCCGATGTATATAAATAACAGTGTATTTCG ACACTGGATGGCGGCGTTAG	Oligo reverse to tag YNL094W/APP1 with DHFR
ATTAACGTTTATCTTTATATATAAAAAGTACAATTCATGATTCG ACACTGGATGGCGGCGTTAG	Oligo reverse to tag YNL243W/SLA2 with DHFR
AAGCGAGCAGCGAGTAAGCGACTCGGCGTAAGCCTTGAACTT CGACACTGGATGGCGGCGTTAG	Oligo reverse to tag YNL244C/SUI1 with DHFR
TTTTGGTATTACTGTTGTCATAATTTTTTGGTTTAATATTTTCG ACACTGGATGGCGGCGTTAG	Oligo reverse to tag YNL271C/BNI1 with DHFR
TATATATATATATATATACGTATACGGAAGTAATTCTTCGTTCG ACACTGGATGGCGGCGTTAG	Oligo reverse to tag YNL315C/ATP11 with DHFR
ACCTCGGTATGATTTAAATGTTTACGGGCAATTCATTTTTTTC GACACTGGATGGCGGCGTTAG	Oligo reverse to tag YOL133W/HRT1 with DHFR
TGAAAATTAAGAAGAGTTAAACATGGCGATTCTTTCCATATTC GACACTGGATGGCGGCGTTAG	Oligo reverse to tag YOR035C/SHE4 with DHFR
ATATCTACATATTGTAATTGTGTAGGTATATACTAATCATTTCG ACACTGGATGGCGGCGTTAG	Oligo reverse to tag YOR057W/SGT1 with DHFR
ACGGTGGCATATATACAGTTTAGTCTATATGAATTTTAGATTC GACACTGGATGGCGGCGTTAG	Oligo reverse to tag YOR091W/YOR091W with DHFR
TATATATTTGATGCAATTTCTGCCTTAAAGTACAAAATGCTTCG ACACTGGATGGCGGCGTTAG	Oligo reverse to tag YPL235W/RVB2 with DHFR
GGCTATGCAATGGCTATGCAATCCAGGGGGGGTTACCTACTT CGACACTGGATGGCGGCGTTAG	Oligo reverse to tag YNL078W/NIS1 with DHFR
TTCATAACTGGCACATGCTTTTCTTCCTTATGCGGCTCTTTTC GACACTGGATGGCGGCGTTAG	Oligo reverse to tag YDL028C/MPS1 with DHFR
ATTAGTATACATACGTATCTATAGAAAGCAAATTAAATCTTTCG ACACTGGATGGCGGCGTTAG	Oligo reverse to tag YIR006C/PAN1 with DHFR
ATATTTTTTATGGCACTTCGGCGATGCGAAAGAAAGTGAGTTC GACACTGGATGGCGGCGTTAG	Oligo reverse to tag YCL008C/STP22 with DHFR
TTATATAAGCTCCACAACTACATAAAATACTAAGTCTTCATTCG ACACTGGATGGCGGCGTTAG	Oligo reverse to tag YIL159W/BNR1 with DHFR
AAAGGATATAAAGTCTTCCAAATTTTTAAAAAAAAGTTCGTTCG ACACTGGATGGCGGCGTTAG	Oligo reverse to tag YHR023W/MYO1 with DHFR

TTATATCGCGGCGAATGCAAGACAAGAATTCATTTTTACGTTC GACACTGGATGGCGGCGTTAG	Oligo reverse to tag YGR092W/DBF2 with DHFR
AGCGAAGATGAAGATTATGATTACG	Oligo_C to confirm tag of YSC84(LSB4)/YHR016C
TTGGAATATGTCGAAGCTCTTTATC	Oligo_C to confirm tag of PIN3/YPR154W
TGCCAATCCAACATCCTACG	Oligo_C to confirm tag of HSE1/YHL002W
AAAATCAGATTCTCAAAATGACTGG	Oligo_C to confirm tag of LSB3/YFR024C-A
TGATGGTTTGGCGAATGCGC	Oligo_C to confrim tag of MYO5/YMR109W
AGCAGCCTAAGACTGATTACAAGAA	Oligo_C to confirm tag of ABP1/YCR088W
GGTTTAATGCGAAAGTTGTAGAGAA	Oligo_C to confirm tag of BBC1/YJL020C
GAATTTGAAAGAGACGAAAATCAAA	Oligo_C to confirm tag of BEM1/YBR200W
CCTTTCAACAATTGGCGAGTTT	Oligo_C to confirm tag of BUD14/YAR014C
GGGTTCTGGATGGACTAAGATAAAT	Oligo_C to confirm tag of BZZ1/YHR114W
TCCCATTAACACATAAGGGAAGTAA	Oligo_C to confirm tag of CYK3/YDL117W
TATTCAGGATTACGAGCCTAGATTG	Oligo_C to confirm tag of FUS1/YCL027W
GGCACACGCATAGACAATCCA	Oligo_C to confirm tag of HOF1/YMR032W
AGAGCGTTATATGATTTTGTTCCAG	Oligo_C to confirm tag of PEX13/YLR191W
AAACACCAAAGGCAGTACGC	Oligo_C to confirm tag of CDC25/YLR310C
TAACGAAGATGAATACGACG	Oligo_C to confirm tag of NBP2/YDR162C
AAATGGCGAATCTGGCTCCG	Oligos_C to confirm tag of YAL031C GIP4
CGTATTGTTGGTTTAGCCGG	Oligos_C to confirm tag of YDR122W KIN1
TAAACCAAGAAGACTTAGCC	Oligos_C to confirm tag of YER155C BEM2
CGTATCGTCGGCCTAGCAGG	Oligos_C to confirm tag of YLR096W KIN2
TAATCCAGCAAAACTGTTGC	Oligos_C to confirm tag of YNL333W SNZ2
AAAGGGTCCGTCGTGAAGCG	Oligos_C to confirm tag of YBR059C AKL1
ACGAGCAGGATAACAGCAGG	Oligos_C to confirm tag of YCL034W LSB5
CTTGACTTGTCAGGAGCTGG	Oligos_C to confirm tag of YCR030C SYP1
GCAACAGCTTCAGATGCAGC	Oligos_C to confirm tag of YGL181W GTS1
GGTGAATGCAGAGGGTCCGG	Oligos_C to confirm tag of YGR268C HUA1
AGTTGGTACAGATGAAGAGG	Oligos_C to confirm tag of YIR006C PAN1
CAAAACTACCACCTCTACCG	Oligos_C to confirm tag of YNL152W INN1
GAAAATCCATTTAGTGACCC	Oligos_C to confirm tag of YNL238W KEX2
GCGAACCCATCCCACCCAGC	Oligos_C to confirm tag of YBL016W FUS3
ATTTGGATGGGATGACACCC	Oligos_C to confirm tag of YBL085W BOI1
ATCCAAGCATGAATAGTGCC	Oligos_C to confirm tag of YDR085C AFR1
ATTGAATGATGACCAAGAGG	Oligos_C to confirm tag of YER124C DSE1
AATCCTTCTACTTTTGTCGG	Oligos_C to confirm tag of YER158C
CATGAGCGTGAGCGTGATCG	Oligos_C to confirm tag of YJR056C
ATGGAGCAGGTGGGCACTGG	Oligos_C to confirm tag of YML109W ZDS2
CACCAAACCAAGTTCTATGG	Oligos_C to confirm tag of YMR124W EPO1
TACTCCTTCAAGTCTCTTCC	Oligos_C to confirm tag of YNL078W NIS1
ATGGAGAGGGCCTATTCGGG	Oligos_C to confirm tag of YBR108W AIM3
TTCGATAAGAACGGTGATGG	Oligos_C to confirm tag of YBR109C CMD1
GGAATGCCGTTAGATATGGC	Oligos_C to confirm tag of YDL028C MPS1
CTTCATTCTGAGGAGGAAGG	Oligos_C to confirm tag of YDR103W STE5
GCTCGCAATAGTAGTATGCG	Oligos_C to confirm tag of YER114C BOI2
AACGAGCTTCTTTCTCATCC	Oligos_C to confirm tag of YJL095W BCK1
TTGGGGGATATGGCTGCGGG	Oligos_C to confirm tag of YJL157C FAR1
TTTAGCAGACATTGGAAGGG	Oligos_C to confirm tag of YLR166C SEC10
TAGATATAGGGCATCAACCG	Oligos_C to confirm tag of YNL298W CLA4
TAGTAGACTCTTTGTCCAGG	Oligos_C to confirm tag of YOL070C NBA1
AAGTAACCTCGAAAAAGAGC	Oligos_C to confirm tag of YPR055W SEC8
GAAGATGAAAGTTTGCAGGC	Oligos_C to confirm tag of YBR239C ERT1
CCGTGGCACCATTCCCCTCG	Oligos_C to confirm tag of YCL008C STP22

GAAGGCAGATGGTGGAAGGC	Oligos_C to confirm tag of YER118C SHO1
TACCGATGGATGGTGTCTGG	Oligos_C to confirm tag of YCL027W FUS1
GCCATTAACGTACATGAAGG	Oligos_C to confirm tag of YIL159W BNR1
GAGGCCATTAGACGGATGCG	Oligos_C to confirm tag of YKR101W SIR1
ATACAGATGACGTTTTGTCC	Oligos_C to confirm tag of YLR330W CHS5
GACCTTCCAGATACCTCTCC	Oligos_C to confirm tag of YPR008W HAA1
CAAAGTGCAGGAAATGGCCC	Oligos_C to confirm tag of YHR023W MYO1
ATTGGTGGTGCCGTTTTAGC	Oligos_C to confirm tag of YDL029W ARP2
GATCCCCACTTTCCTTATGG	Oligos_C to confirm tag of YDL117W CYK3
CCATAAGGGCGACTATCTGC	Oligos_C to confirm tag of YMR032W HOF1
CTCCAAACACGATAACAAGG	Oligos_C to confirm tag of YDL127W PCL2
CGGATGCTACTAAAGGTTCC	Oligos_C to confirm tag of YDR142C PEX7
TTGGATCTCTCTTGAATACG	Oligos_C to confirm tag of YDR244W PEX5
TGAGTGATCCTAGCTGCGCG	Oligos_C to confirm tag of YDR265W PEX10
CAAGTGCTTCCGTTCGTTCC	Oligos_C to confirm tag of YGL252C RTG2
AATGAATATGGAAGAGGACG	Oligos_C to confirm tag of YJR083C ACF4
AATATGGCTCTGCTTGATCC	Oligos_C to confirm tag of YLR144C ACF2
GATTTCAGAACTAGAAAGGC	Oligos_C to confirm tag of YMR192W GYL1
ATTGCATCCAAGTTCTATGC	Oligos_C to confirm tag of YMR287C DSS1
TGAACTGCAAGCGGAAATCG	Oligos_C to confirm tag of YPL249C GYP5
GATGATAAGACCCATAGGCC	Oligos_C to confirm tag of YER047C SAP1
GGGACACTATACTTCATACG	Oligos_C to confirm tag of YER144C UBP5
GATAGCAACGCCTCCATTCC	Oligos_C to confirm tag of YGL153W PEX14
GAACAAAAGATACGGTTCGC	Oligos_C to confirm tag of YGL190C CDC55
GTTGAACATCTGTCTCCAGC	Oligos_C to confirm tag of YGR077C PEX8
CTTGTTGGGTTCACTTTCCG	Oligos_C to confirm tag of YGR092W DBF2
GCAGTGGGGCTAGTAGTGCC	Oligos_C to confirm tag of YGR136W LSB1
GCTGAAGATAAAGAGAATGC	Oligos_C to confirm tag of YGR218W CRM1
AGATGGAAATTCCGCAAGCG	Oligos_C to confirm tag of YGR239C PEX21
AATTTGGCTACTGTTGAACC	Oligos_C to confirm tag of YKL213C DOA1
TAACGGTACAGAAAGAAACC	Oligos_C to confirm tag of YHR160C PEX18
ATTCAACCAATTCTAAGGCG	Oligos_C to confirm tag of YIL106W MOB1
TTAACAGATAGGTCCCGAGC	Oligos_C to confirm tag of YNL214W PEX17
TCTGCTCACAGGTCTGCTGC	Oligos_C to confirm tag of YOL044W PEX15
ATGAAAGCACCACATATCCC	Oligos_C to confirm tag of YMR001C CDC5
CCATTTGCTATGCTAAATCC	Oligos_C to confirm tag of YPL158C AIM44
ACGATACTCGGATGATGTCG	Oligos_C to confirm tag of YPL242C IQG1
AGTTCACTAAGGAAAAACCG	Oligos_C to confirm tag of YMR104C YPK2
CAATACGTTACTGTCACCGG	Oligos_C to confirm tag of YOR247W SRL1
GGTGCTCCAGGTGGCGCTGC	Ologo_C to confirm tag of YAL005C/SSA1
ATATGTCCGATCCGGCTTCG	Ologo_C to confirm tag of YAR014C/BUD14
TTACAGAACCAACCTACTGG	Ologo_C to confirm tag of YBL007C/SLA1
AATTCACCTCTGCGCCACCC	Ologo_C to confirm tag of YBL105C/PKC1
TTACCTACACCGAACACGCC	Ologo_C to confirm tag of YBR009C/HHF1
TGCAAGAATCTGTCGAAGCC	Ologo_C to confirm tag of YBR010W/HHT1
GGTGAATTGGTTTGCTCTCC	Ologo_C to confirm tag of YBR034C/HMT1
ACGCGGTATTGATTTGTTGG	Ologo_C to confirm tag of YBR160W/CDC28
TCATCTCCAAGACTTAATCG	Ologo_C to confirm tag of YCR002C/CDC10
CGATGTTAGATCCCGTTCCG	Ologo_C to confirm tag of YCR008W/SAT4
AATCAAGAGACGACTATGCC	Ologo_C to confirm tag of YCR009C/RVS161
TGCGAAGCGAATTGAAGTCC	Ologo_C to confirm tag of YDL031W/DBP10
AAATTCCCCTGCTCAAGAGC	Ologo_C to confirm tag of YDL064W/UBC9
CGATGCTTTGGCTGCTTTGC	Ologo_C to confirm tag of YDL229W/SSB1
AGTTGGGTGCTTTGATTTGG	Ologo_C to confirm tag of YDR129C/SAC6

TGAAGCTCCAGTTTGTGTGG	Ologo_C to confirm tag of YDR172W/SUP35
ACGTCAGGAAACATGATCCC	Ologo_C to confirm tag of YDR243C/PRP28
AAGAAAAGAAAACTTGGCGG	Ologo_C to confirm tag of YDR365C/ESF1
AATTGTCCAGCGTACTCCGG	Ologo_C to confirm tag of YDR388W/RVS167
TAACATTAACTTCGTGGCGG	Ologo_C to confirm tag of YDR416W/SYF1
GAAATTGGATAGCGCATCGG	Ologo_C to confirm tag of YDR449C/UTP6
AGGTAAGATGAGCGTTGTGC	Ologo_C to confirm tag of YDR507C/GIN4
ACGATCAAGCTATTTCGCGC	Ologo_C to confirm tag of YEL037C/RAD23
TTCACCACTGGTACTTCTCG	Ologo_C to confirm tag of YER125W/RSP5
GAATTTGATAACGCTGGTGC	Ologo_C to confirm tag of YER133W/GLC7
TGAACATAGATTCAAGCTGG	Ologo_C to confirm tag of YER149C/PEA2
CACCTTGGCGAAGTTAATCC	Ologo_C to confirm tag of YFL005W/SEC4
AATTACTACGGTGATGATGC	Ologo_C to confirm tag of YFL010C/WWM1
ATTGGTGGTTCTATCTTGGC	Ologo_C to confirm tag of YFL039C/ACT1
GCAAGATCCAAAGAAGCACC	Ologo_C to confirm tag of YFR004W/RPN11
CATAAGGGACGAACTGGACG	Ologo_C to confirm tag of YFR010W/UBP6
TACTAAGACAACTATTGCCC	Ologo_C to confirm tag of YFR028C/CDC14
ATCTCCGAATCATTTCAAGG	Ologo_C to confirm tag of YGL060W/YBP2
TCCCAAATGTCACTCTCGGG	Ologo_C to confirm tag of YGL070C/RPB9
AACCGACGCTGAAGTAGACG	Ologo_C to confirm tag of YGL106W/MLC1
TGGTTCGTTGCATGGATTGC	Ologo_C to confirm tag of YGL197W/MDS3
TGAGGACTATACTGGCGACG	Ologo_C to confirm tag of YGL207W/SPT16
AAGATAGCTCGATGGACAGC	Ologo_C to confirm tag of YGR052W/FMP48
ATTCTTTGCGAAGTCCTTGG	Ologo_C to confirm tag of YGR168C/YGR168C
GGTTGAAGAAAAAGAACACG	Ologo_C to confirm tag of YGR229C/SMI1
CGACGATAACGACAACGAGC	Ologo_C to confirm tag of YHL007C/STE20
ACCAAGTTGATGGAATTGGG	Ologo_C to confirm tag of YHR064C/SSZ1
GAAAGAATCAGACTGAACGG	Ologo_C to confirm tag of YHR107C/CDC12
TGAACGAGCCGTACAAGTCG	Ologo_C to confirm tag of YHR140W/YHR140W
AACATCATGTATGGAGACGG	Ologo_C to confirm tag of YHR185C/PFS1
GCTGGGAAGTAACGAAGACG	Ologo_C to confirm tag of YIL021W/RPB3
CAAAGAGAACCAAAGTTGCC	Ologo_C to confirm tag of YIL033C/BCY1
GACGATGAGGTTGTCAAGGC	Ologo_C to confirm tag of YIL156W/UBP7
GATGGAGGACCAAATGGAGG	Ologo_C to confirm tag of YIR003W/YIR003W
ATACAGAAACCAGGATGTCC	Ologo_C to confirm tag of YJL128C/PBS2
TCGTGCGAACTACTGTTACG	Ologo_C to confirm tag of YJL210W/PEX2
GACCAAGACCGAAGAATTGC	Ologo_C to confirm tag of YJR045C/SSC1
ACGGTAGGATTGCCATTTGG	Ologo_C to confirm tag of YKL018W/SWD2
AGCGAAGCGTATCTGGTAGG	Ologo_C to confirm tag of YKL049C/CSE4
GCTGGTTCTCCAGATATGGC	Ologo_C to confirm tag of YKL117W/SBA1
CGGTTTAGCTTCTGCATTGG	Ologo_C to confirm tag of YKL129C/MYO3
CGAAAAACTTTACGGAAAGC	Ologo_C to confirm tag of YKR001C/VPS1
GGTGATGGATGCAGGTGAGG	Ologo_C to confirm tag of YLL048C/YBT1
TTTCGGAACAAACTATCACC	Ologo_C to confirm tag of YLR078C/BOS1
CACTCAGATATAGCTGGTGG	Ologo_C to confirm tag of YLR113W/HOG1
TCGGCACTAACACAACGCGG	Ologo_C to confirm tag of YLR229C/CDC42
AAACACCAAAGGCAGTACGC	Ologo_C to confirm tag of YLR310C/CDC25
CTACAGGTCTTCTAAATTGG	Ologo_C to confirm tag of YLR314C/CDC3
GATTCCTCATACTACTTTGC	Ologo_C to confirm tag of YLR320W/MMS22
GAGATGGACGAGGTTGTACC	Ologo_C to confirm tag of YLR435W/TSR2

TCCGCTACCCTTGAAGATGG	Ologo_C to confirm tag of YML131W/YML131W
ATGAAAGCACCACATATCCC	Ologo_C to confirm tag of YMR001C/CDC5
GGGTATGGAAGAGATTCGGG	Ologo_C to confirm tag of YMR216C/SKY1
AAAACAAAACCTACCCCACC	Ologo_C to confirm tag of YNL020C/ARK1
CCCAATGGCAAGATTCGAGC	Ologo_C to confirm tag of YNL026W/SAM50
TTACTTACACTGAACACGCC	Ologo_C to confirm tag of YNL030W/HHF2
TAGGTGGCTTTGGCCTTGGC	Ologo_C to confirm tag of YNL073W/MSK1
ATGGAAGAGAAGAGTAATGG	Ologo_C to confirm tag of YNL094W/APP1
TGGTGAAGAAGAAGACGCCG	Ologo_C to confirm tag of YNL189W/SRP1
CGAAATGGAACAGCAAGTGG	Ologo_C to confirm tag of YNL243W/SLA2
AAGGACTTTGCATGTAATGG	Ologo_C to confirm tag of YNL244C/SUI1
CTCTGACACACCAGTAAAGC	Ologo_C to confirm tag of YNL271C/BNI1
AGTTGCTAAACAGAGAGTGC	Ologo_C to confirm tag of YNL315C/ATP11
TAATCACGCTTTCCATTTGC	Ologo_C to confirm tag of YOL133W/HRT1
CGAGGTGTATCCGTTGCTGC	Ologo_C to confirm tag of YOR035C/SHE4
AAAGGGCTATGATGAAATCC	Ologo_C to confirm tag of YOR057W/SGT1
GAAGGCTGACCACCAAGACG	Ologo_C to confirm tag of YOR091W/YOR091W
GTGGAAGATGTCAAAAGGGC	Ologo_C to confirm tag of YPL235W/RVB2
TCTCTCTGACAACAAGTACC	Ologo_C to confirm tag of YPR093C/YPR093C
TGCGATCCAAGTGACTATCC	Ologo_C to confirm tag of YPR159W/KRE6
TACTCCTTCAAGTCTCTTCC	Ologo_C to confirm tag of YNL078W/NIS1
GGAATGCCGTTAGATATGGC	Ologo_C to confirm tag of YDL028C/MPS1
GCTGAAGATAAAGAGAATGC	Ologo_C to confirm tag of YGR218W/CRM1
AGTTGGTACAGATGAAGAGG	Ologo_C to confirm tag of YIR006C/PAN1
ATGGAGAGGGCCTATTCGGG	Ologo_C to confirm tag of YBR108W/AIM3
CCGTGGCACCATTCCCCTCG	Ologo_C to confirm tag of YCL008C/STP22
GCCATTAACGTACATGAAGG	Ologo_C to confirm tag of YIL159W/BNR1
CAAAGTGCAGGAAATGGCCC	Ologo_C to confirm tag of YHR023W/MYO1
TGAACTGCAAGCGGAAATCG	Ologo_C to confirm tag of YPL249C/GYP5
CTTGTTGGGTTCACTTTCCG	Ologo_C to confirm tag of YGR092W/DBF2
GTGTTATATACAAAAGAGCTAGAGTATGACTGTGTTTCTGCCT TGGGCCACAGCAGAATA	Oligo-F to Switch the SH3 of ABP1/YCR088W in SH3-1 of SLA1/YBL007C
CCTGTTCATAATCATAAATGGCTCTTACCTTCTTCAAAACGTT GCCCAAAGACACATAAT	Oligo-R to Switch the SH3 of ABP1/YCR088W in SH3-1 of SLA1/YBL007C
GTTTTGAAGAAGGTAAGAGCCATTTATGATTATGAACAGGCCT TGGGCCACAGCAGAATA	Oligo-F to Switch the SH3 of ABP1/YCR088W in SH3-2 of SLA1/YBL007C
CAGCGGCAGCGGGAGCCTGTTCCTGCTTGGAAGTGGACCCG TTGCCCAAAGACACATAAT	Oligo-R to Switch the SH3 of ABP1/YCR088W in SH3-2 of SLA1/YBL007C
TCCTGGGTTGAGAGAAGTCGAAATGGCTTCCAAATCCAAACC TTGGGCCACAGCAGAATA	Oligo-F to Switch the SH3 of ABP1/YCR088W in SH3-3 of SLA1/YBL007C
TGATGATACCGCTTGCTGTAGATTCAGTATGTTTTTGTCGTT GCCCAAAGACACATAAT	Oligo-R to Switch the SH3 of ABP1/YCR088W in SH3-3 of SLA1/YBL007C
GTGTTATATACAAAAGAGCTAGAGTATGACTGTGTTTCTGAAG AGAGGTATTGTTCAATA	Oligo-F to Switch the SH3-3 of SLA1/YBL007C in SH3-1 of SLA1/YBL007C

CCTGTTCATAATCATAAATGGCTCTTACCTTCTTCAAAACACG AACAGGCTCAATAAACT	Oligo-R to Switch the SH3-3 of SLA1/YBL007C in SH3-1 of SLA1/YBL007C
TCCTGGGTTGAGAGAAGTCGAAATGGCTTCCAAATCCAAAGG CATCTATAGGGCCGTCTA	Oligo-F to Switch the SH3-1 of SLA1/YBL007C in SH3-3 of SLA1/YBL007C
TGATGATACCGCTTGCTGTAGATTCAGTATGTTTTTTGTCAGG AGCTTCTTCAATGTAAG	Oligo-R to Switch the SH3-1 of SLA1/YBL007C in SH3-3 of SLA1/YBL007C
TCCTGGGTTGAGAGAAGTCGAAATGGCTTCCAAATCCAAAAA GAAGGTAAGAGCCATTTA	Oligo-F to Switch the SH3-2 of SLA1/YBL007C in SH3-3 of SLA1/YBL007C
TGATGATACCGCTTGCTGTAGATTCAGTATGTTTTTTGTCATT CTCTGGTTCGACGTAAT	Oligo-R to Switch the SH3-2 of SLA1/YBL007C in SH3-3 of SLA1/YBL007C
TCTAGTGCCCTCCACTTACATTGAAGAAGCTCCTGTTTTGAAG AGAGGTATTGTTCAATA	Oligo-F to Switch the SH3-3 of SLA1/YBL007C in SH3-2 of SLA1/YBL007C
CAGCGGCAGCGGGAGCCTGTTCCTGCTTGGAAGTGGACCCA CGAACAGGCTCAATAAACT	Oligo-R to Switch the SH3-3 of SLA1/YBL007C in SH3-2 of SLA1/YBL007C
TCTAGTGCCCTCCACTTACATTGAAGAAGCTCCTGTTTTGGGC ATCTATAGGGCCGTCTA	Oligo-F to Switch the SH3-1 of SLA1/YBL007C in SH3-2 of SLA1/YBL007C
CAGCGGCAGCGGGAGCCTGTTCCTGCTTGGAAGTGGACCCA GGAGCTTCTTCAATGTAAG	Oligo-R to Switch the SH3-1 of SLA1/YBL007C in SH3-2 of SLA1/YBL007C
GTGTTATATACAAAAGAGCTAGAGTATGACTGTGTTTCTGAAG AAGGTAAGAGCCATTTA	Oligo-F to Switch the SH3-2 of SLA1/YBL007C in SH3-1 of SLA1/YBL007C
CCTGTTCATAATCATAAATGGCTCTTACCTTCTTCAAAACATTC TCTGGTTCGACGTAAT	Oligo-R to Switch the SH3-2 of SLA1/YBL007C in SH3-1 of SLA1/YBL007C
TCCAAGACGAGCAACTCCAGAGAAAAAGCCAAAGGAAAATTT CAAGGTGGTGGCTCAGTT	Oligo-F to Switch the SH3 of BBC1/YJL020C in ABP1/YCR088W
GTCGAACCATAGACCCACCGCCTCCTGATCCGCCACCGCCTC CCTGAACAGCAACAAAAC	Oligo-R to Switch the SH3 of BBC1/YJL020C in ABP1/YCR088W
TCCAAGACGAGCAACTCCAGAGAAAAAGCCAAAGGAAAATAA AGTTATAAAAGCCAAATA	Oligo-F to Switch the first SH3 of BEM1/YBR200W in ABP1/YCR088W
GTCGAACCATAGACCCACCGCCTCCTGATCCGCCACCGCCAT CAAACACTTCAAAATAGG	Oligo-R to Switch the first SH3 of BEM1/YBR200W in ABP1/YCR088W
TCCAAGACGAGCAACTCCAGAGAAAAAGCCAAAGGAAAATTTA TACGCCATTGTTTTATA	Oligo-F to Switch the second SH3 of BEM1/YBR200W in ABP1/YCR088W
GTCGAACCATAGACCCACCGCCTCCTGATCCGCCACCGCCAT CTATGATGCTAACAAACC	Oligo-R to Switch the second SH3 of BEM1/YBR200W in ABP1/YCR088W
TCCAAGACGAGCAACTCCAGAGAAAAAGCCAAAGGAAAATCC TCTGTATATTGCGGTAAA	Oligo-F to Switch the SH3 of BOI1/YBL085W in ABP1/YCR088W
GTCGAACCATAGACCCACCGCCTCCTGATCCGCCACCGCCTG CTATTCTTTTGGTAAATA	Oligo-R to Switch the SH3 of BOI1/YBL085W in ABP1/YCR088W
TCCAAGACGAGCAACTCCAGAGAAAAAGCCAAAGGAAAATCC AATGTATATTGCCATTAA	Oligo-F to Switch the SH3 of BOI2/YER114C in ABP1/YCR088W
GTCGAACCATAGACCCACCGCCTCCTGATCCGCCACCGCCTG TTATCTTTTGAGTAAAGA	Oligo-R to Switch the SH3 of BOI2/YER114C in ABP1/YCR088W
TCCAAGACGAGCAACTCCAGAGAAAAAGCCAAAGGAAAATGA TAAACTATATGCTTTATA	Oligo-F to Switch the SH3 of Bud14/YAR014C in ABP1/YCR088W
GTCGAACCATAGACCCACCGCCTCCTGATCCGCCACCGCCTG GAAAGGTTTCTAGAATTT	Oligo-R to Switch the SH3 of Bud14/YAR014C in ABP1/YCR088W
TCCAAGACGAGCAACTCCAGAGAAAAAGCCAAAGGAAAATGG CAAGAATAAAGTGTTGTA	Oligo-F to Switch the first SH3 of BZZ1/YHR114W in ABP1/YCR088W
GTCGAACCATAGACCCACCGCCTCCTGATCCGCCACCGCCG CTAGATATGCGAATATATG	Oligo-R to Switch the first SH3 of BZZ1/YHR114W in ABP1/YCR088W
TCCAAGACGAGCAACTCCAGAGAAAAAGCCAAAGGAAAATAG GACGATGGAGGCCATATA	Oligo-F to Switch the second SH3 of BZZ1/YHR114W in ABP1/YCR088W
GTCGAACCATAGACCCACCGCCTCCTGATCCGCCACCGCCTT TACAGTAACTTGTAGGAA	Oligo-R to Switch the second SH3 of BZZ1/YHR114W in ABP1/YCR088W

TCCAAGACGAGCAACTCCAGAGAAAAAGCCAAAGGAAAATGG AATAGTAGTCGCTGCTTA	Oligo-F to Switch the SH3 of CDC25/YLR310C in ABP1/YCR088W
GTCGAACCATAGACCCACCGCCTCCTGATCCGCCACCGCCTC TTAAAGGTCTACCGAAGT	Oligo-R to Switch the SH3 of CDC25/YLR310C in ABP1/YCR088W
TCCAAGACGAGCAACTCCAGAGAAAAAGCCAAAGGAAAATTTT AAGGTGAAGGCCAGGTA	Oligo-F to Switch the SH3 of CYK3/YDL117W in ABP1/YCR088W
GTCGAACCATAGACCCACCGCCTCCTGATCCGCCACCGCCCT CTAATAGTATGACGAAGT	Oligo-R to Switch the SH3 of CYK3/YDL117W in ABP1/YCR088W
TCCAAGACGAGCAACTCCAGAGAAAAAGCCAAAGGAAAATAA GACATATACAGTTATTCA	Oligo-F to Switch the SH3 of FUS1/YCL027W in ABP1/YCR088W
GTCGAACCATAGACCCACCGCCTCCTGATCCGCCACCGCCGT CGTATTCTTGGAGACAGT	Oligo-R to Switch the SH3 of FUS1/YCL027W in ABP1/YCR088W
TCCAAGACGAGCAACTCCAGAGAAAAAGCCAAAGGAAAATGA ATATGCCAAGGCCATGTA	Oligo-F to Switch the SH3 of HOF1/YMR032W in ABP1/YCR088W
GTCGAACCATAGACCCACCGCCTCCTGATCCGCCACCGCCAT GCAGTAGCTGAATGAAAT	Oligo-R to Switch the SH3 of HOF1/YMR032W in ABP1/YCR088W
TCCAAGACGAGCAACTCCAGAGAAAAAGCCAAAGGAAAATCC GAAATTCGAAGCTGCATA	Oligo-F to Switch the SH3 of MYO3/YKL129C in ABP1/YCR088W
GTCGAACCATAGACCCACCGCCTCCTGATCCGCCACCGCCTT TATAAGGAGTCATATAAG	Oligo-R to Switch the SH3 of MYO3/YKL129C in ABP1/YCR088W
TCCAAGACGAGCAACTCCAGAGAAAAAGCCAAAGGAAAATCC CATGTTTGAAGCGGCTTA	Oligo-F to Switch the SH3 of MYO5/YMR109W in ABP1/YCR088W
GTCGAACCATAGACCCACCGCCTCCTGATCCGCCACCGCCG GAATGTGGTTTCATGTAAG	Oligo-R to Switch the SH3 of MYO5/YMR109W in ABP1/YCR088W
TCCAAGACGAGCAACTCCAGAGAAAAAGCCAAAGGAAAATCA ACGTGCTGTTGCCCTCTA	Oligo-F to Switch the SH3 of NBP2/YDR162C in ABP1/YCR088W
GTCGAACCATAGACCCACCGCCTCCTGATCCGCCACCGCCCT GTATATAAGAGACAAACT	Oligo-R to Switch the SH3 of NBP2/YDR162C in ABP1/YCR088W
TCCAAGACGAGCAACTCCAGAGAAAAAGCCAAAGGAAAATGA ATTTGCAAGAGCGTTATA	Oligo-F to Switch the SH3 of PEX13/YLR191W in ABP1/YCR088W
GTCGAACCATAGACCCACCGCCTCCTGATCCGCCACCGCCTT TTATGATCTCAATATAGT	Oligo-R to Switch the SH3 of PEX13/YLR191W in ABP1/YCR088W
TCCAAGACGAGCAACTCCAGAGAAAAAGCCAAAGGAAAATGA AACTGTTACCGCATTGTA	Oligo-F to Switch the SH3 of RVS167/YDR388W in ABP1/YCR088W
GTCGAACCATAGACCCACCGCCTCCTGATCCGCCACCGCCCT TGTTGAGTTGCACGTAGT	Oligo-R to Switch the SH3 of RVS167/YDR388W in ABP1/YCR088W
TCCAAGACGAGCAACTCCAGAGAAAAAGCCAAAGGAAAATTA CAAGGCAAAAGCACTGTA	Oligo-F to Switch the SH3 of SHO1/YER118C in ABP1/YCR088W
GTCGAACCATAGACCCACCGCCTCCTGATCCGCCACCGCCAT CGATTAGTTGAACATAAT	Oligo-R to Switch the SH3 of SHO1/YER118C in ABP1/YCR088W
TCCAAGACGAGCAACTCCAGAGAAAAAGCCAAAGGAAAATGG CATCTATAGGGCCGTCTA	Oligo-F to Switch the first SH3 of SLA1/YBL007C in ABP1/YCR088W
GTCGAACCATAGACCCACCGCCTCCTGATCCGCCACCGCCAG GAGCTTCTTCAATGTAAG	Oligo-R to Switch the first SH3 of SLA1/YBL007C in ABP1/YCR088W
TCCAAGACGAGCAACTCCAGAGAAAAAGCCAAAGGAAAATAA GAAGGTAAGAGCCATTTA	Oligo-F to Switch the second SH3 of SLA1/YBL007C in ABP1/YCR088W
GTCGAACCATAGACCCACCGCCTCCTGATCCGCCACCGCCAT TCTCTGGTTCGACGTAAT	Oligo-R to Switch the second SH3 of SLA1/YBL007C in ABP1/YCR088W
TCCAAGACGAGCAACTCCAGAGAAAAAGCCAAAGGAAAATAA GAGAGGTATTGTTCAATA	Oligo-F to Switch the third SH3 of SLA1/YBL007C in ABP1/YCR088W
GTCGAACCATAGACCCACCGCCTCCTGATCCGCCACCGCCAC GAACAGGCTCAATAAACT	Oligo-R to Switch the third SH3 of SLA1/YBL007C in ABP1/YCR088W
TCCAAGACGAGCAACTCCAGAGAAAAAGCCAAAGGAAAATGA GTACGTTGAAGCTTTATA	Oligo-F to Switch the SH3 of LSB1/YGR136W in ABP1/YCR088W

GTCGAACCATAGACCCACCGCCTCCTGATCCGCCACCGCCGAAAGCAGGTTTCACATAAT	Oligo-R to Switch the SH3 of LSB1/YGR136W in ABP1/YCR088W
TCCAAGACGAGCAACTCCAGAGAAAAAGCCAAAGGAAAATCCAAAGGCCGTGGCATTATA	Oligo-F to Switch the SH3 of LSB3/YFR024C in ABP1/YCR088W
GTCGAACCATAGACCCACCGCCTCCTGATCCGCCACCGCCAACTAGTTCAACGTAATTTG	Oligo-R to Switch the SH3 of LSB3/YFR024C in ABP1/YCR088W
TCCAAGACGAGCAACTCCAGAGAAAAAGCCAAAGGAAAATAGAAGAGTTCGGGCTCTATA	Oligo-F to Switch the SH3 of HSE1/YHL002W in ABP1/YCR088W
GTCGAACCATAGACCCACCGCCTCCTGATCCGCCACCGCCAACAATCGGTGTTACATAAT	Oligo-R to Switch the SH3 of HSE1/YHL002W in ABP1/YCR088W
TCCAAGACGAGCAACTCCAGAGAAAAAGCCAAAGGAAAATGAATATGTCGAAGCTCTTTA	Oligo-F to Switch the SH3 of PIN3/YPR154W in ABP1/YCR088W
GTCGAACCATAGACCCACCGCCTCCTGATCCGCCACCGCCGAAAGCTGGCTTGACATAGT	Oligo-R to Switch the SH3 of PIN3/YPR154W in ABP1/YCR088W
TCCAAGACGAGCAACTCCAGAGAAAAAGCCAAAGGAAAATTCGGCGACACCAACGGCTGT	Oligo-F to Switch the SH3 of YSC84/YHR016C in ABP1/YCR088W
GTCGAACCATAGACCCACCGCCTCCTGATCCGCCACCGCCAGAAACTCTAACGTAGTTTG	Oligo-R to Switch the SH3 of YSC84/YHR016C in ABP1/YCR088W
TCCAAGACGAGCAACTCCAGAGAAAAAGCCAAAGGAAAATTGTACCTATCAATATTTTAC	Oligo-F to Switch the SH3 of SDC25/YLL017W in ABP1/YCR088W
GTCGAACCATAGACCCACCGCCTCCTGATCCGCCACCGCCGACCGTGTAAAAAGAAGGGG	Oligo-R to Switch the SH3 of SDC25/YLL017W in ABP1/YCR088W
TCCAAGACGAGCAACTCCAGAGAAAAAGCCAAAGGAAAATCCTTGGGCCACAGCAGAATA	Oligo-F to put back the SH3 of APB1/YCR088W in ABP1/YCR088W
GTCGAACCATAGACCCACCGCCTCCTGATCCGCCACCGCCGTTGCCCAAAGACACATAAT	Oligo-R to put back the SH3 of APB1/YCR088W in ABP1/YCR088W
TCCAAGACGAGCAACTCCAGAGAAAAAGCCAAAGGAAAATGCCCTTGGAATTAGTGCTGT	Oligo-F to Switch the SH3 of ABP1 human ortholog HCLS1 in ABP1/YCR088W
GTCGAACCATAGACCCACCGCCTCCTGATCCGCCACCGCCCTCAAGCAACTTGACGTAGT	Oligo-R to Switch the SH3 of ABP1 human ortholog HCLS1 in ABP1/YCR088W
TCCAAGACGAGCAACTCCAGAGAAAAAGCCAAAGGAAAATGGTCAAGGTCTATGTGCTAG	Oligo-F to Switch the SH3 of ABP1 human ortholog DBNL in ABP1/YCR088W
GTCGAACCATAGACCCACCGCCTCCTGATCCGCCACCGCCCTCGATCAGTTCGACGTAAT	Oligo-R to Switch the SH3 of ABP1 human ortholog DBNL in ABP1/YCR088W
TCCAAGACGAGCAACTCCAGAGAAAAAGCCAAAGGAAAATGATTTGGGGATAACTGCGGT	Oligo-F to Switch the SH3 of ABP1 human ortholog CTTN in ABP1/YCR088W
GTCGAACCATAGACCCACCGCCTCCTGATCCGCCACCGCCCTGCCTCAACTCCACGTAGT	Oligo-R to Switch the SH3 of ABP1 human ortholog CTTN in ABP1/YCR088W
GTGTTATATACAAAAGAGCTAGAGTATGACTGTGTTTCTGGGCATCTATAGGGCCGTCTA	Oligo-F to put back the SH3-1 of SLA1/YBL007C in SLA1/YBL007C
CCTGTTCATAATCATAAATGGCTCTTACCTTCTTCAAAACAGGAGCTTCTTCAATGTAAG	Oligo-R to put back the SH3-1 of SLA1/YBL007C in SLA1/YBL007C
TCTAGTGCCCTCCACTTACATTGAAGAAGCTCCTGTTTTGAAGAAGGTAAGAGCCATTTA	Oligo-F to put back the SH3-2 of SLA1/YBL007C in SLA1/YBL007C
CAGCGGCAGCGGGAGCCTGTTCCTGCTTGGAAGTGGACCCATTCTCTGGTTCGACGTAAT	Oligo-R to put back the SH3-2 of SLA1/YBL007C in SLA1/YBL007C
TCCTGGGTTGAGAGAAGTCGAAATGGCTTCCAAATCCAAAAAGAGAGGTATTGTTCAATA	Oligo-F to put back the SH3-3 of SLA1/YBL007C in SLA1/YBL007C
TGATGATACCGCTTGCTGTAGATTCAGTATGTTTTTTGTCACGAACAGGCTCAATAAACT	Oligo-R to put back the SH3-3 of SLA1/YBL007C in SLA1/YBL007C
GCGGCTCATAGGCATAGACGGCCCTATAGATGCCCAAAACATTCTCTGGTTCGACGTAAT	Oligo-R Fusion PCR First fragment for SH3-2--SH3-1 of SLA1/YBL007C
CTTCATTCCAGGCAATTACGTCGAACCAGAGAATGTTTTGGGCATCTATAGGGCCGTCTA	Oligo-F Fusion PCR Second fragment for SH3-2--SH3-1 of SLA1/YBL007C

GCGGCTCATAGGCATAGACGGCCCTATAGATGCCCAAAACACGAACAGGCTCAATAAACT	Oligo-R Fusion PCR First fragment for SH3-3--SH3-1 of SLA1/YBL007C
TCTCGTTCCTGCACAGTTTATTGAGCCTGTTCGTGTTTTGGGCATCTATAGGGCCGTCTA	Oligo-F Fusion PCR Second fragment for SH3-3--SH3-1 of SLA1/YBL007C
CAGCCATGAAGTCATATTGAACAATACCTCTCTTCAAAACATTCTCTGGTTCGACGTAAT	Oligo-R Fusion PCR First fragment for SH3-2--SH3-3 of SLA1/YBL007C
CTTCATTCCAGGCAATTACGTCGAACCAGAGAATGTTTTGAAGAGAGGTATTGTTCAATA	Oligo-F Fusion PCR Second fragment for SH3-2--SH3-3 of SLA1/YBL007C
GCGAATTCCAGCTGCCATCAAGAAGCTCT	Oligo-F EcoR1 to clone in the pUC19 vector the SH3 domains at the locus of the SH3 of ABP1/YCR088W-DHFR1,2-FLAG
GCGGATCCCCCCATATTTTGGGACACGGC	Oligo-R BamH1 to clone in the pUC19 vector the SH3 domains at the locus of the SH3 of ABP1/YCR088W-DHFR1,2-FLAG
CCAAGGCGACAGAGTGTGTTATATACAAAAGAGCTAGAGTcagctgaagcttcgtacgc	Oligo-F for the insertion of NAT casette at SLA1/YBL007C locus
GTTTTAGTTATTATCCTATAAAATCTTAAAATACATTAATcataggccactagtggatc	Oligo-R for the insertion of NAT casette at SLA1/YBL007C locus
AAGCACTGTGTGAAAAGAAATTGTCAAGAAAGCCATATAAcagctgaagcttcgtacgc	Oligo-F for the insertion of NAT casette at BEM1/YBR200W locus
CAAGTAAAGAAGAAAAATGCTTCGTCTTCTAACACTAGATcataggccactagtggatc	Oligo-R for the insertion of NAT casette at BEM1/YBR200W locus
CTATCCTTAAACGCCAACTACTACATTACTTGCAATAAAAcagctgaagcttcgtacgc	Oligo-F for the insertion of NAT casette at BZZ1/YHR114W locus
GCCAGGGAAAATATTTAATAGTTTCAGTTCATTCCTTCGTcataggccactagtggatc	Oligo-R for the insertion of NAT casette at BZZ1/YHR114W locus
ATAACCGCACGTATATACACGCACACACCTATCAATCACAcagctgaagcttcgtacgc	Oligo-F for the insertion of NAT casette at ABP1/YCR088W locus
ACGTAAGAATAATATAATAGCATGACGCTGACGTGTGATTcataggccactagtggatc	Oligo-R for the insertion of NAT casette at ABP1/YCR088W locus
GCCAAAGGAAAATNNNTGGGCCACAGCAG	DMS_ABP1_P01_for; For Deep Mutational Scanning (DMS) of ABP1's SH3 domain at residue P01. Forward primer.
GCCAAAGGAAAATCCTNNNGCCACAGCAGAATATG	DMS_ABP1_W02_for; For Deep Mutational Scanning (DMS) of ABP1's SH3 domain at residue W02. Forward primer.
CCAAAGGAAAATCCTTGGNNNACAGCAGAATATGATTAC	DMS_ABP1_A03_for; For Deep Mutational Scanning (DMS) of ABP1's SH3 domain at residue A03. Forward primer.
GGAAAATCCTTGGGCCNNNGCAGAATATGATTACG	DMS_ABP1_T04_for; For Deep Mutational Scanning (DMS) of ABP1's SH3 domain at residue T04. Forward primer.
GAAAATCCTTGGGCCACANNNGAATATGATTACGATGC	DMS_ABP1_A05_for; For Deep Mutational Scanning (DMS) of ABP1's SH3 domain at residue A05. Forward primer.
CCTTGGGCCACAGCANNNTATGATTACGATGCTGC	DMS_ABP1_E06_for; For Deep Mutational Scanning (DMS) of ABP1's SH3 domain at residue E06. Forward primer.
CCTTGGGCCACAGCAGAANNNGATTACGATGCTGCAG	DMS_ABP1_Y07_for; For Deep Mutational Scanning (DMS) of ABP1's SH3 domain at residue Y07. Forward primer.
GGGCCACAGCAGAATATNNNTACGATGCTGCAGAAG	DMS_ABP1_D08_for; For Deep Mutational Scanning (DMS) of ABP1's SH3 domain at residue D08. Forward primer.

GCCACAGCAGAATATGATNNNGATGCTGCAGAAGATAAC	DMS_ABP1_Y09_for; For Deep Mutational Scanning (DMS) of ABP1's SH3 domain at residue Y09. Forward primer.
CAGCAGAATATGATTACNNNGCTGCAGAAGATAACG	DMS_ABP1_D10_for; For Deep Mutational Scanning (DMS) of ABP1's SH3 domain at residue D10. Forward primer.
GCAGAATATGATTACGATNNNGCAGAAGATAACGAACTG	DMS_ABP1_A11_for; For Deep Mutational Scanning (DMS) of ABP1's SH3 domain at residue A11. Forward primer.
GAATATGATTACGATGCTNNNGAAGATAACGAACTGACC	DMS_ABP1_A12_for; For Deep Mutational Scanning (DMS) of ABP1's SH3 domain at residue A12. Forward primer.
GATTACGATGCTGCANNNGATAACGAACTGACC	DMS_ABP1_E13_for; For Deep Mutational Scanning (DMS) of ABP1's SH3 domain at residue E13. Forward primer.
GATTACGATGCTGCAGAANNNAACGAACTGACCTTTGTG	DMS_ABP1_D14_for; For Deep Mutational Scanning (DMS) of ABP1's SH3 domain at residue D14. Forward primer.
CGATGCTGCAGAAGATNNNGAACTGACCTTTGTGG	DMS_ABP1_N15_for; For Deep Mutational Scanning (DMS) of ABP1's SH3 domain at residue N15. Forward primer.
GATGCTGCAGAAGATAACNNNCTGACCTTTGTGG	DMS_ABP1_E16_for; For Deep Mutational Scanning (DMS) of ABP1's SH3 domain at residue E16. Forward primer.
GCTGCAGAAGATAACGAANNNACCTTTGTGGAAAATGAC	DMS_ABP1_L17_for; For Deep Mutational Scanning (DMS) of ABP1's SH3 domain at residue L17. Forward primer.
GCAGAAGATAACGAACTGNNNTTTGTGGAAAATGACAAG	DMS_ABP1_T18_for; For Deep Mutational Scanning (DMS) of ABP1's SH3 domain at residue T18. Forward primer.
GAAGATAACGAACTGACCNNNGTGGAAAATGACAAG	DMS_ABP1_F19_for; For Deep Mutational Scanning (DMS) of ABP1's SH3 domain at residue F19. Forward primer.
GATAACGAACTGACCTTTNNNGAAAATGACAAGATTATC	DMS_ABP1_V20_for; For Deep Mutational Scanning (DMS) of ABP1's SH3 domain at residue V20. Forward primer.
CGAACTGACCTTTGTGNNNAATGACAAGATTATC	DMS_ABP1_E21_for; For Deep Mutational Scanning (DMS) of ABP1's SH3 domain at residue E21. Forward primer.
GAACTGACCTTTGTGGAANNNGACAAGATTATC	DMS_ABP1_N22_for; For Deep Mutational Scanning (DMS) of ABP1's SH3 domain at residue N22. Forward primer.
CTGACCTTTGTGGAAAATNNNAAGATTATCAATATTG	DMS_ABP1_D23_for; For Deep Mutational Scanning (DMS) of ABP1's SH3 domain at residue D23. Forward primer.
CCTTTGTGGAAAATGACNNNATTATCAATATTG	DMS_ABP1_K24_for; For Deep Mutational Scanning (DMS) of ABP1's SH3 domain at residue K24. Forward primer.
GTGGAAAATGACAAGNNNATCAATATTGAATTTGTC	DMS_ABP1_I25_for; For Deep Mutational Scanning (DMS) of ABP1's SH3 domain at residue I25. Forward primer.
GTGGAAAATGACAAGATTNNNAATATTGAATTTGTCGAC	DMS_ABP1_I26_for; For Deep Mutational Scanning (DMS) of ABP1's SH3 domain at residue I26. Forward primer.

GAAAATGACAAGATTATCNNNATTGAATTTGTCGACG	DMS_ABP1_N27_for; For Deep Mutational Scanning (DMS) of ABP1's SH3 domain at residue N27. Forward primer.
GACAAGATTATCAATNNNGAATTTGTCGACGATGAC	DMS_ABP1_I28_for; For Deep Mutational Scanning (DMS) of ABP1's SH3 domain at residue I28. Forward primer.
GACAAGATTATCAATATTNNNTTTGTCGACGATGACTGG	DMS_ABP1_E29_for; For Deep Mutational Scanning (DMS) of ABP1's SH3 domain at residue E29. Forward primer.
GATTATCAATATTGAANNNGTCGACGATGACTGGTGG	DMS_ABP1_F30_for; For Deep Mutational Scanning (DMS) of ABP1's SH3 domain at residue F30. Forward primer.
CAATATTGAATTTNNNGACGATGACTGGTGGC	DMS_ABP1_V31_for; For Deep Mutational Scanning (DMS) of ABP1's SH3 domain at residue V31. Forward primer.
CAATATTGAATTTGTCNNNGATGACTGGTGGCTAGGG	DMS_ABP1_D32_for; For Deep Mutational Scanning (DMS) of ABP1's SH3 domain at residue D32. Forward primer.
GAATTTGTCGACNNNGACTGGTGGCTAGGGG	DMS_ABP1_D33_for; For Deep Mutational Scanning (DMS) of ABP1's SH3 domain at residue D33. Forward primer.
GAATTTGTCGACGATNNNTGGTGGCTAGGGGAAC	DMS_ABP1_D34_for; For Deep Mutational Scanning (DMS) of ABP1's SH3 domain at residue D34. Forward primer.
GAATTTGTCGACGATGACNNNTGGCTAGGGGAACTAGAG	DMS_ABP1_W35_for; For Deep Mutational Scanning (DMS) of ABP1's SH3 domain at residue W35. Forward primer.
GTCGACGATGACTGGNNNCTAGGGGAACTAGAG	DMS_ABP1_W36_for; For Deep Mutational Scanning (DMS) of ABP1's SH3 domain at residue W36. Forward primer.
GTCGACGATGACTGGTGGNNNGGGGAACTAGAGAAAGAC	DMS_ABP1_L37_for; For Deep Mutational Scanning (DMS) of ABP1's SH3 domain at residue L37. Forward primer.
GACGATGACTGGTGGCTANNNGAACTAGAGAAAGACGGC	DMS_ABP1_G38_for; For Deep Mutational Scanning (DMS) of ABP1's SH3 domain at residue G38. Forward primer.
GATGACTGGTGGCTAGGGNNNCTAGAGAAAGACGGCTC	DMS_ABP1_E39_for; For Deep Mutational Scanning (DMS) of ABP1's SH3 domain at residue E39. Forward primer.
GACTGGTGGCTAGGGGAANNNGAGAAAGACGGCTC	DMS_ABP1_L40_for; For Deep Mutational Scanning (DMS) of ABP1's SH3 domain at residue L40. Forward primer.
GGTGGCTAGGGGAACTANNNAAAGACGGCTCAAAAGG	DMS_ABP1_E41_for; For Deep Mutational Scanning (DMS) of ABP1's SH3 domain at residue E41. Forward primer.
GGCTAGGGGAACTAGAGNNNGACGGCTCAAAAGGTCTC	DMS_ABP1_K42_for; For Deep Mutational Scanning (DMS) of ABP1's SH3 domain at residue K42. Forward primer.
CTAGGGGAACTAGAGAAANNNGGCTCAAAAGGTCTCTTC	DMS_ABP1_D43_for; For Deep Mutational Scanning (DMS) of ABP1's SH3 domain at residue D43. Forward primer.
GGGGAACTAGAGAAAGACNNNTCAAAAGGTCTCTTCCCC	DMS_ABP1_G44_for; For Deep Mutational Scanning (DMS) of ABP1's SH3 domain at residue G44. Forward primer.

GAACTAGAGAAAGACGGCNNNAAAGGTCTCTTCCCCAGC	DMS_ABP1_S45_for; For Deep Mutational Scanning (DMS) of ABP1's SH3 domain at residue S45. Forward primer.
CTAGAGAAAGACGGCTCANNNGGTCTCTTCCCCAGC	DMS_ABP1_K46_for; For Deep Mutational Scanning (DMS) of ABP1's SH3 domain at residue K46. Forward primer.
GAGAAAGACGGCTCAAAANNNCTCTTCCCCAGC	DMS_ABP1_G47_for; For Deep Mutational Scanning (DMS) of ABP1's SH3 domain at residue G47. Forward primer.
GACGGCTCAAAAGGTNNNTTCCCCAGCAATTATGTG	DMS_ABP1_L48_for; For Deep Mutational Scanning (DMS) of ABP1's SH3 domain at residue L48. Forward primer.
GACGGCTCAAAAGGTCTCNNNCCCAGCAATTATGTGTC	DMS_ABP1_F49_for; For Deep Mutational Scanning (DMS) of ABP1's SH3 domain at residue F49. Forward primer.
GGCTCAAAAGGTCTCTTCNNNAGCAATTATGTGTCTTTG	DMS_ABP1_P50_for; For Deep Mutational Scanning (DMS) of ABP1's SH3 domain at residue P50. Forward primer.
CAAAAGGTCTCTTCCCCNNNAATTATGTGTCTTTGGGC	DMS_ABP1_S51_for; For Deep Mutational Scanning (DMS) of ABP1's SH3 domain at residue S51. Forward primer.
GGTCTCTTCCCCAGCNNNTATGTGTCTTTGGGCAAC	DMS_ABP1_N52_for; For Deep Mutational Scanning (DMS) of ABP1's SH3 domain at residue N52. Forward primer.
GGTCTCTTCCCCAGCAATNNNGTGTCTTTGGGCAACGGC	DMS_ABP1_Y53_for; For Deep Mutational Scanning (DMS) of ABP1's SH3 domain at residue Y53. Forward primer.
CTCTTCCCCAGCAATTATNNNTCTTTGGGCAACGGCGG	DMS_ABP1_V54_for; For Deep Mutational Scanning (DMS) of ABP1's SH3 domain at residue V54. Forward primer.
CCCCAGCAATTATGTGNNNTTGGGCAACGGCGGTGGC	DMS_ABP1_S55_for; For Deep Mutational Scanning (DMS) of ABP1's SH3 domain at residue S55. Forward primer.
CCCAGCAATTATGTGTCTNNNGGCAACGGCGGTGGCGG	DMS_ABP1_L56_for; For Deep Mutational Scanning (DMS) of ABP1's SH3 domain at residue L56. Forward primer.
GCAATTATGTGTCTTTGNNNAACGGCGGTGGCGGATC	DMS_ABP1_G57_for; For Deep Mutational Scanning (DMS) of ABP1's SH3 domain at residue G57. Forward primer.
GTGTCTTTGGGCNNNGGCGGTGGCGGATCAGG	DMS_ABP1_N58_for; For Deep Mutational Scanning (DMS) of ABP1's SH3 domain at residue N58. Forward primer.
CTTTATGCTTCCGGCTCGTATG	DMS PCR1 pUC19 rev; For Deep Mutational Scanning (DMS) of SH3 domains in pUC19. Common reverse primer.
CAGCTGCCATCAAGAAGCTCT	Oligo-F to amplify the SH3 mutant library in puc19 vector with its homology arms for CRISPR insertion at the genomic ABP1 SH3
CCCCATATTTTGGGACACGGC	Oligo-R to amplify the SH3 mutant library in puc19 vector with its homology arms for CRISPR insertion at the genomic ABP1 SH3

CCATACGAGCACATTACGGGAAAAAGCCAAAGGAAAAT	Forward to amplify SH3-DMS pool in ABP1/YCR088W locus
CTTGACTGAGCGACTGAGGTCCTGATCCGCCACCGCC	Reverse to amplify SH3-DMS pool in ABP1/YCR088W locus
TAACTTACGGAGTCGCTCTACGCAAGTGTTCCCATACGAGCACATTACGGG	Forward to do Row-Column-PCR with index 1 for Illumina Sequencing (DB-BC_R_1)
TAACTTACGGAGTCGCTCTACGAGGACATTCCCATACGAGCACATTACGGG	Forward to do Row-Column-PCR with index 2 for Illumina Sequencing (DB-BC_R_2)
TAACTTACGGAGTCGCTCTACGCACTAATGGCCATACGAGCACATTACGGG	Forward to do Row-Column-PCR with index 3 for Illumina Sequencing (DB-BC_R_3)
TAACTTACGGAGTCGCTCTACGAGCCTGATGCCATACGAGCACATTACGGG	Forward to do Row-Column-PCR with index 4 for Illumina Sequencing (DB-BC_R_4)
TAACTTACGGAGTCGCTCTACGTTACGCTAACCATACGAGCACATTACGGG	Forward to do Row-Column-PCR with index 5 for Illumina Sequencing (DB-BC_R_5)
TAACTTACGGAGTCGCTCTACGACTCTCCGTCCATACGAGCACATTACGGG	Forward to do Row-Column-PCR with index 6 for Illumina Sequencing (DB-BC_R_6)
TAACTTACGGAGTCGCTCTACGGTCGATGCACCATACGAGCACATTACGGG	Forward to do Row-Column-PCR with index 7 for Illumina Sequencing (DB-BC_R_7)
TAACTTACGGAGTCGCTCTACGACGGGAATTCCATACGAGCACATTACGGG	Forward to do Row-Column-PCR with index 8 for Illumina Sequencing (DB-BC_R_8)
GGATGGGATTCTTTAGGTCCTGCAAGTGTTCCTTGACTGAGCGACTGAGG	Reverse to do Row-Column-PCR with index 1 for Illumina Sequencing (DB-BC_C_1)
GGATGGGATTCTTTAGGTCCTGAGGACATTCCTTGACTGAGCGACTGAGG	Reverse to do Row-Column-PCR with index 2 for Illumina Sequencing (DB-BC_C_2)
GGATGGGATTCTTTAGGTCCTGCACTAATGGCTTGACTGAGCGACTGAGG	Reverse to do Row-Column-PCR with index 3 for Illumina Sequencing (DB-BC_C_3)
GGATGGGATTCTTTAGGTCCTGAGCCTGATGCTTGACTGAGCGACTGAGG	Reverse to do Row-Column-PCR with index 4 for Illumina Sequencing (DB-BC_C_4)
GGATGGGATTCTTTAGGTCCTGTTACGCTAACTTGACTGAGCGACTGAGG	Reverse to do Row-Column-PCR with index 5 for Illumina Sequencing (DB-BC_C_5)
GGATGGGATTCTTTAGGTCCTGACTCTCCGTCTTGACTGAGCGACTGAGG	Reverse to do Row-Column-PCR with index 6 for Illumina Sequencing (DB-BC_C_6)
GGATGGGATTCTTTAGGTCCTGGTCGATGCACTTGACTGAGCGACTGAGG	Reverse to do Row-Column-PCR with index 7 for Illumina Sequencing (DB-BC_C_7)
GGATGGGATTCTTTAGGTCCTGACGGGAATTCTTGACTGAGCGACTGAGG	Reverse to do Row-Column-PCR with index 8 for Illumina Sequencing (DB-BC_C_8)

Supplementary Table 2.
List of strains used in this study

Strain description	Source	Identifier
S. cerevisiae : Yeast Protein Interactome Collection DHFR[1,2] (background BY4741)	(Tarassov et al., 2008) Horizon	#YSC5849
S. cerevisiae : Yeast Protein Interactome Collection DHFR[3] (background BY4742)	(Tarassov et al., 2008) Horizon	#YSC5849
S. cerevisiae : Yeast knockout Collection (background BY4741)	Horizon	#YSC1053
S. cerevisiae : Sla1-GFP from the yeast GFP collection (background BY4741)	Thermo Fisher Scientific	#95702
S. cerevisiae: Abp1-linker-DHFR[1,2]-Linker-1XFLAG (background BY4741)	This study	N/A
S. cerevisiae: Abp1-KO-NATMX4 (background BY4741)	This study	N/A
S. cerevisiae: Abp1-SH3-deleted-linker-DHFR[1,2]-Linker-1XFLAG (background BY4741)	This study	N/A
S. cerevisiae: Abp1-SH3-swapped-Abp1SH3-linker-DHFR[1,2]-Linker-1XFLAG (background BY4741)	This study	N/A
S. cerevisiae: Abp1-SH3-swapped-Bbc1SH3-linker-DHFR[1,2]-Linker-1XFLAG (background BY4741)	This study	N/A
S. cerevisiae: Abp1-SH3-swapped-Bem1SH3-1-linker-DHFR[1,2]-Linker-1XFLAG (background BY4741)	This study	N/A
S. cerevisiae: Abp1-SH3-swapped-Bem1SH3-2-linker-DHFR[1,2]-Linker-1XFLAG (background BY4741)	This study	N/A
S. cerevisiae: Abp1-SH3-swapped-Boi1SH3-linker-DHFR[1,2]-Linker-1XFLAG (background BY4741)	This study	N/A
S. cerevisiae: Abp1-SH3-swapped-Boi2SH3-linker-DHFR[1,2]-Linker-1XFLAG (background BY4741)	This study	N/A
S. cerevisiae: Abp1-SH3-swapped-Bud14SH3-linker-DHFR[1,2]-Linker-1XFLAG (background BY4741)	This study	N/A
S. cerevisiae: Abp1-SH3-swapped-Bzz1SH3-1-linker-DHFR[1,2]-Linker-1XFLAG (background BY4741)	This study	N/A
S. cerevisiae: Abp1-SH3-swapped-Bzz1SH3-2-linker-DHFR[1,2]-Linker-1XFLAG (background BY4741)	This study	N/A
S. cerevisiae: Abp1-SH3-swapped-Cdc25SH3-linker-DHFR[1,2]-Linker-1XFLAG (background BY4741)	This study	N/A
S. cerevisiae: Abp1-SH3-swapped-CTTNSH3-linker-DHFR[1,2]-Linker-1XFLAG (background BY4741)	This study	N/A
S. cerevisiae: Abp1-SH3-swapped-Cyk3SH3-linker-DHFR[1,2]-Linker-1XFLAG (background BY4741)	This study	N/A
S. cerevisiae: Abp1-SH3-swapped-DBNLSH3-linker-DHFR[1,2]-Linker-1XFLAG (background BY4741)	This study	N/A
S. cerevisiae: Abp1-SH3-swapped-Fus1SH3-linker-DHFR[1,2]-Linker-1XFLAG (background BY4741)	This study	N/A
S. cerevisiae: Abp1-SH3-swapped-HCLS1SH3-linker-DHFR[1,2]-Linker-1XFLAG (background BY4741)	This study	N/A
S. cerevisiae: Abp1-SH3-swapped-Hof1SH3-linker-DHFR[1,2]-Linker-1XFLAG (background BY4741)	This study	N/A
S. cerevisiae: Abp1-SH3-swapped-Hse1SH3-linker-DHFR[1,2]-Linker-1XFLAG (background BY4741)	This study	N/A
S. cerevisiae: Abp1-SH3-swapped-Lsb1SH3-linker-DHFR[1,2]-Linker-1XFLAG (background BY4741)	This study	N/A
S. cerevisiae: Abp1-SH3-swapped-Lsb3SH3-linker-DHFR[1,2]-Linker-1XFLAG (background BY4741)	This study	N/A

S. cerevisiae: Abp1-SH3-swapped-Myo3SH3-linker-DHFR[1,2]-Linker-1XFLAG (background BY4741)	This study	N/A
S. cerevisiae: Abp1-SH3-swapped-Myo5SH3-linker-DHFR[1,2]-Linker-1XFLAG (background BY4741)	This study	N/A
S. cerevisiae: Abp1-SH3-swapped-Nbp2SH3-linker-DHFR[1,2]-Linker-1XFLAG (background BY4741)	This study	N/A
S. cerevisiae: Abp1-SH3-swapped-Pex13SH3-linker-DHFR[1,2]-Linker-1XFLAG (background BY4741)	This study	N/A
S. cerevisiae: Abp1-SH3-swapped-Pin3SH3-linker-DHFR[1,2]-Linker-1XFLAG (background BY4741)	This study	N/A
S. cerevisiae: Abp1-SH3-swapped-Rvs167SH3-linker-DHFR[1,2]-Linker-1XFLAG (background BY4741)	This study	N/A
S. cerevisiae: Abp1-SH3-swapped-Sdc25SH3-linker-DHFR[1,2]-Linker-1XFLAG (background BY4741)	This study	N/A
S. cerevisiae: Abp1-SH3-swapped-Sho1SH3-linker-DHFR[1,2]-Linker-1XFLAG (background BY4741)	This study	N/A
S. cerevisiae: Abp1-SH3-swapped-Sla1SH3-1-linker-DHFR[1,2]-Linker-1XFLAG (background BY4741)	This study	N/A
S. cerevisiae: Abp1-SH3-swapped-Sla1SH3-2-linker-DHFR[1,2]-Linker-1XFLAG (background BY4741)	This study	N/A
S. cerevisiae: Abp1-SH3-swapped-Sla1SH3-2-linker-DHFR[1,2]-Linker-1XFLAG (background BY4741)	This study	N/A
S. cerevisiae: Abp1-SH3-swapped-Ysc84SH3-linker-DHFR[1,2]-Linker-1XFLAG (background BY4741)	This study	N/A
S. cerevisiae: Bbc1-linker-DHFR[1,2]-Linker-1XFLAG (background BY4741)	This study	N/A
S. cerevisiae: Bbc1-SH3-deleted-linker-DHFR[1,2]-Linker-1XFLAG (background BY4741)	This study	N/A
S. cerevisiae: Bem1-linker-DHFR[1,2]-Linker-1XFLAG (background BY4741)	This study	N/A
S. cerevisiae: Bem1-SH3-1-deleted-linker-DHFR[1,2]-Linker-1XFLAG (background BY4741)	This study	N/A
S. cerevisiae: Bem1-SH3-2-deleted-linker-DHFR[1,2]-Linker-1XFLAG (background BY4741)	This study	N/A
S. cerevisiae: Bem1-SH3-1-And-SH3-2-deleted-linker-DHFR[1,2]-Linker-1XFLAG (background BY4741)	This study	N/A
S. cerevisiae: Bem1-KO-NATMX4 (background BY4741)	This study	N/A
S. cerevisiae: Boi1-linker-DHFR[1,2]-Linker-1XFLAG (background BY4741)	This study	N/A
S. cerevisiae: Boi1-SH3-deleted-linker-DHFR[1,2]-Linker-1XFLAG (background BY4741)	This study	N/A
S. cerevisiae: Boi2-linker-DHFR[1,2]-Linker-1XFLAG (background BY4741)	This study	N/A
S. cerevisiae: Boi2-SH3-deleted-linker-DHFR[1,2]-Linker-1XFLAG (background BY4741)	This study	N/A
S. cerevisiae: Bud14-linker-DHFR[1,2]-Linker-1XFLAG (background BY4741)	This study	N/A
S. cerevisiae: Bud14-SH3-deleted-linker-DHFR[1,2]-Linker-1XFLAG (background BY4741)	This study	N/A
S. cerevisiae: Bzz1-linker-DHFR[1,2]-Linker-1XFLAG (background BY4741)	This study	N/A
S. cerevisiae: Bzz1-SH3-1-deleted-linker-DHFR[1,2]-Linker-1XFLAG (background BY4741)	This study	N/A

S. cerevisiae: Bzz1-SH3-2-deleted-linker-DHFR[1,2]-Linker-1XFLAG (background BY4741)	This study	N/A
S. cerevisiae: Bzz1-SH3-1-And-SH3-2-deleted-linker-DHFR[1,2]-Linker-1XFLAG (background BY4741)	This study	N/A
S. cerevisiae: Bzz1-KO-NATMX4 (background BY4741)	This study	N/A
S. cerevisiae: Cdc25-linker-DHFR[1,2]-Linker-1XFLAG (background BY4741)	This study	N/A
S. cerevisiae: Cdc25-SH3-deleted-linker-DHFR[1,2]-Linker-1XFLAG (background BY4741)	This study	N/A
S. cerevisiae: Cyk3-linker-DHFR[1,2]-Linker-1XFLAG (background BY4741)	This study	N/A
S. cerevisiae: Cyk3-SH3-deleted-linker-DHFR[1,2]-Linker-1XFLAG (background BY4741)	This study	N/A
S. cerevisiae: Fus1-linker-DHFR[1,2]-Linker-1XFLAG (background BY4741)	This study	N/A
S. cerevisiae: Fus1-SH3-deleted-linker-DHFR[1,2]-Linker-1XFLAG (background BY4741)	This study	N/A
S. cerevisiae: Hof1-linker-DHFR[1,2]-Linker-1XFLAG (background BY4741)	This study	N/A
S. cerevisiae: Hof1-SH3-deleted-linker-DHFR[1,2]-Linker-1XFLAG (background BY4741)	This study	N/A
S. cerevisiae: Hse1-linker-DHFR[1,2]-Linker-1XFLAG (background BY4741)	This study	N/A
S. cerevisiae: Hse1-SH3-deleted-linker-DHFR[1,2]-Linker-1XFLAG (background BY4741)	This study	N/A
S. cerevisiae: Lsb1-linker-DHFR[1,2]-Linker-1XFLAG (background BY4741)	This study	N/A
S. cerevisiae: Lsb1-SH3-deleted-linker-DHFR[1,2]-Linker-1XFLAG (background BY4741)	This study	N/A
S. cerevisiae: Lsb3-linker-DHFR[1,2]-Linker-1XFLAG (background BY4741)	This study	N/A
S. cerevisiae: Lsb3-SH3-deleted-linker-DHFR[1,2]-Linker-1XFLAG (background BY4741)	This study	N/A
S. cerevisiae: Myo3-linker-DHFR[1,2]-Linker-1XFLAG (background BY4741)	This study	N/A
S. cerevisiae: Myo3-SH3-deleted-linker-DHFR[1,2]-Linker-1XFLAG (background BY4741)	This study	N/A
S. cerevisiae: Myo5-linker-DHFR[1,2]-Linker-1XFLAG (background BY4741)	This study	N/A
S. cerevisiae: Myo5-SH3-deleted-linker-DHFR[1,2]-Linker-1XFLAG (background BY4741)	This study	N/A
S. cerevisiae: Nbp2-linker-DHFR[1,2]-Linker-1XFLAG (background BY4741)	This study	N/A
S. cerevisiae: Nbp2-SH3-deleted-linker-DHFR[1,2]-Linker-1XFLAG (background BY4741)	This study	N/A
S. cerevisiae: Pex13-linker-DHFR[1,2]-Linker-1XFLAG (background BY4741)	This study	N/A
S. cerevisiae: Pex13-SH3-deleted-linker-DHFR[1,2]-Linker-1XFLAG (background BY4741)	This study	N/A
S. cerevisiae: Pin3-linker-DHFR[1,2]-Linker-1XFLAG (background BY4741)	This study	N/A
S. cerevisiae: Pin3-SH3-deleted-linker-DHFR[1,2]-Linker-1XFLAG (background BY4741)	This study	N/A

S. cerevisiae: Rvs167-linker-DHFR[1,2]-Linker-1XFLAG (background BY4741)	This study	N/A
S. cerevisiae: Rvs167-SH3-deleted-linker-DHFR[1,2]-Linker-1XFLAG (background BY4741)	This study	N/A
S. cerevisiae: Sho1-linker-DHFR[1,2]-Linker-1XFLAG (background BY4741)	This study	N/A
S. cerevisiae: Sho1-SH3-deleted-linker-DHFR[1,2]-Linker-1XFLAG (background BY4741)	This study	N/A
S. cerevisiae: Sla1-linker-DHFR[1,2]-Linker-1XFLAG (background BY4741)	This study	N/A
S. cerevisiae: Sla1-KO-NATMX4 (background BY4741)	This study	N/A
S. cerevisiae: Sla1-SH3-1-deleted-linker-DHFR[1,2]-Linker-1XFLAG (background BY4741)	This study	N/A
S. cerevisiae: Sla1-SH3-2-deleted-linker-DHFR[1,2]-Linker-1XFLAG (background BY4741)	This study	N/A
S. cerevisiae: Sla1-SH3-3-deleted-linker-DHFR[1,2]-Linker-1XFLAG (background BY4741)	This study	N/A
S. cerevisiae: Sla1-SH3-1-And-SH3-2-And-SH3-3-deleted-linker-DHFR[1,2]-Linker-1XFLAG (background BY4741)	This study	N/A
S. cerevisiae: Sla1-SH3-shuffled-SH3-2|SH3-1|WT-linker-DHFR[1,2]-Linker-1XFLAG (background BY4741)	This study	N/A
S. cerevisiae: Sla1-SH3-shuffled-WT|SH3-2|SH3-2-linker-DHFR[1,2]-Linker-1XFLAG (background BY4741)	This study	N/A
S. cerevisiae: Sla1-SH3-shuffled-SH3-3|WT|SH3-1-linker-DHFR[1,2]-Linker-1XFLAG (background BY4741)	This study	N/A
S. cerevisiae: Sla1-SH3-shuffled-SH3-3|SH3-1|SH3-2-linker-DHFR[1,2]-Linker-1XFLAG (background BY4741)	This study	N/A
S. cerevisiae: Sla1-SH3-swapped-SH3Abp1|WT|WT-linker-DHFR[1,2]-Linker-1XFLAG (background BY4741)	This study	N/A
S. cerevisiae: Sla1-SH3-swapped-WT|SH3Abp1|WT-linker-DHFR[1,2]-Linker-1XFLAG (background BY4741)	This study	N/A
S. cerevisiae: Sla1-SH3-swapped-WT|WT|SH3Abp1-linker-DHFR[1,2]-Linker-1XFLAG (background BY4741)	This study	N/A
S. cerevisiae: Sla1-SH3-shuffled-SH3-3|SH3-1|D-linker-DHFR[1,2]-Linker-1XFLAG (background BY4741)	This study	N/A
S. cerevisiae: Sla1-SH3-shuffled-SH3-3|SH3-2|D-linker-DHFR[1,2]-Linker-1XFLAG (background BY4741)	This study	N/A
S. cerevisiae: Sla1-SH3-shuffled-SH3-3|SH3-2|SH3-3-linker-DHFR[1,2]-Linker-1XFLAG (background BY4741)	This study	N/A
S. cerevisiae: Sla1-SH3-shuffled-SH3-1|SH3-2|SH3-1-linker-DHFR[1,2]-Linker-1XFLAG (background BY4741)	This study	N/A
S. cerevisiae: Sla1-SH3-shuffled-SH3-1|SH3-2|SH3-2-linker-DHFR[1,2]-Linker-1XFLAG (background BY4741)	This study	N/A
S. cerevisiae: Sla1-SH3-shuffled-SH3-1|SH3-1|SH3-3-linker-DHFR[1,2]-Linker-1XFLAG (background BY4741)	This study	N/A
S. cerevisiae: Sla1-SH3-shuffled-SH3-1|SH3-1|D-linker-DHFR[1,2]-Linker-1XFLAG (background BY4741)	This study	N/A
S. cerevisiae: Sla1-SH3-shuffled-SH3-1|D|D-linker-DHFR[1,2]-Linker-1XFLAG (background BY4741)	This study	N/A
S. cerevisiae: Sla1-SH3-shuffled-SH3-2|SH3-2|SH3-3-linker-DHFR[1,2]-Linker-1XFLAG (background BY4741)	This study	N/A
S. cerevisiae: Sla1-SH3-shuffled-SH3-1|SH3-3|D-linker-DHFR[1,2]-Linker-1XFLAG (background BY4741)	This study	N/A

S. cerevisiae: Sla1-SH3-shuffled-SH3-1|SH3-3|SH3-3-linker-DHFR[1,2]-Linker-1XFLAG (background BY4741)	This study	N/A
S. cerevisiae: Sla1-SH3-shuffled-D|SH3-2|D-linker-DHFR[1,2]-Linker-1XFLAG (background BY4741)	This study	N/A
S. cerevisiae: Sla1-SH3-shuffled-SH3-1|D|SH3-2-linker-DHFR[1,2]-Linker-1XFLAG (background BY4741)	This study	N/A
S. cerevisiae: Sla1-SH3-shuffled-SH3-1*|SH3-2|SH3-3-linker-DHFR[1,2]-Linker-1XFLAG (background BY4741)	This study	N/A
S. cerevisiae: Sla1-SH3-shuffled-SH3-1|SH3-2*|SH3-3-linker-DHFR[1,2]-Linker-1XFLAG (background BY4741)	This study	N/A
S. cerevisiae: Sla1-SH3-shuffled-SH3-1|SH3-2|SH3-3*-linker-DHFR[1,2]-Linker-1XFLAG (background BY4741)	This study	N/A
S. cerevisiae: Sla1-SH3-shuffled-D|D|SH3-1-linker-DHFR[1,2]-Linker-1XFLAG (background BY4741)	This study	N/A
S. cerevisiae: Sla1-SH3-shuffled-D|D|SH3-3-linker-DHFR[1,2]-Linker-1XFLAG (background BY4741)	This study	N/A
S. cerevisiae: Sla1-SH3-shuffled-SH3-2|SH3-2|D-linker-DHFR[1,2]-Linker-1XFLAG (background BY4741)	This study	N/A
S. cerevisiae: Sla1-SH3-shuffled-D|SH3-2|SH3-2-linker-DHFR[1,2]-Linker-1XFLAG (background BY4741)	This study	N/A
S. cerevisiae: Sla1-SH3-shuffled-D|SH3-2|SH3-1-linker-DHFR[1,2]-Linker-1XFLAG (background BY4741)	This study	N/A
S. cerevisiae: Sla1-SH3-1-deleted-GFP (background BY4741)	This study	N/A
S. cerevisiae: Sla1-SH3-2-deleted-GFP (background BY4741)	This study	N/A
S. cerevisiae: Sla1-SH3-3-deleted-GFP (background BY4741)	This study	N/A
S. cerevisiae: Sla1-SH3-1-And-SH3-2-And-SH3-3-deleted-GFP (background BY4741)	This study	N/A
S. cerevisiae: Sla1-SH3-shuffled-SH3-2|SH3-1|WT-GFP (background BY4741)	This study	N/A
S. cerevisiae: Sla1-SH3-shuffled-WT|SH3-2|SH3-2-GFP (background BY4741)	This study	N/A
S. cerevisiae: Sla1-SH3-shuffled-SH3-3|WT|SH3-1-GFP (background BY4741)	This study	N/A
S. cerevisiae:Sla1-SH3-shuffled-SH3-2|SH3-1|SH3-3-GFP (background BY4741)	This study	N/A
S. cerevisiae:Sla1-SH3-shuffled-SH3-3|SH3-1|SH3-2-GFP (background BY4741)	This study	N/A
S. cerevisiae: Abp1-DMSlibrary-Pos1-NNN-linker-DHFR[1,2]-Linker-1XFLAG (background BY4741)	This study	N/A
S. cerevisiae: Abp1-DMSlibrary-Pos2-NNN-linker-DHFR[1,2]-Linker-1XFLAG (background BY4741)	This study	N/A
S. cerevisiae: Abp1-DMSlibrary-Pos3-NNN-linker-DHFR[1,2]-Linker-1XFLAG (background BY4741)	This study	N/A
S. cerevisiae: Abp1-DMSlibrary-Pos4-NNN-linker-DHFR[1,2]-Linker-1XFLAG (background BY4741)	This study	N/A
S. cerevisiae: Abp1-DMSlibrary-Pos5-NNN-linker-DHFR[1,2]-Linker-1XFLAG (background BY4741)	This study	N/A
S. cerevisiae: Abp1-DMSlibrary-Pos6-NNN-linker-DHFR[1,2]-Linker-1XFLAG (background BY4741)	This study	N/A
S. cerevisiae: Abp1-DMSlibrary-Pos7-NNN-linker-DHFR[1,2]-Linker-1XFLAG (background BY4741)	This study	N/A
S. cerevisiae: Abp1-DMSlibrary-Pos8-NNN-linker-DHFR[1,2]-Linker-1XFLAG (background BY4741)	This study	N/A

S. cerevisiae: Abp1-DMSlibrary-Pos9-NNN-linker-DHFR[1,2]-Linker-1XFLAG (background BY4741)	This study	N/A
S. cerevisiae: Abp1-DMSlibrary-Pos10-NNN-linker-DHFR[1,2]-Linker-1XFLAG (background BY4741)	This study	N/A
S. cerevisiae: Abp1-DMSlibrary-Pos11-NNN-linker-DHFR[1,2]-Linker-1XFLAG (background BY4741)	This study	N/A
S. cerevisiae: Abp1-DMSlibrary-Pos12-NNN-linker-DHFR[1,2]-Linker-1XFLAG (background BY4741)	This study	N/A
S. cerevisiae: Abp1-DMSlibrary-Pos13-NNN-linker-DHFR[1,2]-Linker-1XFLAG (background BY4741)	This study	N/A
S. cerevisiae: Abp1-DMSlibrary-Pos14-NNN-linker-DHFR[1,2]-Linker-1XFLAG (background BY4741)	This study	N/A
S. cerevisiae: Abp1-DMSlibrary-Pos15-NNN-linker-DHFR[1,2]-Linker-1XFLAG (background BY4741)	This study	N/A
S. cerevisiae: Abp1-DMSlibrary-Pos16-NNN-linker-DHFR[1,2]-Linker-1XFLAG (background BY4741)	This study	N/A
S. cerevisiae: Abp1-DMSlibrary-Pos17-NNN-linker-DHFR[1,2]-Linker-1XFLAG (background BY4741)	This study	N/A
S. cerevisiae: Abp1-DMSlibrary-Pos18-NNN-linker-DHFR[1,2]-Linker-1XFLAG (background BY4741)	This study	N/A
S. cerevisiae: Abp1-DMSlibrary-Pos19-NNN-linker-DHFR[1,2]-Linker-1XFLAG (background BY4741)	This study	N/A
S. cerevisiae: Abp1-DMSlibrary-Pos20-NNN-linker-DHFR[1,2]-Linker-1XFLAG (background BY4741)	This study	N/A
S. cerevisiae: Abp1-DMSlibrary-Pos21-NNN-linker-DHFR[1,2]-Linker-1XFLAG (background BY4741)	This study	N/A
S. cerevisiae: Abp1-DMSlibrary-Pos22-NNN-linker-DHFR[1,2]-Linker-1XFLAG (background BY4741)	This study	N/A
S. cerevisiae: Abp1-DMSlibrary-Pos23-NNN-linker-DHFR[1,2]-Linker-1XFLAG (background BY4741)	This study	N/A
S. cerevisiae: Abp1-DMSlibrary-Pos24-NNN-linker-DHFR[1,2]-Linker-1XFLAG (background BY4741)	This study	N/A
S. cerevisiae: Abp1-DMSlibrary-Pos25-NNN-linker-DHFR[1,2]-Linker-1XFLAG (background BY4741)	This study	N/A
S. cerevisiae: Abp1-DMSlibrary-Pos26-NNN-linker-DHFR[1,2]-Linker-1XFLAG (background BY4741)	This study	N/A
S. cerevisiae: Abp1-DMSlibrary-Pos27-NNN-linker-DHFR[1,2]-Linker-1XFLAG (background BY4741)	This study	N/A
S. cerevisiae: Abp1-DMSlibrary-Pos28-NNN-linker-DHFR[1,2]-Linker-1XFLAG (background BY4741)	This study	N/A
S. cerevisiae: Abp1-DMSlibrary-Pos29-NNN-linker-DHFR[1,2]-Linker-1XFLAG (background BY4741)	This study	N/A
S. cerevisiae: Abp1-DMSlibrary-Pos30-NNN-linker-DHFR[1,2]-Linker-1XFLAG (background BY4741)	This study	N/A
S. cerevisiae: Abp1-DMSlibrary-Pos31-NNN-linker-DHFR[1,2]-Linker-1XFLAG (background BY4741)	This study	N/A
S. cerevisiae: Abp1-DMSlibrary-Pos32-NNN-linker-DHFR[1,2]-Linker-1XFLAG (background BY4741)	This study	N/A
S. cerevisiae: Abp1-DMSlibrary-Pos33-NNN-linker-DHFR[1,2]-Linker-1XFLAG (background BY4741)	This study	N/A
S. cerevisiae: Abp1-DMSlibrary-Pos34-NNN-linker-DHFR[1,2]-Linker-1XFLAG (background BY4741)	This study	N/A
S. cerevisiae: Abp1-DMSlibrary-Pos35-NNN-linker-DHFR[1,2]-Linker-1XFLAG (background BY4741)	This study	N/A

S. cerevisiae: Abp1-DMSlibrary-Pos36-NNN-linker-DHFR[1,2]-Linker-1XFLAG (background BY4741)	This study	N/A
S. cerevisiae: Abp1-DMSlibrary-Pos37-NNN-linker-DHFR[1,2]-Linker-1XFLAG (background BY4741)	This study	N/A
S. cerevisiae: Abp1-DMSlibrary-Pos38-NNN-linker-DHFR[1,2]-Linker-1XFLAG (background BY4741)	This study	N/A
S. cerevisiae: Abp1-DMSlibrary-Pos39-NNN-linker-DHFR[1,2]-Linker-1XFLAG (background BY4741)	This study	N/A
S. cerevisiae: Abp1-DMSlibrary-Pos40-NNN-linker-DHFR[1,2]-Linker-1XFLAG (background BY4741)	This study	N/A
S. cerevisiae: Abp1-DMSlibrary-Pos41-NNN-linker-DHFR[1,2]-Linker-1XFLAG (background BY4741)	This study	N/A
S. cerevisiae: Abp1-DMSlibrary-Pos42-NNN-linker-DHFR[1,2]-Linker-1XFLAG (background BY4741)	This study	N/A
S. cerevisiae: Abp1-DMSlibrary-Pos43-NNN-linker-DHFR[1,2]-Linker-1XFLAG (background BY4741)	This study	N/A
S. cerevisiae: Abp1-DMSlibrary-Pos44-NNN-linker-DHFR[1,2]-Linker-1XFLAG (background BY4741)	This study	N/A
S. cerevisiae: Abp1-DMSlibrary-Pos45-NNN-linker-DHFR[1,2]-Linker-1XFLAG (background BY4741)	This study	N/A
S. cerevisiae: Abp1-DMSlibrary-Pos46-NNN-linker-DHFR[1,2]-Linker-1XFLAG (background BY4741)	This study	N/A
S. cerevisiae: Abp1-DMSlibrary-Pos47-NNN-linker-DHFR[1,2]-Linker-1XFLAG (background BY4741)	This study	N/A
S. cerevisiae: Abp1-DMSlibrary-Pos48-NNN-linker-DHFR[1,2]-Linker-1XFLAG (background BY4741)	This study	N/A
S. cerevisiae: Abp1-DMSlibrary-Pos49-NNN-linker-DHFR[1,2]-Linker-1XFLAG (background BY4741)	This study	N/A
S. cerevisiae: Abp1-DMSlibrary-Pos50-NNN-linker-DHFR[1,2]-Linker-1XFLAG (background BY4741)	This study	N/A
S. cerevisiae: Abp1-DMSlibrary-Pos51-NNN-linker-DHFR[1,2]-Linker-1XFLAG (background BY4741)	This study	N/A
S. cerevisiae: Abp1-DMSlibrary-Pos52-NNN-linker-DHFR[1,2]-Linker-1XFLAG (background BY4741)	This study	N/A
S. cerevisiae: Abp1-DMSlibrary-Pos53-NNN-linker-DHFR[1,2]-Linker-1XFLAG (background BY4741)	This study	N/A
S. cerevisiae: Abp1-DMSlibrary-Pos54-NNN-linker-DHFR[1,2]-Linker-1XFLAG (background BY4741)	This study	N/A
S. cerevisiae: Abp1-DMSlibrary-Pos55-NNN-linker-DHFR[1,2]-Linker-1XFLAG (background BY4741)	This study	N/A
S. cerevisiae: Abp1-DMSlibrary-Pos56-NNN-linker-DHFR[1,2]-Linker-1XFLAG (background BY4741)	This study	N/A
S. cerevisiae: Abp1-DMSlibrary-Pos57-NNN-linker-DHFR[1,2]-Linker-1XFLAG (background BY4741)	This study	N/A
S. cerevisiae: Abp1-DMSlibrary-Pos58-NNN-linker-DHFR[1,2]-Linker-1XFLAG (background BY4741)	This study	N/A
S. cerevisiae: YAL005C/SSA1-linker-DHFR[3] (background BY4742)	This study	N/A
S. cerevisiae: YAR014C/BUD14-linker-DHFR[3] (background BY4742)	This study	N/A
S. cerevisiae: YBL007C/SLA1-linker-DHFR[3] (background BY4742)	This study	N/A
S. cerevisiae: YBR009C/HHF1-linker-DHFR[3] (background BY4742)	This study	N/A
S. cerevisiae: YBR034C/HMT1-linker-DHFR[3] (background BY4742)	This study	N/A
S. cerevisiae: YCR002C/CDC10-linker-DHFR[3] (background BY4742)	This study	N/A
S. cerevisiae: YCR008W/SAT4-linker-DHFR[3] (background BY4742)	This study	N/A

S. cerevisiae: YCR009C/RVS161-linker-DHFR[3] (background BY4742)	This study	N/A
S. cerevisiae: YCR088W/ABP1-linker-DHFR[3] (background BY4742)	This study	N/A
S. cerevisiae: YDL229W/SSB1-linker-DHFR[3] (background BY4742)	This study	N/A
S. cerevisiae: YDR129C/SAC6-linker-DHFR[3] (background BY4742)	This study	N/A
S. cerevisiae: YDR243C/PRP28-linker-DHFR[3] (background BY4742)	This study	N/A
S. cerevisiae: YDR388W/RVS167-linker-DHFR[3] (background BY4742)	This study	N/A
S. cerevisiae: YDR416W/SYF1-linker-DHFR[3] (background BY4742)	This study	N/A
S. cerevisiae: YDR507C/GIN4-linker-DHFR[3] (background BY4742)	This study	N/A
S. cerevisiae: YEL037C/RAD23-linker-DHFR[3] (background BY4742)	This study	N/A
S. cerevisiae: YER149C/PEA2-linker-DHFR[3] (background BY4742)	This study	N/A
S. cerevisiae: YFL010C/WWM1-linker-DHFR[3] (background BY4742)	This study	N/A
S. cerevisiae: YFR004W/RPN11-linker-DHFR[3] (background BY4742)	This study	N/A
S. cerevisiae: YFR010W/UBP6-linker-DHFR[3] (background BY4742)	This study	N/A
S. cerevisiae: YFR028C/CDC14-linker-DHFR[3] (background BY4742)	This study	N/A
S. cerevisiae: YGL060W/YBP2-linker-DHFR[3] (background BY4742)	This study	N/A
S. cerevisiae: YGL070C/RPB9-linker-DHFR[3] (background BY4742)	This study	N/A
S. cerevisiae: YGL106W/MLC1-linker-DHFR[3] (background BY4742)	This study	N/A
S. cerevisiae: YGL197W/MDS3-linker-DHFR[3] (background BY4742)	This study	N/A
S. cerevisiae: YGL207W/SPT16-linker-DHFR[3] (background BY4742)	This study	N/A
S. cerevisiae: YGR052W/FMP48-linker-DHFR[3] (background BY4742)	This study	N/A
S. cerevisiae: YGR168C/PEX35-linker-DHFR[3] (background BY4742)	This study	N/A
S. cerevisiae: YGR229C/SMI1-linker-DHFR[3] (background BY4742)	This study	N/A
S. cerevisiae: YHR064C/SSZ1-linker-DHFR[3] (background BY4742)	This study	N/A
S. cerevisiae: YHR140W/YHR140W-linker-DHFR[3] (background BY4742)	This study	N/A
S. cerevisiae: YHR185C/PFS1-linker-DHFR[3] (background BY4742)	This study	N/A
S. cerevisiae: YIL021W/RPB3-linker-DHFR[3] (background BY4742)	This study	N/A
S. cerevisiae: YIL033C/BCY1-linker-DHFR[3] (background BY4742)	This study	N/A
S. cerevisiae: YIL156W/UBP7-linker-DHFR[3] (background BY4742)	This study	N/A
S. cerevisiae: YIR003W/AIM21-linker-DHFR[3] (background BY4742)	This study	N/A
S. cerevisiae: YJL128C/PBS2-linker-DHFR[3] (background BY4742)	This study	N/A
S. cerevisiae: YJL210W/PEX2-linker-DHFR[3] (background BY4742)	This study	N/A
S. cerevisiae: YKL117W/SBA1-linker-DHFR[3] (background BY4742)	This study	N/A
S. cerevisiae: YKL129C/MYO3-linker-DHFR[3] (background BY4742)	This study	N/A
S. cerevisiae: YKR001C/VPS1-linker-DHFR[3] (background BY4742)	This study	N/A
S. cerevisiae: YLL048C/YBT1-linker-DHFR[3] (background BY4742)	This study	N/A
S. cerevisiae: YLR078C/BOS1-linker-DHFR[3] (background BY4742)	This study	N/A
S. cerevisiae: YLR113W/HOG1-linker-DHFR[3] (background BY4742)	This study	N/A
S. cerevisiae: YLR229C/CDC42-linker-DHFR[3] (background BY4742)	This study	N/A
S. cerevisiae: YLR310C/CDC25-linker-DHFR[3] (background BY4742)	This study	N/A
S. cerevisiae: YLR314C/CDC3-linker-DHFR[3] (background BY4742)	This study	N/A
S. cerevisiae: YLR320W/MMS22-linker-DHFR[3] (background BY4742)	This study	N/A
S. cerevisiae: YLR435W/TSR2-linker-DHFR[3] (background BY4742)	This study	N/A
S. cerevisiae: YML131W/YML131W-linker-DHFR[3] (background BY4742)	This study	N/A
S. cerevisiae: YMR001C/CDC5-linker-DHFR[3] (background BY4742)	This study	N/A
S. cerevisiae: YMR216C/SKY1-linker-DHFR[3] (background BY4742)	This study	N/A
S. cerevisiae: YNL020C/ARK1-linker-DHFR[3] (background BY4742)	This study	N/A
S. cerevisiae: YNL026W/SAM50-linker-DHFR[3] (background BY4742)	This study	N/A

S. cerevisiae: YNL030W/HHF2-linker-DHFR[3] (background BY4742)	This study	N/A
S. cerevisiae: YNL073W/MSK1-linker-DHFR[3] (background BY4742)	This study	N/A
S. cerevisiae: YNL094W/APP1-linker-DHFR[3] (background BY4742)	This study	N/A
S. cerevisiae: YNL243W/SLA2-linker-DHFR[3] (background BY4742)	This study	N/A
S. cerevisiae: YNL244C/SUI1-linker-DHFR[3] (background BY4742)	This study	N/A
S. cerevisiae: YNL271C/BNI1-linker-DHFR[3] (background BY4742)	This study	N/A
S. cerevisiae: YNL315C/ATP11-linker-DHFR[3] (background BY4742)	This study	N/A
S. cerevisiae: YOL133W/HRT1-linker-DHFR[3] (background BY4742)	This study	N/A
S. cerevisiae: YOR035C/SHE4-linker-DHFR[3] (background BY4742)	This study	N/A
S. cerevisiae: YOR057W/SGT1-linker-DHFR[3] (background BY4742)	This study	N/A
S. cerevisiae: YOR091W/TMA46-linker-DHFR[3] (background BY4742)	This study	N/A
S. cerevisiae: YPL235W/RVB2-linker-DHFR[3] (background BY4742)	This study	N/A
S. cerevisiae: YNL078W/NIS1-linker-DHFR[3] (background BY4742)	This study	N/A
S. cerevisiae: YDL028C/MPS1-linker-DHFR[3] (background BY4742)	This study	N/A
S. cerevisiae: YIR006C/PAN1-linker-DHFR[3] (background BY4742)	This study	N/A
S. cerevisiae: YCL008C/STP22-linker-DHFR[3] (background BY4742)	This study	N/A
S. cerevisiae: YIL159W/BNR1-linker-DHFR[3] (background BY4742)	This study	N/A
S. cerevisiae: YHR023W/MYO1-linker-DHFR[3] (background BY4742)	This study	N/A
S. cerevisiae: YGR092W/DBF2-linker-DHFR[3] (background BY4742)	This study	N/A

Supplementary Table 3.
List of media used in this study

Media name	Composition
YPD liquid	1% Yeast extract, 2% Tryptone, 2% Glucose
YPD solid	1% Yeast extract, 2% Tryptone, 2% Glucose, 2% Agar
PCA_MTX -ade-lys-met	0.67% Yeast nitrogen base without amonium sulfate without amino acids, 2% Glucose, 200ug/mL methotrexate
PCA_MTX -ade-lys-met solid	0.67% Yeast nitrogen base without amonium sulfate without amino acids, 2% Glucose, 2.5% Agar noble, 200ug/mL methotrexate
10X PCA Drop-out -ade-lys-met	0.4g Tryptophane, 0.3g Tyrosine, 0.5g Phenylalanine, 1.0g Glutamic acid (monosodium salt), 1.0g Asparagine, 1.5g Valine, 2.0g Threonine, 3.75g Serine, 0.2g Uracile, 0.2g Histidine, 0.2g Arginine, 0.6g Leucine
1.34g/L Drop-out complet	0.5g Adenine, 2.0g Arginine, 2.0g, Aspartic acid, 2.0g Glutamic acid, 2.0g Histidine, 10.0g Leucine, 2.0g Lysine, 2.0g Methionine, 2.0g Phenylalanine, 2.0g Serine, 2.0g Threonine, 2.0g Tryptophane, 2.0g Tyrosine, 2.0g Uracile, 2.0g Valine
1.27g/L Drop-out -trp	0.5g Adenine, 2.0g Arginine, 2.0g, Aspartic acid, 2.0g Glutamic acid, 2.0g Histidine, 10.0g Leucine, 2.0g Lysine, 2.0g Methionine, 2.0g Phenylalanine, 2.0g Serine, 2.0g Threonine, 2.0g Tyrosine, 2.0g Uracile, 2.0g Valine
SC Ammonium	0.174% Yeast nitrogen base without ammonium sulfate without amino acids, 0.5% Ammonium sulfate, 2% Glucose, XXX appropriate drop-out
SC MSG	0.174% Yeast nitrogen base without ammonium sulfate without amino acids, 0.1% Glutamic acid (monosodium salt), 2% Glucose, XXX appropriate drop-out
SC MSG pH 6.0 liquid	0.174% Yeast nitrogen base without ammonium sulfate without amino acids, 0.1% Glutamic acid (monosodium salt), 2% Glucose, XXX appropriate drop-out, 1% Succinic acid, 0.6% Sodium hydroxide
SC MSG pH 6.0 solid	0.174% Yeast nitrogen base without ammonium sulfate without amino acids, 0.1% Glutamic acid (monosodium salt), 2% Glucose, XXX appropriate drop-out, 1% Succinic acid, 0.6% Sodium hydroxide, 2% Agar
SC Ammonium pH 6.0 solid	0.174% Yeast nitrogen base without ammonium sulfate without amino acids, 0.5% Ammonium sulfate, 2% Glucose, 0.0134% Drop-out complet, 1% Succinic acid, 0.6% Sodium hydroxide, 2% Agar
SC Ammonium pH 6.0 + 0.05µM Cycloheximide	0.174% Yeast nitrogen base without ammonium sulfate without amino acids, 0.5% Ammonium sulfate, 2% Glucose, 0.0134% Drop-out complet, 1% Succinic acid, 0.6% Sodium hydroxide, 2% Agar, 2% DMSO, 0.05µM Cycloheximide
SC Ammonium pH 6.0 + 0.2µM Cycloheximide	0.174% Yeast nitrogen base without ammonium sulfate without amino acids, 0.5% Ammonium sulfate, 2% Glucose, 0.0134% Drop-out complet, 1% Succinic acid, 0.6% Sodium hydroxide, 2% Agar, 2% DMSO, 0.2µM Cycloheximide
SC Ammonium pH 6.0 + 0.5µM Cycloheximide	0.174% Yeast nitrogen base without ammonium sulfate without amino acids, 0.5% Ammonium sulfate, 2% Glucose, 0.0134% Drop-out complet, 1% Succinic acid, 0.6% Sodium hydroxide, 2% Agar, 2% DMSO, 0.5µM Cycloheximide
SC Ammonium pH 6.0 + 2mM Caffein	0.174% Yeast nitrogen base without ammonium sulfate without amino acids, 0.5% Ammonium sulfate, 2% Glucose, 0.0134% Drop-out complet, 1% Succinic acid, 0.6% Sodium hydroxide, 2% Agar, 2mM Caffein
SC Ammonium pH 6.0 + 10mM Caffein	0.174% Yeast nitrogen base without ammonium sulfate without amino acids, 0.5% Ammonium sulfate, 2% Glucose, 0.0134% Drop-out complet, 1% Succinic acid, 0.6% Sodium hydroxide, 2% Agar, 10mM Caffein

SC Ammonium pH 6.0 + 25mM Hydroxyurea	0.174% Yeast nitrogen base without ammonium sulfate without amino acids, 0.5% Ammonium sulfate, 2% Glucose, 0.0134% Drop-out complet, 1% Succinic acid, 0.6% Sodium hydroxide, 2% Agar, 50mM Hydroxyurea
SC Ammonium pH 6.0 + 0.004% MMS	0.174% Yeast nitrogen base without ammonium sulfate without amino acids, 0.5% Ammonium sulfate, 2% Glucose, 0.0134% Drop-out complet, 1% Succinic acid, 0.6% Sodium hydroxide, 2% Agar, 0.004% MMS
SC Ammonium pH 6.0 + 0.02% MMS	0.174% Yeast nitrogen base without ammonium sulfate without amino acids, 0.5% Ammonium sulfate, 2% Glucose, 0.0134% Drop-out complet, 1% Succinic acid, 0.6% Sodium hydroxide, 2% Agar, 0.02% MMS
SC Ammonium pH 6.0 + 0.04% MMS	0.174% Yeast nitrogen base without ammonium sulfate without amino acids, 0.5% Ammonium sulfate, 2% Glucose, 0.0134% Drop-out complet, 1% Succinic acid, 0.6% Sodium hydroxide, 2% Agar, 0.04% MMS
SC Ammonium pH 6.0 + 10nM Rapamycin	0.174% Yeast nitrogen base without ammonium sulfate without amino acids, 0.5% Ammonium sulfate, 2% Glucose, 0.0134% Drop-out complet, 1% Succinic acid, 0.6% Sodium hydroxide, 2% Agar, 2% DMSO, 10nM Rapamycin
SC Ammonium pH 6.0 + 50nM Rapamycin	0.174% Yeast nitrogen base without ammonium sulfate without amino acids, 0.5% Ammonium sulfate, 2% Glucose, 0.0134% Drop-out complet, 1% Succinic acid, 0.6% Sodium hydroxide, 2% Agar, 2% DMSO, 50nM Rapamycin
SC Ammonium pH 6.0 + 0.25M NaCl	0.174% Yeast nitrogen base without ammonium sulfate without amino acids, 0.5% Ammonium sulfate, 2% Glucose, 0.0134% Drop-out complet, 1% Succinic acid, 0.6% Sodium hydroxide, 2% Agar, 0.25M NaCl
SC Ammonium pH 6.0 + 1M NaCl	0.174% Yeast nitrogen base without ammonium sulfate without amino acids, 0.5% Ammonium sulfate, 2% Glucose, 0.0134% Drop-out complet, 1% Succinic acid, 0.6% Sodium hydroxide, 2% Agar, 1M NaCl
SC Ammonium pH 6.0 + 10µg/ml Hygromycin	0.174% Yeast nitrogen base without ammonium sulfate without amino acids, 0.5% Ammonium sulfate, 2% Glucose, 0.0134% Drop-out complet, 1% Succinic acid, 0.6% Sodium hydroxide, 2% Agar, 10µg/ml Hygromycin
SC Ammonium pH 6.0 + 50µg/ml Hygromycin	0.174% Yeast nitrogen base without ammonium sulfate without amino acids, 0.5% Ammonium sulfate, 2% Glucose, 0.0134% Drop-out complet, 1% Succinic acid, 0.6% Sodium hydroxide, 2% Agar, 50µg/ml Hygromycin
SC Ammonium pH 6.0 + 100µg/ml Hygromycin	0.174% Yeast nitrogen base without ammonium sulfate without amino acids, 0.5% Ammonium sulfate, 2% Glucose, 0.0134% Drop-out complet, 1% Succinic acid, 0.6% Sodium hydroxide, 2% Agar, 100µg/ml Hygromycin
SC Ammonium pH 6.0, 2% Galactose	0.174% Yeast nitrogen base without ammonium sulfate without amino acids, 0.5% Ammonium sulfate, 2% Galactose, 0.0134% Drop-out complet, 1% Succinic acid, 0.6% Sodium hydroxide, 2% Agar
SC Ammonium pH 6.0, 2% Raffinose	0.174% Yeast nitrogen base without ammonium sulfate without amino acids, 0.5% Ammonium sulfate, 2% Raffinose, 0.0134% Drop-out complet, 1% Succinic acid, 0.6% Sodium hydroxide, 2% Agar
SC Ammonium pH 6.0, 2% Glycerol	0.174% Yeast nitrogen base without ammonium sulfate without amino acids, 0.5% Ammonium sulfate, 2% Glycerol, 0.0134% Drop-out complet, 1% Succinic acid, 0.6% Sodium hydroxide, 2% Agar
SC Ammonium pH 6.0 + 1mM CuSO4	0.174% Yeast nitrogen base without ammonium sulfate without amino acids, 0.5% Ammonium sulfate, 2% Glucose, 0.0134% Drop-out complet, 1% Succinic acid, 0.6% Sodium hydroxide, 2% Agar, 1mM CuSO4
SC Ammonium pH 6.0 + 5mM CuSO4	0.174% Yeast nitrogen base without ammonium sulfate without amino acids, 0.5% Ammonium sulfate, 2% Glucose, 0.0134% Drop-out complet, 1% Succinic acid, 0.6% Sodium hydroxide, 2% Agar, 5mM CuSO4

SC Ammonium pH 6.0 + 0.01µM Nystatin	0.174% Yeast nitrogen base without ammonium sulfate without amino acids, 0.5% Ammonium sulfate, 2% Glucose, 0.0134% Drop-out complet, 1% Succinic acid, 0.6% Sodium hydroxide, 2% Agar, 2% DMSO, 0.01µM Nystatin
SC Ammonium pH 6.0 + 0.1µM Nystatin	0.174% Yeast nitrogen base without ammonium sulfate without amino acids, 0.5% Ammonium sulfate, 2% Glucose, 0.0134% Drop-out complet, 1% Succinic acid, 0.6% Sodium hydroxide, 2% Agar, 2% DMSO, 0.1µM Nystatin
SC Ammonium pH 6.0 + 0.1µM Bathophenanthroline	0.174% Yeast nitrogen base without ammonium sulfate without amino acids, 0.5% Ammonium sulfate, 2% Glucose, 0.0134% Drop-out complet, 1% Succinic acid, 0.6% Sodium hydroxide, 2% Agar, 2% Ethanol, 0.1µM Bathophenanthroline
SC Ammonium pH 6.0 + 1µM Bathophenanthroline	0.174% Yeast nitrogen base without ammonium sulfate without amino acids, 0.5% Ammonium sulfate, 2% Glucose, 0.0134% Drop-out complet, 1% Succinic acid, 0.6% Sodium hydroxide, 2% Agar, 2% Ethanol, 1µM Bathophenanthroline
SC Ammonium pH 6.0 + 10µM Bathophenanthroline	0.174% Yeast nitrogen base without ammonium sulfate without amino acids, 0.5% Ammonium sulfate, 2% Glucose, 0.0134% Drop-out complet, 1% Succinic acid, 0.6% Sodium hydroxide, 2% Agar, 2% Ethanol, 10µM Bathophenanthroline
SC Ammonium pH 6.0 + 2% Ethanol	0.174% Yeast nitrogen base without ammonium sulfate without amino acids, 0.5% Ammonium sulfate, 2% Glucose, 0.0134% Drop-out complet, 1% Succinic acid, 0.6% Sodium hydroxide, 2% Agar, 2% Ethanol
SC Ammonium pH 6.0 + 6% Ethanol	0.174% Yeast nitrogen base without ammonium sulfate without amino acids, 0.5% Ammonium sulfate, 2% Glucose, 0.0134% Drop-out complet, 1% Succinic acid, 0.6% Sodium hydroxide, 2% Agar, 6% Ethanol
SC Ammonium pH 6.0 + 8% Ethanol	0.174% Yeast nitrogen base without ammonium sulfate without amino acids, 0.5% Ammonium sulfate, 2% Glucose, 0.0134% Drop-out complet, 1% Succinic acid, 0.6% Sodium hydroxide, 2% Agar, 8% Ethanol
SC Ammonium pH 6.0 + 100mM CaCl2	0.174% Yeast nitrogen base without ammonium sulfate without amino acids, 0.5% Ammonium sulfate, 2% Glucose, 0.0134% Drop-out complet, 1% Succinic acid, 0.6% Sodium hydroxide, 2% Agar, 250mM CaCl2
SC Ammonium pH 6.0 + 250mM CaCl2	0.174% Yeast nitrogen base without ammonium sulfate without amino acids, 0.5% Ammonium sulfate, 2% Glucose, 0.0134% Drop-out complet, 1% Succinic acid, 0.6% Sodium hydroxide, 2% Agar, 750mM CaCl2
SC Ammonium pH 6.0 + 500M CaCl2	0.174% Yeast nitrogen base without ammonium sulfate without amino acids, 0.5% Ammonium sulfate, 2% Glucose, 0.0134% Drop-out complet, 1% Succinic acid, 0.6% Sodium hydroxide, 2% Agar, 1M CaCl2
SC Ammonium pH 3.0	0.174% Yeast nitrogen base without ammonium sulfate without amino acids, 0.5% Ammonium sulfate, 2% Glucose, 0.0134% Drop-out complet, 2% Agar
SC Ammonium pH 6.0 + 1mM Sorbitol	0.174% Yeast nitrogen base without ammonium sulfate without amino acids, 0.5% Ammonium sulfate, 2% Glucose, 0.0134% Drop-out complet, 1% Succinic acid, 0.6% Sodium hydroxide, 2% Agar, 1mM Sorbitol
SC Ammonium pH 6.0 + 5mM Sorbitol	0.174% Yeast nitrogen base without ammonium sulfate without amino acids, 0.5% Ammonium sulfate, 2% Glucose, 0.0134% Drop-out complet, 1% Succinic acid, 0.6% Sodium hydroxide, 2% Agar, 5mM Sorbitol
SC Ammonium pH 6.0 + 10mM Sorbitol	0.174% Yeast nitrogen base without ammonium sulfate without amino acids, 0.5% Ammonium sulfate, 2% Glucose, 0.0134% Drop-out complet, 1% Succinic acid, 0.6% Sodium hydroxide, 2% Agar, 10mM Sorbitol
SC Ammonium pH 6.0 + 10µM Cadmium Chloride	0.174% Yeast nitrogen base without ammonium sulfate without amino acids, 0.5% Ammonium sulfate, 2% Glucose, 0.0134% Drop-out complet, 1% Succinic acid, 0.6% Sodium hydroxide, 2% Agar, 10µM Cadmium Chloride

SC Ammonium pH 6.0 + 50µM Cadmium Chloride	0.174% Yeast nitrogen base without ammonium sulfate without amino acids, 0.5% Ammonium sulfate, 2% Glucose, 0.0134% Drop-out complet, 1% Succinic acid, 0.6% Sodium hydroxide, 2% Agar, 50µM Cadmium Chloride
SC Ammonium pH 6.0 + 100µM Cadmium Chloride	0.174% Yeast nitrogen base without ammonium sulfate without amino acids, 0.5% Ammonium sulfate, 2% Glucose, 0.0134% Drop-out complet, 1% Succinic acid, 0.6% Sodium hydroxide, 2% Agar, 100µM Cadmium Chloride
SC Ammonium pH 6.0 + 2% DMSO	0.174% Yeast nitrogen base without ammonium sulfate without amino acids, 0.5% Ammonium sulfate, 2% Glucose, 0.0134% Drop-out complet, 1% Succinic acid, 0.6% Sodium hydroxide, 2% Agar, 2% DMSO
SC Ammonium pH 6.0 + 6% DMSO	0.174% Yeast nitrogen base without ammonium sulfate without amino acids, 0.5% Ammonium sulfate, 2% Glucose, 0.0134% Drop-out complet, 1% Succinic acid, 0.6% Sodium hydroxide, 2% Agar, 6% DMSO
SC Ammonium pH 6.0 + 8% DMSO	0.174% Yeast nitrogen base without ammonium sulfate without amino acids, 0.5% Ammonium sulfate, 2% Glucose, 0.0134% Drop-out complet, 1% Succinic acid, 0.6% Sodium hydroxide, 2% Agar, 8% DMSO
SC Ammonium pH 6.0 + 50µM Menadione	0.174% Yeast nitrogen base without ammonium sulfate without amino acids, 0.5% Ammonium sulfate, 2% Glucose, 0.0134% Drop-out complet, 1% Succinic acid, 0.6% Sodium hydroxide, 2% Agar, 50µM Menadione
SC Ammonium pH 6.0 + 100µM Menadione	0.174% Yeast nitrogen base without ammonium sulfate without amino acids, 0.5% Ammonium sulfate, 2% Glucose, 0.0134% Drop-out complet, 1% Succinic acid, 0.6% Sodium hydroxide, 2% Agar, 100µM Menadione
SC Ammonium pH 6.0 + 200µM Menadione	0.174% Yeast nitrogen base without ammonium sulfate without amino acids, 0.5% Ammonium sulfate, 2% Glucose, 0.0134% Drop-out complet, 1% Succinic acid, 0.6% Sodium hydroxide, 2% Agar, 200µM Menadione
SC Ammonium pH 6.0 + 10µM Nitroquinoline	0.174% Yeast nitrogen base without ammonium sulfate without amino acids, 0.5% Ammonium sulfate, 2% Glucose, 0.0134% Drop-out complet, 1% Succinic acid, 0.6% Sodium hydroxide, 2% Agar, 2% DMSO, 10µM Nitroquinoline
SC Ammonium pH 6.0 + 50µM Nitroquinoline	0.174% Yeast nitrogen base without ammonium sulfate without amino acids, 0.5% Ammonium sulfate, 2% Glucose, 0.0134% Drop-out complet, 1% Succinic acid, 0.6% Sodium hydroxide, 2% Agar, 2% DMSO, 50µM Nitroquinoline
SC Ammonium pH 6.0 + 0.01% SDS	0.174% Yeast nitrogen base without ammonium sulfate without amino acids, 0.5% Ammonium sulfate, 2% Glucose, 0.0134% Drop-out complet, 1% Succinic acid, 0.6% Sodium hydroxide, 2% Agar, 0.01% SDS
SC Ammonium pH 6.0 + 0.02% SDS	0.174% Yeast nitrogen base without ammonium sulfate without amino acids, 0.5% Ammonium sulfate, 2% Glucose, 0.0134% Drop-out complet, 1% Succinic acid, 0.6% Sodium hydroxide, 2% Agar, 0.02% SDS
SC Ammonium pH 6.0 + 0.05% SDS	0.174% Yeast nitrogen base without ammonium sulfate without amino acids, 0.5% Ammonium sulfate, 2% Glucose, 0.0134% Drop-out complet, 1% Succinic acid, 0.6% Sodium hydroxide, 2% Agar, 0.05% SDS

Supplementary References :

1. Tonikian, R. *et al.* Bayesian modeling of the yeast SH3 domain interactome predicts spatiotemporal dynamics of endocytosis proteins. *PLoS Biol.* **7**, e1000218 (2009).

2. Stark, C. *et al.* BioGRID: a general repository for interaction datasets. *Nucleic Acids Res.* **34**, D535–9 (2006).

3. Garcia, B., Stollar, E. J. & Davidson, A. R. The importance of conserved features of yeast actin-binding protein 1 (Abp1p): the conditional nature of essentiality. *Genetics* **191**, 1199–1211 (2012).

4. Stollar, E. J. *et al.* Structural, functional, and bioinformatic studies demonstrate the crucial role of an extended peptide binding site for the SH3 domain of yeast Abp1p. *J. Biol. Chem.* **284**, 26918–26927 (2009).
