# Supplementary material for: Protein context shapes the specificity of SH3 domain-mediated interactions in vivo
Source: Nat Commun. 2021 Mar 12;12:1597. doi: 10.1038/s41467-021-21873-2 (PMC7954794; doi:10.1038/s41467-021-21873-2)
Supplement: Supplementary file 6 — Description of Additional Supplementary Files [file 41467_2021_21873_MOESM6_ESM.docx]

Description of additional supplementary files

Title: Supplementary Data 1.

Description: Raw data of every DHFR-PCA experiments (solid and liquid) performed for this study. Linked with Figs 1, 2, 3, S1, S2, S3 and S4.

Title: Supplementary Data 2.

Description: Raw data of every growth experiments performed for this study. Linked with Figs 1, S2, and S5.

Title: Supplementary Data 3.

Description: Raw data of MS (DDA and DIA) and CD experiments performed for this study. Linked with Figs 5 and S6.
