# Supplementary material for: Protein context shapes the specificity of SH3 domain-mediated interactions in vivo
Source: Nat Commun. 2021 Mar 12;12:1597. doi: 10.1038/s41467-021-21873-2 (PMC7954794; doi:10.1038/s41467-021-21873-2)
Supplement: Supplementary file 7 — Reporting Summary [file 41467_2021_21873_MOESM7_ESM.pdf]

nature research

Corresponding author(s): Christian Landry and Nicolas Bisson

Last updated by author(s): Feb 10, 2021

Reporting Summary

Nature Research wishes to improve the reproducibility of the work that we publish. This form provides structure for consistency and transparency in reporting. For further information on Nature Research policies, see our Editorial Policies and the Editorial Policy Checklist.

Statistics

For all statistical analyses, confirm that the following items are present in the figure legend, table legend, main text, or Methods section.

n/a	Confirmed	
☐	☒	The exact sample size (*n*) for each experimental group/condition, given as a discrete number and unit of measurement
☐	☒	A statement on whether measurements were taken from distinct samples or whether the same sample was measured repeatedly
☐	☒	The statistical test(s) used AND whether they are one- or two-sided<br>*Only common tests should be described solely by name; describe more complex techniques in the Methods section.*
☐	☒	A description of all covariates tested
☒	☐	A description of any assumptions or corrections, such as tests of normality and adjustment for multiple comparisons
☐	☒	A full description of the statistical parameters including central tendency (e.g. means) or other basic estimates (e.g. regression coefficient) AND variation (e.g. standard deviation) or associated estimates of uncertainty (e.g. confidence intervals)
☐	☒	For null hypothesis testing, the test statistic (e.g. *F*, *t*, *r*) with confidence intervals, effect sizes, degrees of freedom and *P* value noted<br>*Give P values as exact values whenever suitable.*
☒	☐	For Bayesian analysis, information on the choice of priors and Markov chain Monte Carlo settings
☒	☐	For hierarchical and complex designs, identification of the appropriate level for tests and full reporting of outcomes
☒	☐	Estimates of effect sizes (e.g. Cohen's *d*, Pearson's *r*), indicating how they were calculated

Our web collection on statistics for biologists contains articles on many of the points above.

Software and code

Policy information about availability of computer code

Data collection	DMS sequencing results were obtained using a MiSeq sequencer that uses proprietary Illumina software to generate fastqfiles. Other softwares used for data collection: Zen Blue (Carl Zeiss Microscopy, version 2.3.69.1000) and Amersham Imager 600 analysis (for WB, GE Healthcare).
Data analysis	The custom code for all data analysis is linked in the code availability section of the paper (https://github.com/Landrylab/DionneEtal2020).<br><br>Softwares, algorithms and packages: R (R version >3.0, https://www.r-project.org/), Mutfunc (sequence alignment, http://www.mutfunc.com/), Gitter Package for R (yeast colony analysis, http://omarwagih.github.io/gitter/), PEAR (sequencing analysis, v0.9.6, https://cme.h-its.org/exelixis/web/software/pear/), Pysam (sequencing analysis, v0.14.1), Samtools (sequencing analysis, v1.9, http://samtools.sourceforge.net/), Bowtie2 (sequencing analysis, v2.3.5, http://bowtie-bio.sourceforge.net/bowtie2/index.shtml), GraphPad Prism 7 (FP data analysis, https://www.graphpad.com/), DSSP web server (surface accessibilities of amino acids, https://www3.cmbi.umcn.nl/xssp/), Ensembl REST API (ortholog analysis, https://rest.ensembl.org/), HMMER (ortholog domains analysis, v3.3, https://www.ebi.ac.uk/Tools/hmmer/), MAFFT (sequence alignment, L-INS-i, https://mafft.cbrc.jp/alignment/software/), bio3d package for R (domain sequence analysis, http://thegrantlab.org/bio3d/index.php) and dendextend package for R (tree comparison analysis, https://github.com/talgalili/dendextend).<br><br>MS Data analysis: ProHits (v.4.0, http://prohitsms.comProhits_download/list.php), ProteoWizard (v3.0.4468, http://proteowizard.sourceforge.net/), Mascot (version 2.3.02, RRID: SCR_014322), Comet (version 2012.02 rev.0, http://comet-ms.sourceforge.net/), Trans-Proteomic Pipeline (v4.6 OCCUPY rev 3, http://tools.proteomecenter.org/software.php), MS-GFDB (Beta version 1.0072 (6/30/2014), https://msgfplus.github.io/msgfplus/index.html), Independent Acquisition analysis: MSPLIT-DIA (v.1.0, http://proteomics.ucsd.edu/software-tools/msplit-dia/) and Significance Analysis of INTeractome analysis (SAINTexpress version 3.6.1, http://saint-apms.sourceforge.net/).<br><br>Microscopy analysis: Python 6.0.3., YeastSpotter (Dec 12, 2018, https://github.com/alexxijielu/yeast_segmentation), Trackpy (v0.4.2, https://

pypi.org/project/trackpy/), Scikit-Image (v0.16.2, https://pypi.org/project/scikit-image/) and SciPy (v1.4.1, https://pypi.org/project/scipy/).

For manuscripts utilizing custom algorithms or software that are central to the research but not yet described in published literature, software must be made available to editors and reviewers. We strongly encourage code deposition in a community repository (e.g. GitHub). See the Nature Research guidelines for submitting code & software for further information.

Data

Policy information about availability of data

All manuscripts must include a data availability statement. This statement should provide the following information, where applicable:

- Accession codes, unique identifiers, or web links for publicly available datasets
- A list of figures that have associated raw data
- A description of any restrictions on data availability

Accession codes, unique identifiers, or web links for publicly available datasets: SMART V.8.0 (http://smart.embl-heidelberg.de/, RRID:SCR_005026), PDB (PDB:2RPN, 10.2210/pdb2RPN/pdb), BioGRID (version 3.5.16, https://thebiogrid.org/, RRID:SCR_007393), RefSeq (v. 57 (01/30/2013), NCBI, https://www.ncbi.nlm.nih.gov/refseq/), Common mass spectrometry contaminants (Max Planck Institute, http://141.61.102.106:8080/share.cgi?ssid=0f2gfuB), Global Proteome Machine (https://www.thegpm.org/crap/index.html), Ensembl Compara (https://www.ensembl.org/info/genome/compara/index.html), NCBI (NP_035009.3, https://www.ncbi.nlm.nih.gov/protein/NP_035009.3) and Uniprot (O55033, https://www.uniprot.org/uniprot/O55033).

A list of figures that have associated raw data: Figures 1-5 and Supplementary Figures 1-6. For every figure panels corresponding to new results, the associated data are in the Source Data file. In addition, large datasets corresponding to the raw data of the high-throughput screens or MS experiments are also available with the manuscript as Supplementary Data 1-3.

The MS datasets generated during this study are available at MassIVE (http://massive.ucsd.edu) MSV000085092 (DIA SWATH MS) and MSV000085093 (DDA MS). The files can be accessed using the following links: ftp://MSV000085092@massive.ucsd.edu and ftp://MSV000085093@massive.ucsd.edu. The deep sequencing datasets generated during this study are available at NCBI as BioProjects; (https://www.ncbi.nlm.nih.gov/biosample/14752885) SAMN14752885 (Abp1-Hua2 DMS reference condition), (https://www.ncbi.nlm.nih.gov/biosample/14752886) SAMN14752886 (Abp1-Lsb3 DMS reference condition), (https://www.ncbi.nlm.nih.gov/biosample/14752887) SAMN14752887 (Abp1-Hua2 DMS DHFR-PCA condition) and (https://www.ncbi.nlm.nih.gov/biosample/14752888) SAMN14752888 (Abp1-Lsb3 DMS DHFR-PCA condition).

Field-specific reporting

Please select the one below that is the best fit for your research. If you are not sure, read the appropriate sections before making your selection.

☒ Life sciences ☐ Behavioural & social sciences ☐ Ecological, evolutionary & environmental sciences

For a reference copy of the document with all sections, see nature.com/documents/nr-reporting-summary-flat.pdf

Life sciences study design

All studies must disclose on these points even when the disclosure is negative.

Sample size	Sample size for statistical analysis were based on our biological data. All statistical analysis were performed with a minimum of n = 3. For Figure 1C-D and Supplementary Figure S2G, the random distribution was calculated with n =10 000. This number is much higher than the biological sample sizes in the boxplot, allowing us to be confident that there will be no random/stochastic variation that would affect the results generated in these analyses.
Data exclusions	In all DHFR-PCA screens on solid media, Bait-Prey pairs with colony growth that was detected for less than two replicates were excluded. For the growth screens on solid media, strains with high variance between plate replicates were excluded. In yeast microscopy experiments, cells were filtered out based on anomalies (e.g. circularity) to remove poorly detected cells. In addition, cells that were out of focus were also filtered out.
Replication	Protein-Protein interaction detection was assessed in 4 biological replicates for all DHFR-PCA screens on solid media (Figures 1B. 2B, D, E. 4B, D. S2C, F and S5A). For the growth screens on solid media, colony growth was followed in 12 biological replicates (Figures 1E-F. S2D and S5B). The DMS experiments were performed in two biological replicates in the reference and DHFR-PCA conditions (Figures 3B-D. S3A, B, E, G). The yeast microscopy experiments were performed multiple times with a total of Sla1-GFP foci followed of: n = 3286 for Sla1 1|2|3, n = 473 for Sla1 D|D|D, n = 755 for Sla1 D|2|3, n = 1530 for Sla1 1|D|3, n = 1734 for Sla1 1|2|D, n = 686 for Sla1 2|1|3, n = 963 for Sla1 1|3|2, n = 652 for Sla1 3|2|1, n = 745 for Sla1 2|3|1 and n = 994 Sla1 3|1|2 (Figures 5F, G, H. S5F-I). The DDA and DIA MS experiments (for SAINT and SWATH analysis) were both performed in two biological replicates (Figure 5A-C). FP binding assays were done in biological triplicates (each binding assay was also assessed in technical duplicate, Figures 5D. S6E-F). Phase separation experiments in low salt concentration were executed in 7 biological replicates (Figure 5E) and in 3 biological replicates in other conditions (Figure S7A-C). The validation of protein interaction alterations following SH3 deletion by a liquid DHFR-PCA screen was performed once (Figure S1D-E). WB Protein expression levels of yeast baits were either validated once (Figures S1F. S5C) or twice (Figures S2A. S3C). The growth of Abp1 SH3-swapped strains in liquid media containing hygromycin was assessed in 3 biological replicates (Figure S2E). The liquid DHFR-PCA validation assay of selected Abp1 SH3 mutants was performed in 4 biological replicates (Figure S3F). The Co-immunoprecipitation WB experiments with NCK2-PAK and NCK2-P130CAS were also done in 4 biological replicates (Figure S6A-D). Finally, the CD analysis were performed in 3 biological replicates (Figure S6G).
Randomization	Strain positions on solid plates were randomly attributed for all DHFR-PCA and growth screens to limit possible plate effects. For the other smaller scale experiments, randomization was not performed.

Blinding	Because the different strains were positioned randomly on plates (containing 1536 colonies each) for all the high-throughput screens (DHFR-PCA and growth), the positive hits were discovered by the investigators only at the end of the analysis pipeline. Thus, the subjective judgement of the investigators couldn't affect the final results of the DHFR-PCA and growth screens. Data analysis was also performed in a systematic way for all yeast microscopy and MS raw data, with the final resluts being determined independently of the investigator judgement. For the other smaller scale experiments with less data, blinding was not performed.

Reporting for specific materials, systems and methods

We require information from authors about some types of materials, experimental systems and methods used in many studies. Here, indicate whether each material, system or method listed is relevant to your study. If you are not sure if a list item applies to your research, read the appropriate section before selecting a response.

Materials & experimental systems

n/a	Involved in the study
☐	☒ Antibodies
☐	☒ Eukaryotic cell lines
☒	☐ Palaeontology and archaeology
☒	☐ Animals and other organisms
☒	☐ Human research participants
☒	☐ Clinical data
☒	☐ Dual use research of concern

Methods

n/a	Involved in the study
☒	☐ ChIP-seq
☒	☐ Flow cytometry
☒	☐ MRI-based neuroimaging

Antibodies

Antibodies used	M2-HRP (A8592, Sigma), rabbit anti-FLAG (F7425, Sigma), PAK (SC-881, Santa Cruz), p130cas/BCAR1 (SC-860, Santa Cruz), actin (8H10D10, Cell Signaling Technology), anti-mouse HRP (7076, Cell Signaling Technology) and anti-rabbit HRP (7074S, Cell Signaling Technology). The dilutions used are described in the Methods section of the manuscript.
Validation	As described by suppliers:<br>αPAK Antibody (C-19) is a rabbit polyclonal IgG; 200 μg/ml. Epitope mapping at the C-terminus of αPAK of rat origin. Reactivity: Avian, Bovine, Dog, Horse, Human, Rat and Pig. 62 publications used this antibody for either WB, IF, IHC or IP (https://www.citeab.com/antibodies/821239-sc-881-pak-antibody-c-19).<br>p130 Cas Antibody (C-20) is a rabbit polyclonal IgG; 200 μg/ml. Epitope mapping near the C-terminus of p130 Cas of rat origin. Reactivity: Bovine, Dog, Horse, Human, Mouse, Rat. 36 publications used this antibody for either WB or IP (https://www.citeab.com/antibodies/816718-sc-860-p130-cas-antibody-c-20).<br>β-Actin (8H10D10) Mouse mAb detects endogenous levels of total β-actin protein. Due to the high sequence identity between the cytoplasmic actin isoforms, β-actin and cytoplasmic γ-actin, this antibody may cross-react with cytoplasmic γ-actin. It does not cross-react with α-skeletal, α-cardiac, α-vascular smooth, or γ-enteric smooth muscle isoforms. Application Key: WB, IP, IHC, ChIP, IF, Flow Cytometry and ELISA-Peptide. Species Cross-Reactivity Key: Human, Mouse, Rat, Hamster, Monkey, Virus, Mink, Chicken, D.melanogaster, Xenopus, Zebrafish, Bovine, Dog, Pig, S. cerevisiae, C. elegans, Horse, All Species Expected.

Eukaryotic cell lines

Policy information about cell lines

Cell line source(s)	Human cell HEK293T, ATCC:CRL-3216
Authentication	The cell line was not authenticated.
Mycoplasma contamination	The cell line was not tested for mycoplasma contamination,
Commonly misidentified lines (See ICLAC register)	No misidentified cell lines used.
